# Trends over 48 years in a one-number index of survival for all cancers combined, England and Wales (1971–2018): a population-based registry study

**DOI:** 10.1016/j.lanepe.2025.101385

**Published:** 2025-08-13

**Authors:** Michel P. Coleman, Melissa Matz, Pamela Minicozzi, Veronica Di Carlo, Dyfed Huws, Stephanie Smits, Jon Shelton, Claudia Allemani

**Affiliations:** a Cancer Survival Group, London School of Hygiene and Tropical Medicine, Keppel Street, London, WC1E 7HT, UK; b Cancer Division, University College London Hospitals NHS Foundation Trust, 250 Euston Road, London, NW1 2PG, UK; c Welsh Cancer Intelligence and Surveillance Unit, 2 Capital Quarter, Tyndall Street, Cardiff, CF10 4BZ, UK; d Head of Cancer Intelligence, Cancer Research UK, 2 Redman Place, London, E20 1JQ, UK

**Keywords:** Cancer Survival Index, Trends, England, Wales

## Abstract

**Background:**

Trends in cancer survival are a key indicator of progress in the effectiveness of the health system in managing cancer. We aimed to provide long-term trends in a one-number index of survival for all cancers combined, to support national cancer policy.

**Methods:**

We examined long-term trends in a one-number index of net survival (Cancer Survival Index) for all cancers combined in adults in England and Wales. Net survival includes all cancer patients, regardless of whether cancer was the cause of their death. We estimated net survival up to 10 years after diagnosis for 10,769,854 adults diagnosed with a first, primary, invasive cancer during 1971–2018 and followed up to 2019, using anonymised individual records from the National Disease Registration Service for England and the Welsh Cancer Intelligence and Surveillance Unit. We examined trends in the Cancer Survival Index (CSI) at one, five, seven and 10 years after diagnosis, using the entire data set. We present results for selected periods: 1971–72, 1980–81, 1990–91, 2000–01, 2005–06, 2010–11 and 2018.

**Findings:**

During the 48 years 1971–2018, the CSI for England and Wales rose substantially, at all intervals up to 10 years after diagnosis. For patients diagnosed in 1971–72, the CSI was 46.5% at 1 year after diagnosis. For patients diagnosed in 2018, the index is 49.8% at 10 years after diagnosis. The CSI has remained about 10% higher for women than men since the early 1970s. The speed of improvement has slowed down: between 2000–01 and 2005–06, the 10-year CSI rose by 4.0%. Ten years later, the increase between 2010–11 and 2015–16 was only 1.4%.

**Interpretation:**

The slow-down since 2010 of long-term trends in the CSI for all cancers combined in England and Wales is likely to be at least partly explained by longer waits for diagnosis and treatment. A long-term national cancer plan to bring cancer survival trends back towards the best in the world is essential.

**Funding:**

Cancer Research UK.

**Research in context**

**Evidence before this study**

The survival of all patients after a cancer diagnosis is a key measure of the effectiveness of a national health system. The fourth national cancer strategy for England (2015) set an ambitious target for 10-year net survival to reach 75% by 2034 for all cancers combined.

The Cancer Survival Index (CSI) is a standardised, one-number summary of net survival for all cancers combined. It captures trends over time and differences in survival by age, sex and type of cancer, and in the risk of death from other causes. It is designed to help monitor long-term progress in the effectiveness of the healthcare system for managing all cancers.

The 10-year CSI for England and Wales improved by 24% over the 40 years to 2010–11.

**Added value of this study**

We updated trends in the Cancer Survival Index to include all adults diagnosed with a cancer during the 48 years 1971–2018, and followed up to 2019.

For patients diagnosed in 2018, the Cancer Survival Index (CSI) for all cancers combined at 10 years after diagnosis (49.8%) is now higher than the CSI at one year for those diagnosed during 1971–72 (46.5%), a remarkable improvement. The CSI for women has remained about 10% higher than for men since the early 1970s, at all intervals up to 10 years after diagnosis.

However, the speed of improvement in survival has slowed down. In the five years between 2000–01 and 2005–06, the 10-year CSI rose by 4.0%. Ten years later, the increase between 2010–11 and 2015–16 was only 1.4%. The slow-down of progress in long-term survival is seen for many individual cancers, implying a system-wide challenge.

**Implications of all the available evidence**

The slow-down of long-term trends in cancer survival in England and Wales since 2010-11 should drive a long-term National Cancer Plan to accelerate further improvement. The Department of Health and Social Care has recently recognised the need to “bring this country's cancer survival rates back up to the standards of the best in the world.”

Trends in the Cancer Survival Index provide a real-world baseline from which to develop and monitor targets for the National Cancer Plan.

The cancer registries in the UK are crucial for informing cancer control priorities, and for long-term monitoring of their impact. Without their collection and expert curation of detailed data, over many decades, the type of analysis reported here would be impossible.

## Introduction

The survival of all patients following a cancer diagnosis is a key measure of the overall effectiveness of a national health system in dealing with cancer.^1^ National cancer strategies in England and Wales since the year 2000 have all been underpinned by trends in five-year cancer survival, and motivated by differences in survival trends between the UK and other European countries.^2^, ^3^, ^4^, ^5^

From the 1970s to 2010, survival in England and Wales improved dramatically for some cancers, but much less so for others. Five-year survival differs widely between the various types of cancer, from above 90% for testicular cancer and melanoma of the skin to below 25% for cancers of the oesophagus, pancreas, lung and brain.^6^^,^^7^ For many cancers, survival also differs between men and women.

The national cancer strategy for England, published in 2015, set out the ambition for 10-year net survival for all cancers combined to reach 75% by 2034.^8^ This would imply that, on average, and for all cancers combined, three in four people diagnosed with cancer in 2034 could be expected to have the same chances of surviving at least 10 years after their cancer diagnosis as people who have not been diagnosed with cancer.

A cancer survival index enables us to monitor progress towards this goal to improve the overall effectiveness of the healthcare system for cancer. It is a summary index that combines the widely differing patterns of survival for each type of cancer, in each sex and age group, in a single number that is comparable over time, i.e., a “one-number index” of survival for all cancers combined.

An index of one-year net survival has been published for adults in England.^9^ It combined survival estimates for breast, colorectal and lung cancers separately, and all other cancers combined, but excluding prostate cancer. This index rose from 62.0% for patients diagnosed in 2001 to 72.8% for 2016.

We have published long-term trends in an index of net survival up to 10 years after diagnosis, including all cancers, for adults diagnosed in England and Wales between 1971 and 2011.^10^ This highlighted substantial increases in both short-term and long-term net survival up to 10 years in both England and Wales. Here, we set out to update trends in this index to include all adults diagnosed with a cancer during the 48 years 1971–2018.

## Methods

The national cancer registries in England and Wales have been operational since 1971, with high levels of completeness.^11^^,^^12^ Data on race and ethnicity were not available. We obtained anonymised individual records for all adults (15–99 years) diagnosed with a cancer in England and Wales during 1971–2018 and followed up for their vital status to 31 December 2019. The data were originally coded in the eighth revision of the International Classification of Diseases (ICD-8),^13^ ICD-9,^14^ or ICD-10,^15^ later in the International Classification of Diseases for Oncology (ICD-O)^16^ or ICD-O-3.^17^ Data in earlier classifications had been trans-coded with standard conversions in each registry.

The data items in each tumour record were checked for logical coherence against 20 sets of criteria: details of the approach have been published.^6^^,^^18^ The criteria included definite errors (e.g., sex-site errors, invalid dates, impossible date sequence or missing vital status) and possible errors, including a wide range of inconsistencies between age, tumour site and morphology. We excluded records with missing data on sex or vital status, or incomplete dates of birth, diagnosis or last known vital status. We also excluded tumours that were *in situ*, of uncertain behaviour, metastatic from another organ, or unknown whether primary or metastatic. Registrations made solely from a death certificate or following autopsy were also excluded, because the date of diagnosis, and thus the duration of survival, were unknown. We included only the first, primary, invasive malignancy in each patient in survival analyses, although benign tumours of the brain and central nervous system were included.

We estimated net survival up to 10 years after diagnosis by sex and age for each of 21 cancers or groups of cancers (bladder, brain, breast (women), cervix, colon, Hodgkin lymphoma, kidney, larynx (men), leukaemia, lung, melanoma of the skin, myeloma, non-Hodgkin lymphoma, oesophagus, ovary, pancreas, prostate, rectum, stomach, testis and uterus), and for all other cancers combined. Cancers of the breast in men and of the larynx in women were included among all other cancers. Basal cell carcinomas and squamous cell carcinomas of the skin were not included.

Net survival is the probability that cancer patients survive their cancer up to a given time since diagnosis, such as five years, after controlling for the risk of death from other causes (background mortality), which is higher in the elderly.^6^ We used life tables of all-cause mortality by single year of age, sex, Government Office Region, deprivation quintile and single calendar year (1971–2019), to correct for differences and changes over time in background mortality by age, sex, region of residence and socio-economic deprivation, separately for England and Wales.^19^, ^20^, ^21^

Geographical variation in background mortality is strongly influenced by socio-economic deprivation.^22^, ^23^, ^24^, ^25^ The metric of socio-economic deprivation was derived from quintiles of the Indices of Multiple Deprivation (IMD) from 2001.^26^ For earlier years, deprivation quintiles were based on the Carstairs Index.^27^ The life tables were provided by the Office for National Statistics (ONS). This was done to take account of the effect on net survival, to the extent possible, of the gradient in background mortality between quintiles of socio-economic status,^28^ regardless of the socio-economic metric that was available at the time.

We used flexible parametric models to estimate the excess hazard of death^29^^,^^30^ for each combination of cancer, sex and age group (15–44, 45–54, 55–64, 65–74 and 75–99 years), using all data for the 48-year period 1971–2018. We used the Stata^31^ command *stpm2*^32^ to fit eight candidate models for each of 37 combinations of cancer and sex (see Web-Appendix). In each model, age and year of diagnosis were included as main effects, either as linear variables, or modelled on a continuous scale with regression cubic splines, to assess potential non-linearity of the excess hazards with respect to age or year of diagnosis. An interaction term between age and year of diagnosis was also included, to allow for different age effects for each year of diagnosis. More detail is provided in the Web-Appendix.

The best-fitting model for each combination of cancer and sex, chosen as the one with the smallest Akaike Information Criterion (AIC),^33^ was selected to estimate cancer survival from the available data. For some combinations of cancer and sex, some of the eight candidate models did not reach convergence. If four or more models did not converge, we ran a simpler alternative model including only age at diagnosis as the main effect, and estimated its goodness of fit with the AIC. This modelling approach allows estimation of net survival at individual level. Net survival is then estimated in each age group with the Stata “predict” command, as the mean of the individual net survival estimates produced by the model for all patients belonging to that age group. We predicted net survival at one, five, seven and 10 years after diagnosis from the best-fitting model, for each patient included in the analysis, from their cumulative excess hazard.^34^^,^^35^

The Cancer Survival Index for England and Wales was constructed as a weighted average of the net survival estimates for every combination of age at diagnosis (five age groups) for 18 cancers in women and 17 cancers in men, plus all other cancers combined in each sex. The weights were the proportions of patients diagnosed in England and Wales during 1996–99 in each of the 185 combinations of cancer-sex (37) and age group (5). These are the weights used in previous analyses,^10^ to ensure consistency.

We examined 48-year trends in the Cancer Survival Index for men and women combined, and for each sex separately in England and Wales, up to 10 years after diagnosis. We present survival estimates for patients diagnosed during eight selected calendar periods: 1971–72, 1980–81, 1990–91, 2000–01, 2005–06, 2010–11, 2015–16 and 2018. These periods were selected to simplify presentation of long-term trends, but all the data over 48 years were used in constructing the models.

### Role of the funding source

The funding source played no part in the design, data collection, quality control, analysis, interpretation of the findings, writing of the manuscript, or the decision to submit for publication. The corresponding author had full access to all data and responsibility for submission for publication.

### Approvals

We maintained annual approvals for processing sensitive personal data for this research from the UK's statutory Health Research Authority (reference 19/CAG/0035), the NHS Research Ethics Service (19/LO/0426), the Ethics Committee of the London School of Hygiene and Tropical Medicine (#16332), NHS England (DARS-NIC-656861-S5H3R-v0.2) and Public Health Wales.

## Results

We received data on 11,707,005 adults who were registered with a malignancy in England and Wales between 1971 and 2018 and followed up for their vital status until 31 December 2019. We excluded 592,446 records (5.1%), mainly as benign or *in situ* tumours. The other exclusions were for incomplete dates of birth, diagnosis or last known vital status.

Among the 11,114,559 eligible records, we excluded a further 344,705 (3.1%) because the registration was made only from a death certificate, or because the vital status or sex was unknown, or the sequence of dates was invalid, or there was an inconsistency of sex-site, site-morphology, age-site, age-morphology or age-site-morphology. We included the data for 10,769,854 adults (96.9% of those eligible) with a first, primary, invasive malignancy, as well as benign tumours of the brain and central nervous system. Of these patients, 10,098,857 (93.8%) were resident in England and 670,997 (6.2%) in Wales. Table 1 shows the number of adults diagnosed during the eight periods for which separate survival estimates are shown, by age and sex. All 48 years of data were included in the models.

**Table 1:** Number of patients diagnosed in England and Wales in selected calendar periods, by age group and sex.

Age group (years)	1971–1972		1980–1981		1990–1991		2000–2001	
	Men	Women	Men	Women	Men	Women	Men	Women
15–44	6402	10,267	7794	13,376	9734	16,715	11,668	20,048
45–54	12,139	17,675	12,956	18,482	12,659	20,997	17,005	30,230
55–64	32,864	27,362	36,537	33,359	33,215	36,198	41,753	41,955
65–74	44,315	32,597	61,401	45,244	59,975	46,996	73,700	51,496
75–99	26,768	30,858	45,731	48,649	60,740	59,326	83,053	77,341
All ages	122,488	118,759	164,419	159,110	176,323	180,232	227,179	221,070

Age group (years)	2005–2006		2010–2011		2015–2016		2018	
	Men	Women	Men	Women	Men	Women	Men	Women
15–44	12,765	22,265	13,642	23,997	13,973	25,104	6997	12,454
45–54	17,216	29,630	20,578	35,473	23,297	39,098	12,241	19,562
55–64	50,724	49,091	58,044	54,271	57,665	54,322	32,222	28,462
65–74	78,281	56,470	91,582	64,258	106,273	76,646	58,392	39,766
75–99	91,906	83,698	105,127	92,416	118,667	100,609	60,853	50,727
All ages	250,892	241,154	288,973	270,415	319,875	295,779	170,705	150,971

**Fig. 1:**
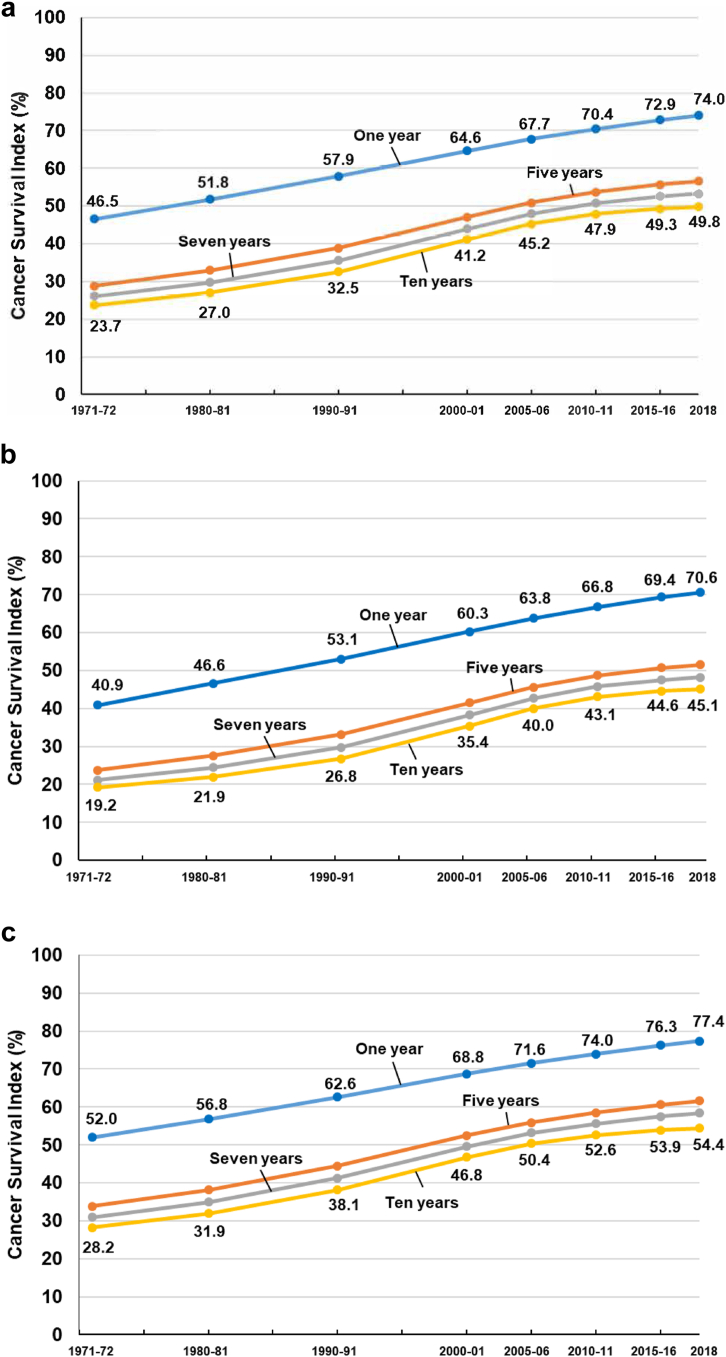
Trends in the index of net survival (%) at 1, 5, 7 and 10 years, for all cancers combined: England and Wales, selected periods during 1971–2018. a) men and women combined, b) men, and c) women.

The Cancer Survival Index for England and Wales for men and women combined has increased substantially between 1971 and 2018, at all intervals up to 10 years since diagnosis (Table 2, Fig. 1a). For example, for patients diagnosed during 1971–72, the survival index at one year after diagnosis was 46.5% (95% confidence interval 46.4–46.6%). For patients diagnosed in 2018, the survival index at ten years after diagnosis had reached 49.8% (49.7–49.9%). The 5-year survival index rose from 28.8% (28.6–29.1%) in 1971–72 to 56.6% (56.6–56.7%) for 2018.

**Table 2:** Cancer **S**urvival **I**ndex (CSI): 48-year trends in net survival (%) with 95% confidence interval (CI) at one, five and 10 years after diagnosis, all cancers combined, for eight selected calendar periods of diagnosis, by sex: adults (15–99 years) in England and Wales.

	One year		Five years		10 years	
	CSI (%)	95% CI	CSI (%)	95% CI	CSI (%)	95% CI
**1971–1972**						
**All adults**	**46.5**	46.4–46.6	**28.8**	28.6–29.1	**23.7**	23.4–24.1
**Men**	**40.9**	40.7–41.1	**23.7**	23.0–24.4	**19.2**	18.2–20.2
**Women**	**52.0**	51.9–52.2	**33.8**	33.4–34.2	**28.2**	27.6–28.8
**1980–1981**						
**All adults**	**51.8**	51.7–51.8	**32.9**	32.8–33.0	**27.0**	26.8–27.2
**Men**	**46.6**	46.5–46.7	**27.6**	27.3–27.9	**21.9**	21.4–22.4
**Women**	**56.8**	56.7–56.8	**38.1**	37.9–38.3	**31.9**	31.6–32.2
**1990–1991**						
**All adults**	**57.9**	57.9–57.9	**38.9**	38.8–39.0	**32.5**	32.4–32.6
**Men**	**53.1**	53.0–53.1	**33.2**	33.0–33.4	**26.8**	26.5–27.2
**Women**	**62.6**	62.6–62.6	**44.5**	44.4–44.6	**38.1**	37.9–38.3
**2000–2001**						
**All adults**	**64.6**	64.6–64.6	**47.0**	47.0–47.1	**41.2**	41.1–41.2
**Men**	**60.3**	60.3–60.3	**41.5**	41.3–41.6	**35.4**	35.2–35.6
**Women**	**68.8**	68.8–68.8	**52.5**	52.4–52.6	**46.8**	46.7–46.9
**2005–2006**						
**All adults**	**67.7**	67.7–67.7	**50.9**	50.8–50.9	**45.2**	45.2–45.3
**Men**	**63.8**	63.8–63.8	**45.7**	45.6–45.8	**40.0**	39.8–40.2
**Women**	**71.6**	71.6–71.6	**56.0**	55.9–56.0	**50.4**	50.3–50.5
**2010–2011**						
**All adults**	**70.4**	70.4–70.4	**53.7**	53.6–53.7	**47.9**	47.8–47.9
**Men**	**66.8**	66.8–66.8	**48.7**	48.6–48.8	**43.1**	42.9–43.3
**Women**	**74.0**	74.0–74.0	**58.5**	58.5–58.5	**52.6**	52.5–52.7
**2015–2016**						
**All adults**	**72.9**	72.9–72.9	**55.7**	55.7–55.8	**49.3**	49.2–49.4
**Men**	**69.4**	69.4–69.4	**50.7**	50.6–50.8	**44.6**	44.4–44.9
**Women**	**76.3**	76.3–76.3	**60.6**	60.6–60.7	**53.9**	53.8–54.1
**2018**						
**All adults**	**74.0**	74.0–74.0	**56.6**	56.6–56.7	**49.8**	49.7–49.9
**Men**	**70.6**	70.6–70.6	**51.5**	51.4–51.6	**45.1**	44.8–45.4
**Women**	**77.4**	77.4–77.4	**61.7**	61.6–61.7	**54.4**	54.3–54.6

The 10-year survival index has increased more slowly since 2010 than over the preceding 20 years (Fig. 1a). It improved by 4.0% over the five years between 2000–01 and 2005–06 (from 41.2% to 45.2%). Ten years later, it had risen by only 1.4% over the five years between 2010–11 and 2015–16 (from 47.9% to 49.3%) (Table 2, Fig. 1a).

For men, the all-cancers survival index has improved markedly since 1971–72, at all intervals up to 10 years after diagnosis (Table 2, Fig. 1b). At one year after diagnosis, the index was 40.9% (40.7–41.1%) for 1971–72, rising to 70.6% (70.6–70.6%) for 2018, while the 10-year survival index rose from 19.2% (18.2–20.2%) to 45.1% (44.8–45.4%) over the same period.

For women, the all-cancers survival index has also risen substantially (Table 2, Fig. 1c). The one-year survival index rose from 52.0% (51.9–52.2%) for 1971–72 to 77.4% (77.4–77.4%) in 2018. The 10-year survival index rose from 28.2% (27.6–28.8%) to 54.4% (54.3–54.6%) over the same period.

### Ten-year survival for selected cancers

Ten-year survival has increased steadily since the 1970s for patients diagnosed with a cancer of the cervix, kidney, colon or rectum, or with melanoma of the skin, or leukaemia or non-Hodgkin lymphoma (Tables 7–10, Web-Appendix pages 29–60).

**Fig. 2:**
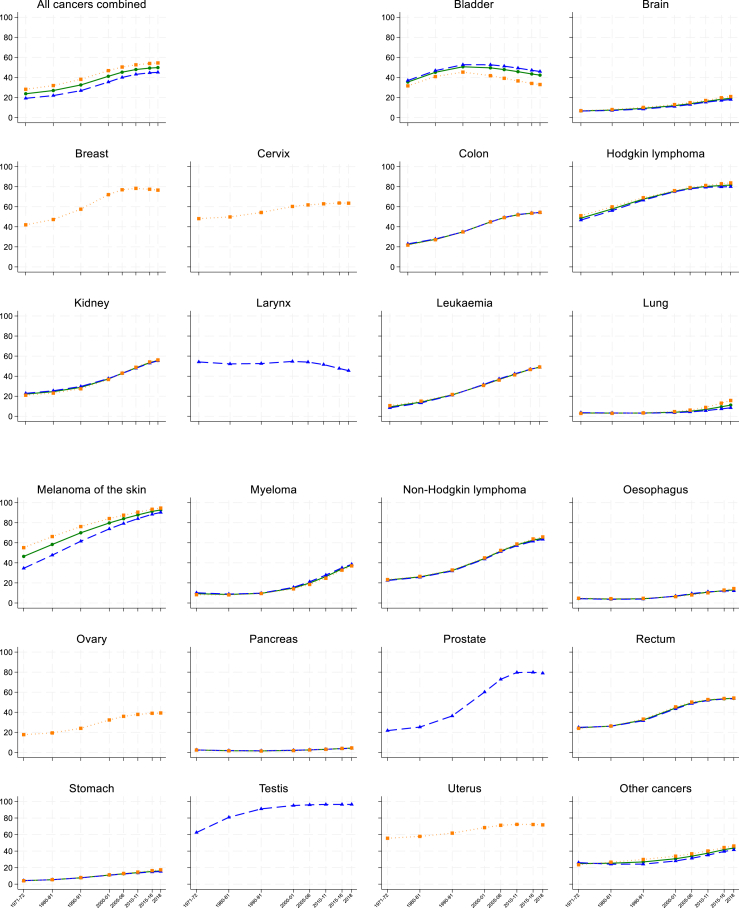
Trends in age-standardised 10-year net survival (%), for 22 cancers and all cancer combined, for men (blue line), women (orange line) and both sexes combined (green line), in England and Wales, for selected periods during 1971–2018.

For patients diagnosed in 2018, the age-standardised 10-year net survival estimate was above 90% for testicular cancer and melanoma of the skin, and in the range 70–89% for Hodgkin lymphoma, and cancers of the prostate, breast (women) and uterus. Ten-year survival was in the range 50–69% for cancers of the cervix, colon, kidney and rectum, and non-Hodgkin lymphoma, and below 50% for all other cancers (Fig. 2; Web-Appendix).

For testicular and uterine cancers, the speed of improvement in survival has slowed down since 2005–06. For cancers of the breast and prostate, the deceleration is more recent, since 2010–11 (Web-Appendix). Ten-year survival has fallen by 7–8% for both men and women with cancer of the bladder between 2000–01 (52.6% and 41.8%, respectively) and 2018 (45.9% and 33.0%). Ten-year survival for men diagnosed with laryngeal cancer has fallen by a similar amount between 2005–06 and 2018, from 54.1% to 45.5%.

Ten-year net survival was below 40% for patients diagnosed in 2018 with malignant myeloma or cancer of the lung, oesophagus, ovary, pancreas, stomach or brain, but with the exception of pancreatic cancer, substantial improvements have nevertheless occurred since the 1970s (Fig. 2; Web-Appendix).

For most cancers, 10-year survival for women diagnosed in 2018 was around 3–5% higher than for men (Table 2). For bladder cancer, however, 10-year survival was 13% higher for men. The difference between men and women was less than 1% for cancers of the colon, kidney, pancreas and rectum, and for leukaemia.

The estimates of survival for each cancer at one, five, seven and 10 years after diagnosis, which are the components of the survival index, are provided by age, sex and selected calendar period in the Web-Appendix.

## Discussion

For many cancers, substantial improvements in net survival have been seen since the early 1970s. The chances of surviving up to 10 years after a cancer diagnosis vary widely between the different types of cancer, even after correcting for other causes of death (net survival). For most cancers, net survival also varies between age groups, and between men and women. The age profile of people who are diagnosed with cancer differs widely between the cancers, and it can change over time.

The Cancer Survival Index for England and Wales up to 10 years after diagnosis improved substantially between 1971–72 and 2018. The 10-year survival index for patients diagnosed in 2018 is now higher than the one-year survival index for those diagnosed during 1971–72, a remarkable improvement. The index has remained about 10% higher for women than men since the early 1970s, at all intervals up to 10 years after diagnosis. This is partly because, for most cancers, survival for women is higher than for men, but it is also partly due to high survival for women with breast cancer, which carries a large weight in the survival index.

However, the speed of improvement in survival up to 10 years after diagnosis from all cancers combined has slowed down since 2010–11. The 10-year cancer survival index for patients diagnosed in 2018 is 49.8%, the same as reported in the previous analysis^10^ for 2010–11. Our estimate for 2010–2011, using data for an additional 3 million cancer patients, is slightly lower, at 47.9%, but it is clear that the 10-year cancer survival index has improved very little since 2010–11.

The numbers of patients diagnosed with cancer in England and Wales increased substantially between 1971 and 2018, particularly among those aged 75 or over, for whom comorbidities may sometimes be considered to preclude the full range of treatment options. This places additional demand on health services that have not always seen a commensurate increase in resources.

The deceleration in survival improvement since 2010-11 seems unlikely to be attributable to the data or the analytic methods, which are closely similar to those used in 2015. We used the same analytic approach to estimate net survival, and the same weights to produce the all-cancers survival index. At one year after diagnosis, the survival index for 2010–11 was 70.5% in the previous report, almost identical to the value of 70.4% obtained in this analysis. The five-year index for 2010–11 in that report was 54.3%, also similar to our estimate at 53.7%. The 10-year survival index for 2010–11 was estimated at 49.8% in the previous report, but with a further eight years of follow-up, we estimated the 10-year survival index for 2010–11 at 47.9%. This decline is not large, but if survival had been improving steadily, we would have expected to see the 10-year index for 2010–11 increase with eight additional years of follow-up.

The differences between previous and current estimates of the Cancer Survival Index up to 10 years for patients diagnosed during 2010–11 are progressively larger for longer intervals since diagnosis: −0.1% at one year, −0.6% at five years and −1.9% at 10 years. These differences reflect slower improvement in longer-term survival between 2010–11 and 2018 than over the 40 years between 1971–72 and 2010–11 (Fig. 1).

With progressive increases in longer-term survival, a higher proportion of cancer survivors now die from a cause of death that is unrelated to their cancer. The death certificate of those persons will not include cancer as the underlying cause of death. For that reason, cancer mortality rates are progressively less informative about cancer outcomes. Population-based survival estimates are the key outcome metric, because they include everyone who has been diagnosed with cancer.^36^ As in the general population, the risk of death among cancer survivors from causes other than the cancer also increases with age. It also differs between men and women, and it changes over time. Net survival estimates are corrected for these differences in background mortality by age and sex, and over time. In this analysis, the net survival estimates were also corrected for differences and trends in background mortality between geographic regions and by socio-economic status. They reflect the chances of surviving after the cancer diagnosis, regardless of the cause of death.

Summarising overall progress in population-based cancer survival over a 48-year period with a single number requires a measure that captures all these aspects of cancer survival, and is comparable over time. An index of net survival for all cancers combined does this. It is a weighted average of the estimates of net survival for every combination of age, sex, and type of cancer. To achieve comparability over time, the weights used to produce the index must be the same for all calendar periods. We used the same weights as in previous analyses, derived from the data for all cancers diagnosed in England and Wales during the period 1996–99.

The merit of an index of net survival that is jointly standardised by age, sex and the distribution of the different types of cancer is that it will only change if net survival for one or more cancers has changed over time, for any age group, or either sex. A survival index is not affected by changes in the frequency of any cancer in men or women, or the age profile of cancer patients, such as the recent increase in cancer risk in young adults in the UK and other countries.^37^, ^38^, ^39^

A cancer survival index has been used to compare survival from all cancers combined as a comparative index of cancer control in European countries contributing to the EUROCARE programme.^40^ A similar index of five-year survival in the USA, based on data from 31 states for patients diagnosed during 2006–12, has been proposed as a baseline measure for monitoring progress in cancer control, from “improving early cancer diagnosis and access to timely, evidence-based treatment.”^41^ The index was 8% higher for whites than blacks.

It is important to note that an all-cancers survival index does not have a clinical interpretation, such as the chances of survival for any individual patient with a cancer. It is a one-number summary of survival from all cancers in a given country, corrected for differences in the chances of dying from other causes, by age and type of cancer, between men and women, and over time. In this sense, it provides a consistent assessment of long-term progress in the overall effectiveness of the healthcare system for all cancer patients in that country. The index is derived from age-sex-cancer weights in the country where it is deployed, so the values of the index in one country cannot be directly compared with those in another country, although the trends may be similar.

The main strength of this study is the use of data from high-quality, population-based, national cancer registries in England and Wales to summarise trends in a one-number index of net survival from all cancers combined in adults over 48 years. Trends in this index provide a quantitative insight into the overall effectiveness of the health service in managing cancer. Net survival is corrected for changes in the risk of death from other causes, which are higher in older patients. The index is a weighted average of the survival estimates by age and sex for each cancer, using the same methodology and weights as those used in 2015, and the results are consistent with the estimates for 2010–11, made in 2015, when those data were the most recent available.^10^ Data quality was high: only 3% of 11 million records of invasive primary malignancy were excluded during quality control.

The data cover the period 1971–2018, with follow-up for vital status to 31 December 2019. A key limitation is that more timely access to data would have been preferable, although more recent survival estimates for all cancers combined are not currently available. The cancer registry for England has been disrupted over the last few years, being moved from Public Health England, first to NHS Digital, then finally to NHS England, further complicating the lengthy application process for data. Further, data for stage at diagnosis and treatment were not available to help interpret the deceleration in cancer survival trends since 2010.

### Survival trends for selected cancers

For most cancers, 10-year survival in England and Wales has improved steadily since the 1970s, although for some cancers, by very little. As a result, 10-year survival now differs even more widely between the various types of cancer than in the past. For patients diagnosed in 2018, the 10-year net survival estimate for pancreatic cancer is 4.3%, with little change since 1971–72, reflecting the difficulty in detecting the disease at an early stage.^42^^,^^43^ Improvements in 10-year net survival have also been very small for cancers of the oesophagus, stomach, lung and brain. For testicular cancer, however, 10-year survival has reached 96.5%.

The improvement in survival for breast cancer may be partially attributable to earlier detection, often enhanced by the mass screening programme. The NHS Breast Screening Programme began in 1988 in England^44^ and 1989 in Wales,^45^ for women aged 50–64 years. Ten-year survival improved from 42.0% for women diagnosed during 1971–72 to 47.3% for women diagnosed during 1980–81, but much larger increases were seen over the next two decades, to 57.6% for 1990–91 and 72.0% for 2000–01. Later increases have been much smaller, reaching 76.6% for women diagnosed in 2018. The smaller additional gains in breast cancer survival over the last 10–15 years may reflect the consistently high uptake of screening programmes that have been available since the 1980s and early 1990s. Invitation of women aged 40–49 years for breast screening may affect survival in the future.^46^

Cervical cancer screening began in England in 1964, but the programme was substantially revised in the 1990s.^47^ Ten-year survival rose from 48.2% for women diagnosed during 1971–72 to 62.9% in 2010–11, but it has risen very little since then, to 63.5% for 2018. Incidence rates of invasive cancer of the cervix have fallen substantially with more efficient screening, and most cervical cancers are detected and treated while *in situ*.^48^

The largest improvements in survival from cancers of the large bowel (colon and rectum) are attributable to diagnosis by colonoscopy, advances in surgical technique,^49^^,^^50^ and chemotherapy.^51^ The surgical advances predate screening, which started for 60-69 year-olds in 2006 in England,^52^ and 2008 in Wales.^53^ For men and women combined, 10-year survival from colon cancer rose from 22.4% for 1971–72 to 52.1% for 2010–11, but it has increased little since then, to 54.3% for 2018. The trend for rectal cancer is similar. The uptake of bowel screening with faecal occult blood tests (FOBT) has been low,^54^ but the introduction of faecal immunochemical tests (FIT) since 2019,^55^ and the extension of screening to 50–59 year-olds from 2024,^56^ may lead to earlier diagnosis.

For cancers of the breast, cervix, rectum, prostate, testis and uterus, 10-year survival appears to have reached a plateau over the last 10–15 years, while survival for cancers of the urinary bladder and larynx has been decreasing. The increase in prostate cancer survival from the 1970s accelerated between the early 1990s and 2010 (Web-Appendix Fig. 2), which probably reflects increased use of prostate-specific antigen (PSA) testing, with an increase in diagnosis of less aggressive tumours. The declining trend in bladder cancer survival was seen in the previous analysis.^10^ This is probably due to the reclassification of certain types of bladder cancer that have a very good prognosis as non-invasive, rather than any decline in the efficacy of treatment.^57^^,^^58^ Survival estimates that only include invasive cancers will be lower after removal of a sub-group of cancers with very high survival that are no longer considered as invasive.

The focus on improving cancer outcomes in the UK has been on early detection and diagnosis and access to better treatment, with more effective drugs.^59^ The proportion of cancers in England diagnosed at an early stage (stage 1 or 2) has remained fairly constant at around 54–56% since national data became available from 2013,^60^ despite the target that was set by NHS England to reach 75% by 2028.^61^ This may reflect falling performance against cancer waiting times standards.^60^^,^^62^ The proportion of patients who obtained treatment within the specified Cancer Waiting Times has fallen considerably since 2013. The number of people in England being referred urgently by a GP with a suspicion of cancer has risen over the last decade, leading to more demand from diagnostic services.

The fourth national cancer strategy for England, set in 2015, was motivated by the persistent survival deficit between England and comparable European countries.^63^ The survival index for all cancers combined has not improved much since 2015, but plans for a fifth National Cancer Plan were withdrawn in January 2023, leaving England as one of the few high-income countries “in which a national cancer plan was not a central pillar of national health policy.”^64^ It was proposed instead to subsume cancer into a “major conditions strategy,” covering a wide range of other diseases, including cardiovascular disease, stroke, musculoskeletal disorders, diabetes, mental health and respiratory disease.^65^ It is at least questionable whether a multi-disease strategy could be more effective for cancer than a dedicated national cancer control plan,^66^ as recommended by WHO.^67^ In calling for evidence and proposals for a new National Cancer Plan for England in February 2025, the Department of Health and Social Care has now recognised the need to “bring this country's cancer survival rates back up to the standards of the best in the world.”^68^

A similar question may be raised about the National Disease Registration Service, into which the National Cancer Registry for England has been absorbed. A national cancer registry is maintained in Scotland, Wales and Northern Ireland.

Timely monitoring of progress in cancer control in England will require long-term support for a dedicated National Cancer Registry for England, with a clear identity and sufficient expert staff, since: “cancer registries are the bedrock of cancer care.”^69^ High-quality patient-level data, securely linked to other health datasets, should form part of the “critical national infrastructure”^70^ to help drive improvements in cancer care. With appropriate governance, more timely access to cancer data for service evaluation and research would ensure that their value to the population can be delivered more promptly. At present, data access lags behind real time by a year, often more.^70^

National improvements in cancer survival will require sustained improvements in early presentation, screening and early diagnosis,^42^ and in prompt access to thorough investigation and optimal treatment. Longer waiting times for diagnosis and the abandonment of key targets for rapid access to treatment, coupled with the prolonged slow-down in cancer survival trends, reflect a National Health Service that is in great difficulty. A new, long-term National Cancer Plan is required.^71^

### Conclusion

Progress towards the goal for 2034 of 75% 10-year net survival for all cancers combined has slowed down over the last 10–15 years. The 10-year cancer survival index rose by 24% over the 40 years to 2010–11, reaching 47.9%. By 2018, it had risen to 49.8%, a further increase of only 1.9% in eight years. The 10-year survival index would need to increase much more quickly, by an average of 1.6% every year over 16 years (2018–2034), to reach the target of 75% by 2034.

The slow-down of long-term trends in survival in England and Wales since 2010–11 should drive urgent national planning to accelerate further improvement in cancer survival.

## Contributors

MPC, CA, MM and VDC drafted the protocol. MPC, PM, MM, CA and JS obtained statutory and ethical approvals and contributed to data acquisition. VDC, DH and SS prepared the life tables. MM, PM, VDC, CA and MPC had access to all the raw data, and did the data preparation, quality control, and analyses. All authors checked the results. MPC, CA, MM, PM, and VDC drafted the report. All authors had access to the results of quality control and analysis, and contributed to interpretation of the findings. All authors contributed to writing the final report and approved the version to be published.

## Data sharing statement

The data we received for this study from the cancer registries of England and Wales are sensitive personal data relating to human subjects, many of them still alive. We cannot share the raw data, which we hold in trust. Our contracts with the registries do not allow sharing of the data with any external repository or data processor.

## Declaration of interests

We declare no competing interests.

## Appendix A Supplementary data

Web-appendix
