# Supplementary material for: Trends over 48 years in a one-number index of survival for all cancers combined, England and Wales (1971–2018): a population-based registry study
Source: Lancet Reg Health Eur. 2025 Aug 13;56:101385. doi: 10.1016/j.lanepe.2025.101385 (PMC12859600; doi:10.1016/j.lanepe.2025.101385)

Appendix

	Page	Content
**Table 1**	**1**	Number of patients by cancer, age group and sex, selected periods during 1971 2018.
**Table 2**	**9**	Standardised net survival (%) at 1, 5, 7 and 10 years for all cancers combined (CSI) and for 22 selected cancers, by sex, selected periods during 1971-2018.
**Table 3**	**25**	Age-sex-cancer weights: to produce the cancer survival index for all adults, all cancers and all ages combined.
**Table 4**	**26**	Age-cancer weights for each sex: to produce a separate cancer survival index for men and women, for all cancers and all ages combined.
**Table 5**	**27**	Age-sex weights for each cancer: to produce age- and sex-standardised survival estimates for each cancer.
**Table 6**	**28**	Age-weights for each cancer and each sex: to produce age-standardised survival estimates for each cancer, by sex.
**Table 7**	**29**	One-year survival by age and sex, for each cancer, selected periods during 1971-2018.
**Table 8**	**37**	Five-year survival by age and sex, for each cancer, selected periods during 1971-2018.
**Table 9**	**45**	Seven-year survival by age and sex, for each cancer, selected periods during 1971-2018.
**Table 10**	**53**	Ten-year survival by age and sex, for each cancer, selected periods during 1971-2018.
**Figure 1**	**61**	Trends in age-standardised one-year net survival (%), for 22 cancers and all cancer combined, by sex and both sexes combined, selected periods during 1971-2018.
**Figure 2**	**63**	Trends in age-standardised five-year net survival (%), for 22 cancers and all cancer combined, by sex and both sexes combined, selected periods during 1971-2018.
**Figure 3**	**65**	Trends in age-standardised seven-year net survival (%), for 22 cancers and all cancer combined, by sex and both sexes combined, selected periods during 1971-2018.

List of the models deployed in the analyses. See Methods section of article for details.

1. Non-linear and non-proportional effects of age and year of diagnosis, and a non-linear and non-proportional interaction between age and year of diagnosis
2. Non-linear and non-proportional effects of age and year of diagnosis, and a non-linear interaction between age and year of diagnosis
3. Non-linear and non-proportional effects of age and year of diagnosis, and a non-proportional interaction between age and year of diagnosis
4. Non-linear effect of age and year of diagnosis and a non-proportional effect of year of diagnosis and a non-linear interaction between age and year of diagnosis
5. Non-linear and non-proportional effects of age and non-linear year of diagnosis
6. Non-linear and non-proportional effects of age and year of diagnosis
7. Non-linear and non-proportional effect of age and linear and proportional effect of year of diagnosis
8. Linear effect of age and year of diagnosis and non-proportional effect of age

Additional notes on methodology – see Methods section of the article

The year of diagnosis was modelled as a restricted cubic spline with 3 degrees of freedom. This choice gave the best balance between flexibility and overfitting, because it placed 2 knots at equal time intervals. Survival trends are usually fairly smooth, and three degrees of freedom were sufficient to capture this.

For age at diagnosis, we selected knots at a specific age, rather than specifying the degrees of freedom. We aimed to take account of the very different age distributions of patients with each type of cancer, and of the non-linear effects of age at diagnosis on survival. Survival is usually lower for patients who are older at diagnosis, but the slope of the decline in net survival with age at diagnosis varies with the lethality of the cancer.

The following knots were selected:

- 4 knots at 15, 25, 65 and 99 years for Hodgkin lymphoma
- 4 knots at 15, 35, 65 and 99 years for cervical cancer and melanoma
- 4 knots at 15, 40, 65 and 99 years for brain tumours and ovarian cancer
- 4 knots at 15, 50, 70 and 99 years for non-Hodgkin lymphoma and cancers of the breast, colon and uterus
- 3 knots at 15, 65 and 99 years for all other cancers

This modelling approach allows estimation of net survival at individual level. Net survival is then estimated (or "predicted" by the model in the interval covered by the data) in each age group, as the mean of all the individual net survival estimates produced by the model for all patients belonging to that age group.

Appendix Table 1. Number of patients by cancer, age group and sex: diagnosed in England and Wales during 1971-72

Number of patients

Age group (years)	Bladder Men	Bladder Women	Brain Men	Brain Women	Breast Men	Breast Women	Cervix Men	Cervix Women	Colon Men	Colon Women
**15-44**	302	64	456	309	-	3,917	-	1,273	337	399
**45-54**	838	266	471	277	-	6,508	-	1,820	771	970
**55-64**	2,278	619	657	471	-	7,706	-	1,609	1,985	2,149
**65-74**	3,377	1,057	366	288	-	7,164	-	1,109	2,852	3,531
**75-99**	2,132	1,152	54	76	-	5,766	-	706	2,334	4,626
	8,927	3,158	2,004	1,421	-	31,061	-	6,517	8,279	11,675

Number of patients

Age group (years)	Hodgkin's lymphoma Men	Hodgkin's lymphoma Women	Kidney Men	Kidney Women	Larynx Men	Larynx Women	Leukaemia Men	Leukaemia Women	Lung Men	Lung Women
**15-44**	658	415	138	93	66	-	449	287	795	336
**45-54**	193	102	318	158	281	-	374	282	4,193	1,368
**55-64**	261	125	674	341	689	-	791	504	13,119	2,912
**65-74**	188	171	712	436	752	-	981	790	16,617	3,252
**75-99**	67	103	305	337	324	-	681	834	6,595	1,878
	1,367	916	2,147	1,365	2,112	-	3,276	2,697	41,319	9,746

Number of patients

Age group (years)	Melanoma Men	Melanoma Women	Myeloma Men	Myeloma Women	Non-Hodgkin lymphoma Men	Non-Hodgkin lymphoma Women	Oesophagus Men	Oesophagus Women	Others Men	Others Women
**15-44**	210	416	33	22	372	238	53	40	1,130	1,118
**45-54**	141	303	120	77	335	221	272	154	1,565	1,502
**55-64**	167	269	346	235	515	447	721	433	3,168	2,843
**65-74**	102	211	343	414	528	525	1,050	721	4,027	3,977
**75-99**	70	175	173	315	268	409	736	921	2,742	4,105
	690	1,374	1,015	1,063	2,018	1,840	2,832	2,269	12,632	13,545

Number of patients

Age group (years)	Ovary Men	Ovary Women	Pancreas Men	Pancreas Women	Prostate Men	Prostate Women	Rectum Men	Rectum Women	Stomach Men	Stomach Women
**15-44**	-	699	96	63	14	-	212	176	269	180
**45-54**	-	1,377	426	256	186	-	641	535	900	441
**55-64**	-	1,829	1,110	680	1,489	-	1,777	1,208	3,046	1,230
**65-74**	-	1,677	1,540	1,341	3,935	-	2,537	1,826	4,366	2,485
**75-99**	-	990	1,036	1,482	4,533	-	1,844	2,211	2,850	3,789
	-	6,572	4,208	3,822	10,157	-	7,011	5,956	11,431	8,125

Number of patients

Age group (years)	Testis Men	Testis Women	Uterus Men	Uterus Women	All cancers Men	All cancers Women
**15-44**	812	-	-	222	6,402	10,267
**45-54**	114	-	-	1,058	12,139	17,675
**55-64**	71	-	-	1,752	32,864	27,362
**65-74**	42	-	-	1,622	44,315	32,597
**75-99**	24	-	-	983	26,768	30,858
	1,063	-	-	5,637	122,488	118,759

Appendix Table 1. Number of patients by cancer, age group and sex: diagnosed in England and Wales during 1980-81

Number of patients

Age group	Bladder		Brain		Breast		Cervix		Colon	
(years)	Men	Women	Men	Women	Men	Women	Men	Women	Men	Women
**15-44**	307	86	651	427	-	4,897	-	2,231	411	411
**45-54**	971	294	512	333	-	7,479	-	1,178	959	990
**55-64**	2,822	916	878	617	-	9,482	-	1,801	2,344	2,426
**65-74**	4,805	1,543	760	628	-	10,550	-	1,442	4,411	4,786
**75-99**	3,925	1,963	194	242	-	9,550	-	877	3,966	7,060
	12,830	4,802	2,995	2,247	-	41,958	-	7,529	12,091	15,673

Number of patients

Age group	Hodgkin's lymphoma		Kidney		Larynx		Leukaemia		Lung	
(years)	Men	Women	Men	Women	Men	Women	Men	Women	Men	Women
**15-44**	733	535	179	113	68	-	504	340	690	365
**45-54**	198	80	458	219	344	-	375	246	3,787	1,588
**55-64**	228	115	935	457	850	-	746	500	13,337	4,571
**65-74**	161	158	1,148	640	977	-	1,290	931	21,559	6,187
**75-99**	87	118	611	592	464	-	1,186	1,367	13,194	4,474
	1,407	1,006	3,331	2,021	2,703	-	4,101	3,384	52,567	17,185

Number of patients

Age group	Melanoma		Myeloma		Non-Hodgkin lymphoma		Oesophagus		Others	
(years)	Men	Women	Men	Women	Men	Women	Men	Women	Men	Women
**15-44**	380	770	50	42	546	326	88	47	1,213	1,278
**45-54**	254	478	190	137	486	318	367	196	1,461	1,174
**55-64**	291	494	452	343	818	641	1,052	538	3,083	2,465
**65-74**	301	528	764	672	1,011	960	1,548	993	4,027	3,844
**75-99**	162	413	567	785	644	930	1,206	1,544	2,834	4,689
	1,388	2,683	2,023	1,979	3,505	3,175	4,261	3,318	12,618	13,450

Number of patients

Age group	Ovary		Pancreas		Prostate		Rectum		Stomach	
(years)	Men	Women	Men	Women	Men	Women	Men	Women	Men	Women
**15-44**	-	810	125	64	23	-	279	188	284	170
**45-54**	-	1,520	437	264	258	-	775	539	955	364
**55-64**	-	2,348	1,332	855	2,140	-	2,139	1,459	2,991	1,111
**65-74**	-	2,272	2,168	1,650	7,012	-	3,798	2,527	5,599	2,766
**75-99**	-	1,703	1,603	2,436	7,926	-	3,005	3,568	4,114	4,685
	-	8,653	5,665	5,269	17,359	-	9,996	8,281	13,943	9,096

Number of patients

Age group	Testis		Uterus		All cancers	
(years)	Men	Women	Men	Women	Men	Women
**15-44**	1,263	-	-	276	7,794	13,376
**45-54**	169	-	-	1,085	12,956	18,482
**55-64**	99	-	-	2,220	36,537	33,359
**65-74**	62	-	-	2,167	61,401	45,244
**75-99**	43	-	-	1,653	45,731	48,649
	1,636	-	-	7,401	164,419	159,110

Appendix Table 1. Number of patients by cancer, age group and sex: diagnosed in England and Wales during 1990-91

Number of patients

Age group	Bladder		Brain		Breast		Cervix		Colon	
(years)	Men	Women	Men	Women	Men	Women	Men	Women	Men	Women
15-44	325	127	714	531	-	6,164	-	2,771	416	401
45-54	915	280	531	364	-	10,146	-	1,143	979	882
55-64	2,938	821	861	601	-	14,211	-	1,115	2,604	2,318
65-74	5,268	1,682	861	596	-	11,524	-	1,157	4,742	4,518
75-99	5,512	2,729	385	439	-	12,057	-	862	5,349	8,181
	14,958	5,639	3,352	2,531	-	54,102	-	7,048	14,090	16,300

Number of patients

Age group	Hodgkin's lymphoma		Kidney		Larynx		Leukaemia		Lung	
(years)	Men	Women	Men	Women	Men	Women	Men	Women	Men	Women
15-44	754	528	279	161	83	-	582	427	546	361
45-54	168	64	537	267	322	-	418	295	2,461	1,221
55-64	135	91	1,110	541	798	-	899	510	8,573	3,981
65-74	116	102	1,447	857	1,082	-	1,531	960	16,456	7,370
75-99	64	83	1,088	877	618	-	1,693	1,687	13,941	6,458
	1,237	868	4,461	2,703	2,903	-	5,123	3,879	41,977	19,391

Number of patients

Age group	Melanoma		Myeloma		Non-Hodgkin lymphoma		Oesophagus		Others	
(years)	Men	Women	Men	Women	Men	Women	Men	Women	Men	Women
15-44	743	1,277	74	43	901	525	97	34	1,736	1,639
45-54	494	658	226	152	744	556	449	160	1,904	1,330
55-64	609	712	522	392	1,239	896	1,075	517	3,473	2,384
65-74	586	770	856	774	1,641	1,434	1,977	1,051	4,791	3,938
75-99	473	886	914	1,071	1,465	1,776	1,697	2,040	4,197	6,005
	2,905	4,303	2,592	2,432	5,990	5,187	5,295	3,802	16,101	15,296

Number of patients

Age group	Ovary		Pancreas		Prostate		Rectum		Stomach	
(years)	Men	Women	Men	Women	Men	Women	Men	Women	Men	Women
15-44	-	951	98	68	25	-	258	226	199	145
45-54	-	1,413	420	254	358	-	841	540	607	244
55-64	-	2,256	987	735	2,910	-	2,285	1,258	2,100	691
65-74	-	2,477	1,696	1,536	9,343	-	3,698	2,334	3,837	1,695
75-99	-	2,100	1,844	2,624	13,831	-	3,475	3,637	4,155	3,818
	-	9,197	5,045	5,217	26,467	-	10,557	7,995	10,898	6,593

Number of patients

Age group	Testis		Uterus		All cancers	
(years)	Men	Women	Men	Women	Men	Women
15-44	1,904	-	-	336	9,734	16,715
45-54	285	-	-	1,028	12,659	20,997
55-64	97	-	-	2,168	33,215	36,198
65-74	47	-	-	2,221	59,975	46,996
75-99	39	-	-	1,996	60,740	59,326
	2,372	-	-	7,749	176,323	180,232

Appendix Table 1. Number of patients by cancer, age group and sex: diagnosed in England and Wales during 2000-01

Number of patients

Age group (years)	Bladder		Brain		Breast		Cervix		Colon	
	Men	Women	Men	Women	Men	Women	Men	Women	Men	Women
15-44	202	100	902	606	-	8,106	-	2,272	442	446
45-54	687	244	703	439	-	15,666	-	884	1,177	1,124
55-64	2,228	652	976	606	-	16,336	-	625	3,222	2,493
65-74	4,432	1,352	1,073	829	-	12,797	-	564	6,110	4,815
75-99	6,072	3,025	814	835	-	16,886	-	819	7,572	8,979
	13,621	5,373	4,468	3,315	-	69,791	-	5,164	18,523	17,857

Number of patients

Age group (years)	Hodgkin's lymphoma		Kidney		Larynx		Leukaemia		Lung	
	Men	Women	Men	Women	Men	Women	Men	Women	Men	Women
15-44	823	631	334	191	80	-	699	469	435	401
45-54	211	79	821	405	445	-	624	423	2,311	1,828
55-64	164	118	1,496	754	906	-	1,208	682	7,115	4,172
65-74	147	118	2,109	1,123	1,037	-	1,887	1,141	13,510	8,037
75-99	100	98	1,854	1,436	779	-	2,427	2,333	14,935	10,075
	1,445	1,044	6,614	3,909	3,247	-	6,845	5,048	38,306	24,513

Number of patients

Age group (years)	Melanoma		Myeloma		Non-Hodgkin lymphoma		Oesophagus		Others	
	Men	Women	Men	Women	Men	Women	Men	Women	Men	Women
15-44	1,144	1,985	96	64	1,040	679	150	52	1,876	2,075
45-54	978	1,278	288	214	1,117	845	782	269	2,756	1,985
55-64	1,175	1,207	696	497	1,775	1,428	1,660	592	4,606	2,964
65-74	1,183	1,175	1,107	878	2,336	1,904	2,439	1,146	5,749	4,220
75-99	1,184	1,556	1,258	1,478	2,320	2,823	2,823	2,716	6,270	7,465
	5,664	7,201	3,445	3,131	8,588	7,679	7,854	4,775	21,257	18,709

Number of patients

Age group (years)	Ovary		Pancreas		Prostate		Rectum		Stomach	
	Men	Women	Men	Women	Men	Women	Men	Women	Men	Women
15-44	-	1,132	129	86	55	-	341	302	242	146
45-54	-	1,860	473	312	1,373	-	1,150	815	627	269
55-64	-	2,800	1,106	820	8,627	-	2,986	1,481	1,632	573
65-74	-	2,950	1,813	1,557	20,724	-	4,579	2,452	3,382	1,413
75-99	-	3,160	2,158	3,200	23,450	-	4,626	4,304	4,370	3,255
	-	11,902	5,679	5,975	54,229	-	13,682	9,354	10,253	5,656

Number of patients

Age group (years)	Testis		Uterus		All cancers	
	Men	Women	Men	Women	Men	Women
15-44	2,678	-	-	305	11,668	20,048
45-54	482	-	-	1,291	17,005	30,230
55-64	175	-	-	3,155	41,753	41,955
65-74	83	-	-	3,025	73,700	51,496
75-99	41	-	-	2,898	83,053	77,341
	3,459	-	-	10,674	227,179	221,070

Appendix Table 1. Number of patients by cancer, age group and sex: diagnosed in England and Wales during 2005-06

	Number of patients									
**Age group**	**Bladder**		**Brain**		**Breast**		**Cervix**		**Colon**	
**(years)**	**Men**	**Women**	**Men**	**Women**	**Men**	**Women**	**Men**	**Women**	**Men**	**Women**
**15-44**	156	84	957	615	-	8,655	-	2,434	568	527
**45-54**	535	205	646	381	-	14,902	-	778	1,158	1,105
**55-64**	2,061	664	1,090	686	-	18,972	-	637	3,545	2,675
**65-74**	4,047	1,302	1,090	764	-	16,076	-	494	6,235	4,805
**75-99**	6,468	2,950	940	954	-	18,213	-	629	8,658	9,814
	13,267	5,205	4,723	3,400	-	76,818	-	4,972	20,164	18,926

	Number of patients									
**Age group**	**Hodgkin's lymphoma**		**Kidney**		**Larynx**		**Leukaemia**		**Lung**	
**(years)**	**Men**	**Women**	**Men**	**Women**	**Men**	**Women**	**Men**	**Women**	**Men**	**Women**
**15-44**	767	638	397	233	69	-	719	508	440	401
**45-54**	210	110	902	469	351	-	581	411	2,015	1,767
**55-64**	214	131	1,944	992	952	-	1,398	754	7,372	5,206
**65-74**	161	153	2,446	1,368	899	-	2,091	1,176	12,340	8,174
**75-99**	145	160	2,728	1,936	769	-	2,659	2,487	15,955	12,201
	1,497	1,192	8,417	4,998	3,040	-	7,448	5,336	38,122	27,749

	Number of patients									
**Age group**	**Melanoma**		**Myeloma**		**Non-Hodgkin lymphoma**		**Oesophagus**		**Others**	
**(years)**	**Men**	**Women**	**Men**	**Women**	**Men**	**Women**	**Men**	**Women**	**Men**	**Women**
**15-44**	1,536	2,543	114	67	1,056	672	152	64	2,165	2,561
**45-54**	1,137	1,541	338	214	1,118	790	724	248	2,925	2,224
**55-64**	1,839	1,778	793	589	2,076	1,668	2,063	702	5,515	3,536
**65-74**	1,839	1,520	1,200	911	2,618	2,261	2,579	1,107	6,401	4,396
**75-99**	1,737	2,028	1,498	1,562	2,966	3,018	3,182	2,668	7,243	7,901
	8,088	9,410	3,943	3,343	9,834	8,409	8,700	4,789	24,249	20,618

	Number of patients									
**Age group**	**Ovary**		**Pancreas**		**Prostate**		**Rectum**		**Stomach**	
**(years)**	**Men**	**Women**	**Men**	**Women**	**Men**	**Women**	**Men**	**Women**	**Men**	**Women**
**15-44**	-	1,249	124	105	96	-	400	355	225	151
**45-54**	-	1,601	470	344	1,906	-	1,231	877	473	202
**55-64**	-	2,896	1,389	1,051	13,560	-	3,343	1,843	1,398	499
**65-74**	-	2,881	1,976	1,764	24,728	-	4,814	2,560	2,729	1,061
**75-99**	-	3,314	2,465	3,495	25,241	-	5,176	4,436	4,033	2,744
	-	11,941	6,424	6,759	65,531	-	14,964	10,071	8,858	4,657

	Number of patients					
**Age group**	**Testis**		**Uterus**		**All cancers**	
**(years)**	**Men**	**Women**	**Men**	**Women**	**Men**	**Women**
**15-44**	2,824	-	-	403	12,765	22,265
**45-54**	496	-	-	1,461	17,216	29,630
**55-64**	172	-	-	3,812	50,724	49,091
**65-74**	88	-	-	3,697	78,281	56,470
**75-99**	43	-	-	3,188	91,906	83,698
	3,623	-	-	12,561	250,892	241,154

Appendix Table 1. Number of patients by cancer, age group and sex: diagnosed in England and Wales during 2010-11

Age group (years)	Bladder Men	Bladder Women	Brain Men	Brain Women	Breast Men	Breast Women	Cervix Men	Cervix Women	Colon Men	Colon Women
**15-44**	133	72	931	648	-	8,698	-	2,699	648	698
**45-54**	510	196	707	495	-	18,066	-	814	1,320	1,207
**55-64**	1,838	608	1,096	801	-	19,353	-	631	4,363	3,328
**65-74**	4,044	1,249	1,262	852	-	16,837	-	421	7,624	5,688
**75-99**	6,800	3,040	1,144	1,135	-	19,435	-	581	10,086	10,858
	13,325	5,165	5,140	3,931	-	82,389	-	5,146	24,041	21,779

Age group (years)	Hodgkin's lymphoma Men	Hodgkin's lymphoma Women	Kidney Men	Kidney Women	Larynx Men	Larynx Women	Leukaemia Men	Leukaemia Women	Lung Men	Lung Women
**15-44**	931	737	543	311	69	-	724	513	449	387
**45-54**	263	134	1,202	622	351	-	769	508	1,888	1,833
**55-64**	236	184	2,471	1,330	947	-	1,665	1,032	7,307	6,009
**65-74**	218	165	3,074	1,748	1,043	-	2,497	1,509	13,342	9,991
**75-99**	156	156	3,544	2,676	850	-	3,320	2,787	17,314	14,320
	1,804	1,376	10,834	6,687	3,260	-	8,975	6,349	40,300	32,540

Age group (years)	Melanoma Men	Melanoma Women	Myeloma Men	Myeloma Women	Non-Hodgkin lymphoma Men	Non-Hodgkin lymphoma Women	Oesophagus Men	Oesophagus Women	Others Men	Others Women
**15-44**	1,531	2,633	112	95	1,147	767	171	51	2,431	3,099
**45-54**	1,592	2,006	390	267	1,305	968	836	242	3,630	2,879
**55-64**	2,366	2,227	895	698	2,497	1,989	2,282	748	6,774	4,420
**65-74**	2,727	2,150	1,492	1,056	3,309	2,662	3,080	1,114	7,868	5,350
**75-99**	2,884	2,602	1,943	1,769	3,737	3,625	3,499	2,658	9,089	8,938
	11,100	11,618	4,832	3,885	11,995	10,011	9,868	4,813	29,792	24,686

Age group (years)	Ovary Men	Ovary Women	Pancreas Men	Pancreas Women	Prostate Men	Prostate Women	Rectum Men	Rectum Women	Stomach Men	Stomach Women
**15-44**	-	1,422	135	124	142	-	421	427	225	147
**45-54**	-	1,793	508	407	2,867	-	1,334	1,052	485	271
**55-64**	-	2,727	1,535	1,181	16,539	-	3,803	2,116	1,202	491
**65-74**	-	3,139	2,290	2,027	29,811	-	5,454	2,737	2,359	962
**75-99**	-	3,448	3,114	3,933	28,299	-	5,479	4,240	3,817	2,457
	-	12,529	7,582	7,672	77,658	-	16,491	10,572	8,088	4,328

Age group (years)	Testis Men	Testis Women	Uterus Men	Uterus Women	All cancers Men	All cancers Women
**15-44**	2,899	-	-	469	13,642	23,997
**45-54**	621	-	-	1,713	20,578	35,473
**55-64**	228	-	-	4,398	58,044	54,271
**65-74**	88	-	-	4,601	91,582	64,258
**75-99**	52	-	-	3,758	105,127	92,416
	3,888	-	-	14,939	288,973	270,415

Number of patients

Appendix Table 1. Number of patients by cancer, age group and sex: diagnosed in England and Wales during 2015-16

Number of patients

Age group (years)	Bladder Men	Bladder Women	Brain Men	Brain Women	Breast Men	Breast Women	Cervix Men	Cervix Women	Colon Men	Colon Women
**15-44**	99	67	1,001	785	-	8,577	-	2,878	839	1,025
**45-54**	477	185	793	525	-	19,484	-	910	1,558	1,437
**55-64**	1,469	493	1,146	746	-	19,515	-	661	4,193	3,175
**65-74**	3,889	1,242	1,433	1,017	-	21,210	-	469	7,705	5,765
**75-99**	7,160	2,981	1,300	1,206	-	20,843	-	515	11,210	11,817
	13,094	4,968	5,673	4,279	-	89,629	-	5,433	25,505	23,219

Number of patients

Age group (years)	Hodgkin's lymphoma Men	Hodgkin's lymphoma Women	Kidney Men	Kidney Women	Larynx Men	Larynx Women	Leukaemia Men	Leukaemia Women	Lung Men	Lung Women
**15-44**	978	779	625	371	65	-	781	559	393	403
**45-54**	288	152	1,644	824	334	-	899	531	1,894	1,900
**55-64**	269	165	2,846	1,423	771	-	1,740	1,052	6,743	6,113
**65-74**	268	229	4,302	2,299	1,205	-	3,098	1,810	14,639	12,736
**75-99**	243	221	4,637	3,337	877	-	3,986	2,959	19,254	16,470
	2,046	1,546	14,054	8,254	3,252	-	10,504	6,911	42,923	37,622

Number of patients

Age group (years)	Melanoma Men	Melanoma Women	Myeloma Men	Myeloma Women	Non-Hodgkin lymphoma Men	Non-Hodgkin lymphoma Women	Oesophagus Men	Oesophagus Women	Others Men	Others Women
**15-44**	1,471	2,601	137	74	1,047	743	152	51	2,388	3,395
**45-54**	1,834	2,441	445	323	1,449	1,014	789	244	4,160	3,360
**55-64**	2,544	2,428	972	684	2,342	1,862	2,287	735	7,147	4,527
**65-74**	3,720	2,955	1,847	1,247	4,024	3,109	3,868	1,370	9,726	6,384
**75-99**	4,229	3,324	2,421	1,992	4,560	4,195	3,974	2,604	10,536	9,037
	13,798	13,749	5,822	4,320	13,422	10,923	11,070	5,004	33,957	26,703

Number of patients

Age group (years)	Ovary Men	Ovary Women	Pancreas Men	Pancreas Women	Prostate Men	Prostate Women	Rectum Men	Rectum Women	Stomach Men	Stomach Women
**15-44**	-	1,529	161	124	176	-	528	499	196	173
**45-54**	-	1,938	606	472	3,610	-	1,374	1,198	493	254
**55-64**	-	2,571	1,522	1,133	16,889	-	3,463	2,202	1,066	509
**65-74**	-	3,423	3,001	2,422	36,135	-	5,164	2,875	2,131	866
**75-99**	-	3,683	3,840	4,659	31,062	-	5,593	4,080	3,734	2,203
	-	13,144	9,130	8,810	87,872	-	16,122	10,854	7,620	4,005

Number of patients

Age group (years)	Testis Men	Testis Women	Uterus Men	Uterus Women	All cancers Men	All cancers Women
**15-44**	2,936	-	-	471	13,973	25,104
**45-54**	650	-	-	1,906	23,297	39,098
**55-64**	256	-	-	4,328	57,665	54,322
**65-74**	118	-	-	5,218	106,273	76,646
**75-99**	51	-	-	4,483	118,667	100,609
	4,011	-	-	16,406	319,875	295,779

Appendix Table 1. Number of patients by cancer, age group and sex: diagnosed in England and Wales during 2018

Number of patients

Age group	Bladder		Brain		Breast		Cervix		Colon	
(years)	Men	Women	Men	Women	Men	Women	Men	Women	Men	Women
15-44	50	31	509	394	-	4,193	-	1,413	407	423
45-54	247	103	400	245	-	9,651	-	510	782	751
55-64	791	254	522	380	-	10,285	-	376	2,305	1,694
65-74	2,023	626	718	491	-	11,185	-	244	3,785	3,047
75-99	3,610	1,385	667	567	-	10,918	-	261	5,677	5,905
	6,721	2,399	2,816	2,077	-	46,232	-	2,804	12,956	11,820

Number of patients

Age group	Hodgkin's lymphoma		Kidney		Larynx		Leukaemia		Lung	
(years)	Men	Women	Men	Women	Men	Women	Men	Women	Men	Women
15-44	472	371	300	183	23	-	406	281	200	209
45-54	154	82	907	418	173	-	400	236	958	907
55-64	160	76	1,466	731	386	-	763	480	3,205	3,041
65-74	151	114	2,289	1,174	569	-	1,386	841	7,382	6,624
75-99	117	126	2,256	1,539	383	-	1,695	1,224	9,454	8,504
	1,054	769	7,218	4,045	1,534	-	4,650	3,062	21,199	19,285

Number of patients

Age group	Melanoma		Myeloma		Non-Hodgkin lymphoma		Oesophagus		Others	
(years)	Men	Women	Men	Women	Men	Women	Men	Women	Men	Women
15-44	715	1,320	53	52	493	355	77	39	1,167	1,722
45-54	928	1,254	251	181	677	473	380	108	2,036	1,766
55-64	1,432	1,304	539	366	1,192	928	1,163	360	3,853	2,454
65-74	2,085	1,705	982	630	2,060	1,606	2,069	677	5,216	3,327
75-99	2,391	1,808	1,276	951	2,349	2,067	2,086	1,308	5,457	4,645
	7,551	7,391	3,101	2,180	6,771	5,429	5,775	2,492	17,729	13,914

Number of patients

Age group	Ovary		Pancreas		Prostate		Rectum		Stomach	
(years)	Men	Women	Men	Women	Men	Women	Men	Women	Men	Women
15-44	-	749	81	70	105	-	308	275	95	71
45-54	-	924	320	234	2,294	-	723	607	249	148
55-64	-	1,326	818	625	11,020	-	1,932	1,178	530	257
65-74	-	1,631	1,588	1,213	22,391	-	2,629	1,405	998	431
75-99	-	1,837	2,030	2,319	16,954	-	2,756	1,952	1,674	1,006
	-	6,467	4,837	4,461	52,764	-	8,348	5,417	3,546	1,913

Number of patients

Age group	Testis		Uterus		All cancers	
(years)	Men	Women	Men	Women	Men	Women
15-44	1,536	-	-	303	6,997	12,454
45-54	362	-	-	964	12,241	19,562
55-64	145	-	-	2,347	32,222	28,462
65-74	71	-	-	2,795	58,392	39,766
75-99	21	-	-	2,405	60,853	50,727
	2,135	-	-	8,814	170,705	150,971

Appendix Table 2. Standardised net survival at 1, 5, 7, and 10 years for all cancers combined (CSI) and for 22 selected cancers, by sex: adults diagnosed in England and Wales during 1971-72

		1 year		5 year		7 year		10 year	
		NS	95% CI	NS	95% CI	NS	95% CI	NS	95% CI
**All cancers combined (cancer survival index)**									
	**All persons**	**46.5**	46.4 - 46.6	**28.8**	28.6 - 29.1	**26.1**	25.8 - 26.4	**23.7**	23.4 - 24.1
	**Men**	**40.9**	40.7 - 41.1	**23.7**	23.0 - 24.4	**21.2**	20.4 - 22.0	**19.2**	18.2 - 20.2
	**Women**	**52.0**	51.9 - 52.2	**33.8**	33.4 - 34.2	**30.9**	30.4 - 31.3	**28.2**	27.6 - 28.8
**Bladder**									
	**All persons**	**59.0**	58.8 - 59.2	**41.2**	40.5 - 41.9	**38.5**	37.7 - 39.3	**35.4**	34.4 - 36.5
	**Men**	**61.6**	61.4 - 61.8	**42.4**	41.7 - 43.2	**39.8**	38.9 - 40.8	**36.9**	35.8 - 38.1
	**Women**	**52.4**	51.9 - 53.0	**38.1**	36.7 - 39.5	**35.1**	33.5 - 36.7	**31.6**	29.6 - 33.6
**Brain**									
	**All persons**	**18.3**	16.3 - 20.5	**8.4**	5.2 - 12.5	**7.4**	4.1 - 12.1	**6.6**	3.1 - 12.0
	**Men**	**18.5**	15.9 - 21.2	**8.1**	4.1 - 13.8	**7.2**	3.1 - 13.6	**6.5**	2.3 - 14.0
	**Women**	**18.1**	14.9 - 21.6	**8.8**	4.2 - 15.5	**7.7**	3.1 - 15.3	**6.8**	2.0 - 15.6
**Breast**									
	**All persons**	**78.4**	78.4 - 78.4	**53.2**	53.1 - 53.4	**47.6**	47.3 - 47.8	**42.0**	41.6 - 42.4
	**Men**								
	**Women**	**78.4**	78.4 - 78.4	**53.2**	53.1 - 53.4	**47.6**	47.3 - 47.8	**42.0**	41.6 - 42.4
**Cervix**									
	**All persons**	**73.3**	73.3 - 73.4	**53.5**	53.1 - 54.0	**51.0**	50.5 - 51.6	**48.2**	47.5 - 48.8
	**Men**								
	**Women**	**73.3**	73.3 - 73.4	**53.5**	53.1 - 54.0	**51.0**	50.5 - 51.6	**48.2**	47.5 - 48.8
**Colon**									
	**All persons**	**38.3**	37.8 - 38.7	**23.3**	22.2 - 24.4	**22.4**	21.1 - 23.7	**22.4**	20.9 - 23.9
	**Men**	**39.4**	38.7 - 40.1	**24.1**	22.5 - 25.8	**23.1**	21.2 - 25.1	**23.0**	20.6 - 25.4
	**Women**	**37.1**	36.5 - 37.8	**22.5**	21.0 - 24.0	**21.7**	20.1 - 23.4	**21.8**	19.9 - 23.6
**Hodgkin's lymphoma**									
	**All persons**	**73.7**	73.6 - 73.9	**56.9**	56.3 - 57.6	**53.3**	52.4 - 54.1	**48.6**	47.4 - 49.8
	**Men**	**71.9**	71.7 - 72.2	**54.8**	53.8 - 55.8	**51.3**	50.0 - 52.6	**46.7**	45.0 - 48.4
	**Women**	**76.1**	76.0 - 76.3	**59.8**	58.9 - 60.6	**55.9**	54.8 - 57.0	**51.1**	49.5 - 52.6
**Kidney**									
	**All persons**	**40.4**	39.5 - 41.2	**26.8**	25.0 - 28.5	**24.5**	22.4 - 26.6	**22.2**	19.7 - 24.8
	**Men**	**41.2**	40.2 - 42.2	**27.5**	25.4 - 29.7	**25.2**	22.7 - 27.8	**22.9**	19.7 - 26.1
	**Women**	**39.0**	37.5 - 40.4	**25.5**	22.6 - 28.5	**23.3**	19.9 - 26.8	**21.0**	17.0 - 25.3
**Larynx**									
	**All persons**	**76.2**	76.1 - 76.3	**59.5**	59.0 - 60.0	**57.5**	56.9 - 58.1	**54.2**	53.4 - 55.0
	**Men**	**76.2**	76.1 - 76.3	**59.5**	59.0 - 60.0	**57.5**	56.9 - 58.1	**54.2**	53.4 - 55.0
	**Women**								
**Leukaemia**									
	**All persons**	**36.8**	36.0 - 37.7	**16.3**	14.2 - 18.5	**12.6**	10.2 - 15.4	**9.4**	6.6 - 12.9
	**Men**	**38.8**	37.8 - 39.8	**15.9**	13.3 - 18.8	**11.9**	9.0 - 15.3	**8.5**	5.3 - 12.5
	**Women**	**34.3**	32.8 - 35.8	**16.7**	13.4 - 20.3	**13.6**	9.7 - 18.0	**10.6**	6.1 - 16.5
**Lung**									
	**All persons**	**14.9**	14.2 - 15.6	**4.7**	3.7 - 5.8	**3.9**	2.9 - 5.1	**3.3**	2.3 - 4.7
	**Men**	**15.4**	14.6 - 16.3	**5.2**	4.1 - 6.5	**4.3**	3.1 - 5.8	**3.6**	2.3 - 5.3
	**Women**	**13.9**	12.5 - 15.4	**3.9**	2.3 - 6.1	**3.3**	1.7 - 5.7	**2.9**	1.3 - 5.5
**Melanoma**									
	**All persons**	**77.9**	77.8 - 78.0	**52.2**	51.5 - 52.8	**48.7**	47.9 - 49.5	**46.4**	45.4 - 47.4
	**Men**	**70.3**	70.0 - 70.5	**40.2**	38.1 - 42.2	**36.7**	34.2 - 39.3	**34.6**	31.4 - 37.8
	**Women**	**83.6**	83.5 - 83.6	**61.0**	60.5 - 61.5	**57.5**	56.9 - 58.1	**55.2**	54.4 - 55.9
**Myeloma**									
	**All persons**	**36.8**	35.5 - 38.1	**14.0**	10.4 - 18.1	**11.3**	7.1 - 16.6	**9.3**	4.2 - 16.9
	**Men**	**36.2**	34.3 - 38.0	**14.5**	9.7 - 20.2	**12.0**	6.4 - 19.5	**10.2**	3.5 - 20.9
	**Women**	**37.5**	35.6 - 39.4	**13.3**	8.3 - 19.6	**10.5**	4.8 - 18.7	**8.3**	2.1 - 20.3
**Non-Hodgkin lymphoma**									
	**All persons**	**48.9**	48.4 - 49.4	**30.9**	29.7 - 32.2	**27.0**	25.5 - 28.5	**22.8**	21.0 - 24.7
	**Men**	**48.6**	48.0 - 49.3	**30.2**	28.4 - 31.9	**26.4**	24.3 - 28.5	**22.4**	19.9 - 25.1
	**Women**	**49.2**	48.5 - 49.9	**31.8**	30.0 - 33.6	**27.7**	25.6 - 29.9	**23.3**	20.7 - 26.0
**Oesophagus**									
	**All persons**	**14.7**	12.7 - 16.9	**5.0**	2.4 - 9.1	**4.6**	1.9 - 9.2	**4.5**	1.5 - 9.8
	**Men**	**14.2**	11.6 - 17.0	**4.7**	1.7 - 10.2	**4.4**	1.3 - 10.7	**4.3**	1.0 - 11.7
	**Women**	**15.6**	12.4 - 19.1	**5.5**	1.8 - 12.4	**5.0**	1.2 - 13.1	**4.7**	0.8 - 14.4
**Others**									
	**All persons**	**40.2**	39.8 - 40.5	**27.0**	26.2 - 27.7	**25.7**	24.9 - 26.6	**24.9**	23.9 - 25.9
	**Men**	**41.3**	40.8 - 41.8	**28.0**	27.0 - 29.0	**26.8**	25.6 - 28.0	**26.0**	24.7 - 27.4
	**Women**	**38.9**	38.4 - 39.4	**25.8**	24.7 - 26.9	**24.5**	23.2 - 25.8	**23.5**	22.0 - 25.0
**Ovary**									
	**All persons**	**41.4**	40.8 - 42.0	**19.8**	18.0 - 21.6	**18.3**	16.3 - 20.5	**17.7**	15.4 - 20.2
	**Men**								
	**Women**	**41.4**	40.8 - 42.0	**19.8**	18.0 - 21.6	**18.3**	16.3 - 20.5	**17.7**	15.4 - 20.2
**Pancreas**									
	**All persons**	**9.0**	6.8 - 11.6	**3.1**	0.7 - 8.8	**2.7**	0.3 - 10.1	**2.4**	0.1 - 12.3
	**Men**	**8.9**	6.0 - 12.4	**3.1**	0.4 - 11.3	**2.7**	0.2 - 13.4	**2.4**	0.0 - 16.7
	**Women**	**9.1**	6.0 - 13.0	**3.1**	0.2 - 13.4	**2.7**	0.1 - 16.5	**2.4**	0.0 - 21.4

Appendix Table 2. Standardised net survival at 1, 5, 7, and 10 years for all cancers combined (CSI) and for 22 selected cancers, by sex: adults diagnosed in England and Wales during 1971-72

		1 year		5 year		7 year		10 year	
		NS	95% CI	NS	95% CI	NS	95% CI	NS	95% CI
Prostate									
	All persons	60.4	60.2 - 60.6	32.9	31.9 - 34.0	26.5	25.1 - 28.0	21.7	19.8 - 23.7
	Men	60.4	60.2 - 60.6	32.9	31.9 - 34.0	26.5	25.1 - 28.0	21.7	19.8 - 23.7
	Women								
Rectum									
	All persons	48.5	48.2 - 48.8	27.4	26.3 - 28.4	25.4	24.1 - 26.7	24.6	23.0 - 26.2
	Men	49.5	49.1 - 49.9	27.9	26.5 - 29.4	25.9	24.1 - 27.6	24.9	22.8 - 27.1
	Women	47.0	46.5 - 47.6	26.5	24.9 - 28.2	24.7	22.7 - 26.7	24.0	21.7 - 26.5
Stomach									
	All persons	13.9	12.5 - 15.4	5.2	3.4 - 7.7	4.7	2.7 - 7.4	4.3	2.2 - 7.5
	Men	14.2	12.5 - 16.0	5.3	3.1 - 8.5	4.8	2.4 - 8.4	4.4	1.8 - 8.6
	Women	13.4	11.1 - 15.9	5.1	2.4 - 9.3	4.5	1.7 - 9.4	4.1	1.2 - 9.8
Testis									
	All persons	78.7	78.6 - 78.8	64.0	63.3 - 64.7	62.8	62.0 - 63.6	62.5	61.6 - 63.3
	Men	78.7	78.6 - 78.8	64.0	63.3 - 64.7	62.8	62.0 - 63.6	62.5	61.6 - 63.3
	Women								
Uterus									
	All persons	72.5	72.4 - 72.6	58.5	58.1 - 58.8	57.1	56.8 - 57.4	55.6	55.2 - 56.0
	Men								
	Women	72.5	72.4 - 72.6	58.5	58.1 - 58.8	57.1	56.8 - 57.4	55.6	55.2 - 56.0

Appendix Table 2. Standardised net survival at 1, 5, 7, and 10 years for all cancers combined (CSI) and for 22 selected cancers, by sex: adults diagnosed in England and Wales during 1980-81

		1 year		5 year		7 year		10 year	
		NS	95% CI	NS	95% CI	NS	95% CI	NS	95% CI
**All cancers combined (cancer survival index)**									
	**All persons**	**51.8**	51.7 - 51.8	**32.9**	32.8 - 33.0	**29.8**	29.6 - 29.9	**27.0**	26.8 - 27.2
	**Men**	**46.6**	46.5 - 46.7	**27.6**	27.3 - 27.9	**24.5**	24.1 - 24.9	**21.9**	21.4 - 22.4
	**Women**	**56.8**	56.7 - 56.8	**38.1**	37.9 - 38.3	**34.9**	34.7 - 35.2	**31.9**	31.6 - 32.2
**Bladder**									
	**All persons**	**69.7**	69.6 - 69.7	**52.5**	52.3 - 52.7	**49.2**	48.9 - 49.4	**45.1**	44.8 - 45.5
	**Men**	**72.7**	72.7 - 72.8	**54.5**	54.3 - 54.7	**51.0**	50.7 - 51.3	**46.8**	46.4 - 47.2
	**Women**	**61.9**	61.7 - 62.1	**47.3**	46.8 - 47.8	**44.3**	43.7 - 44.9	**40.9**	40.0 - 41.7
**Brain**									
	**All persons**	**22.3**	21.2 - 23.3	**10.0**	8.0 - 12.2	**8.6**	6.5 - 11.1	**7.5**	5.1 - 10.3
	**Men**	**22.3**	20.9 - 23.7	**9.4**	6.8 - 12.5	**8.1**	5.3 - 11.6	**7.0**	4.1 - 11.1
	**Women**	**22.3**	20.7 - 24.0	**10.7**	7.9 - 14.1	**9.3**	6.2 - 13.2	**8.0**	4.7 - 12.5
**Breast**									
	**All persons**	**81.5**	81.5 - 81.5	**58.7**	58.6 - 58.8	**53.1**	53.0 - 53.2	**47.3**	47.1 - 47.5
	**Men**								
	**Women**	**81.5**	81.5 - 81.5	**58.7**	58.6 - 58.8	**53.1**	53.0 - 53.2	**47.3**	47.1 - 47.5
**Cervix**									
	**All persons**	**76.0**	76.0 - 76.1	**55.9**	55.6 - 56.2	**53.0**	52.7 - 53.4	**49.9**	49.5 - 50.3
	**Men**								
	**Women**	**76.0**	76.0 - 76.1	**55.9**	55.6 - 56.2	**53.0**	52.7 - 53.4	**49.9**	49.5 - 50.3
**Colon**									
	**All persons**	**48.3**	48.1 - 48.5	**29.6**	29.1 - 30.1	**28.1**	27.4 - 28.7	**27.5**	26.7 - 28.3
	**Men**	**49.8**	49.6 - 50.1	**30.4**	29.6 - 31.1	**28.6**	27.6 - 29.6	**27.8**	26.6 - 29.1
	**Women**	**46.8**	46.6 - 47.0	**28.8**	28.1 - 29.5	**27.5**	26.7 - 28.3	**27.1**	26.1 - 28.1
**Hodgkin's lymphoma**									
	**All persons**	**81.1**	81.1 - 81.1	**65.7**	65.4 - 65.9	**62.1**	61.8 - 62.4	**57.8**	57.4 - 58.2
	**Men**	**80.7**	80.6 - 80.7	**64.3**	64.0 - 64.6	**60.6**	60.2 - 61.1	**56.1**	55.5 - 56.8
	**Women**	**81.6**	81.6 - 81.7	**67.4**	67.2 - 67.7	**64.1**	63.7 - 64.5	**59.9**	59.4 - 60.5
**Kidney**									
	**All persons**	**46.1**	45.8 - 46.5	**30.5**	29.6 - 31.3	**27.6**	26.5 - 28.7	**24.6**	23.2 - 26.0
	**Men**	**47.4**	47.0 - 47.9	**31.5**	30.4 - 32.6	**28.5**	27.2 - 29.9	**25.5**	23.7 - 27.2
	**Women**	**43.9**	43.3 - 44.6	**28.7**	27.2 - 30.2	**25.9**	24.1 - 27.8	**23.1**	20.9 - 25.4
**Larynx**									
	**All persons**	**78.4**	78.4 - 78.5	**59.2**	58.9 - 59.5	**56.4**	55.9 - 56.8	**52.2**	51.6 - 52.8
	**Men**	**78.4**	78.4 - 78.5	**59.2**	58.9 - 59.5	**56.4**	55.9 - 56.8	**52.2**	51.6 - 52.8
	**Women**								
**Leukaemia**									
	**All persons**	**44.7**	44.3 - 45.0	**22.3**	21.2 - 23.4	**18.1**	16.7 - 19.4	**14.1**	12.5 - 15.9
	**Men**	**46.0**	45.6 - 46.4	**21.9**	20.5 - 23.3	**17.4**	15.8 - 19.1	**13.4**	11.4 - 15.4
	**Women**	**43.0**	42.4 - 43.6	**22.8**	21.1 - 24.5	**18.9**	16.8 - 21.1	**15.1**	12.3 - 18.1
**Lung**									
	**All persons**	**16.7**	16.3 - 17.1	**4.9**	4.3 - 5.5	**4.0**	3.3 - 4.6	**3.2**	2.5 - 4.0
	**Men**	**17.1**	16.6 - 17.5	**5.3**	4.6 - 6.0	**4.2**	3.5 - 5.0	**3.3**	2.6 - 4.2
	**Women**	**16.0**	15.3 - 16.8	**4.3**	3.2 - 5.5	**3.5**	2.4 - 4.9	**3.0**	1.9 - 4.5
**Melanoma**									
	**All persons**	**85.8**	85.8 - 85.8	**64.2**	64.0 - 64.3	**60.7**	60.5 - 60.9	**58.3**	58.0 - 58.6
	**Men**	**81.1**	81.0 - 81.1	**54.0**	53.4 - 54.5	**50.2**	49.4 - 50.9	**47.7**	46.7 - 48.7
	**Women**	**89.3**	89.3 - 89.3	**71.7**	71.6 - 71.8	**68.5**	68.3 - 68.6	**66.2**	66.0 - 66.4
**Myeloma**									
	**All persons**	**45.3**	44.8 - 45.8	**15.9**	13.9 - 17.9	**11.8**	9.3 - 14.5	**8.5**	5.3 - 12.6
	**Men**	**45.0**	44.3 - 45.6	**16.0**	13.3 - 18.9	**12.0**	8.7 - 15.9	**8.9**	4.7 - 14.7
	**Women**	**45.7**	45.0 - 46.4	**15.8**	13.0 - 18.7	**11.5**	8.1 - 15.6	**8.1**	3.9 - 14.2
**Non-Hodgkin lymphoma**									
	**All persons**	**55.6**	55.4 - 55.8	**35.1**	34.5 - 35.7	**30.7**	29.9 - 31.4	**26.0**	25.0 - 27.0
	**Men**	**55.5**	55.2 - 55.7	**34.4**	33.5 - 35.3	**30.1**	29.0 - 31.1	**25.6**	24.2 - 26.9
	**Women**	**55.8**	55.5 - 56.0	**35.8**	35.0 - 36.7	**31.4**	30.3 - 32.4	**26.5**	25.1 - 27.9
**Oesophagus**									
	**All persons**	**17.7**	16.7 - 18.8	**4.9**	3.3 - 6.9	**4.2**	2.6 - 6.6	**3.9**	2.1 - 6.6
	**Men**	**17.4**	16.1 - 18.8	**4.5**	2.5 - 7.2	**3.9**	1.9 - 7.1	**3.6**	1.5 - 7.3
	**Women**	**18.2**	16.6 - 20.0	**5.5**	3.1 - 8.8	**4.8**	2.3 - 8.6	**4.3**	1.7 - 9.0
**Others**									
	**All persons**	**44.7**	44.5 - 44.9	**28.9**	28.4 - 29.3	**27.0**	26.4 - 27.5	**25.3**	24.7 - 26.0
	**Men**	**44.1**	43.9 - 44.3	**27.7**	27.0 - 28.3	**25.8**	25.0 - 26.5	**24.2**	23.2 - 25.2
	**Women**	**45.4**	45.1 - 45.6	**30.3**	29.7 - 30.8	**28.3**	27.6 - 29.0	**26.6**	25.8 - 27.5
**Ovary**									
	**All persons**	**47.7**	47.4 - 47.9	**22.8**	21.8 - 23.8	**20.7**	19.6 - 21.9	**19.4**	18.1 - 20.8
	**Men**								
	**Women**	**47.7**	47.4 - 47.9	**22.8**	21.8 - 23.8	**20.7**	19.6 - 21.9	**19.4**	18.1 - 20.8
**Pancreas**									
	**All persons**	**9.6**	8.3 - 11.0	**2.5**	0.9 - 5.4	**2.0**	0.5 - 5.8	**1.7**	0.2 - 6.7
	**Men**	**9.9**	8.2 - 11.8	**2.7**	0.8 - 6.7	**2.2**	0.4 - 7.4	**1.8**	0.1 - 8.8
	**Women**	**9.3**	7.4 - 11.5	**2.4**	0.4 - 7.6	**1.9**	0.1 - 9.0	**1.6**	0.0 - 11.8

Appendix Table 2. Standardised net survival at 1, 5, 7, and 10 years for all cancers combined (CSI) and for 22 selected cancers, by sex: adults diagnosed in England and Wales during 1980-81

		1 year		5 year		7 year		10 year	
		NS	95% CI	NS	95% CI	NS	95% CI	NS	95% CI
**Prostate**									
	**All persons**	**66.5**	66.4 - 66.5	**38.0**	37.5 - 38.4	**30.8**	30.1 - 31.5	**25.3**	24.3 - 26.3
	**Men**	**66.5**	66.4 - 66.5	**38.0**	37.5 - 38.4	**30.8**	30.1 - 31.5	**25.3**	24.3 - 26.3
	**Women**								
**Rectum**									
	**All persons**	**56.2**	56.1 - 56.3	**30.8**	30.2 - 31.3	**27.9**	27.2 - 28.6	**26.2**	25.3 - 27.1
	**Men**	**57.2**	57.1 - 57.4	**30.9**	30.1 - 31.6	**27.9**	26.9 - 28.8	**26.1**	24.9 - 27.4
	**Women**	**54.8**	54.6 - 55.0	**30.6**	29.8 - 31.5	**27.9**	26.9 - 29.0	**26.3**	25.0 - 27.7
**Stomach**									
	**All persons**	**17.9**	17.2 - 18.7	**6.8**	5.6 - 8.2	**6.0**	4.7 - 7.5	**5.4**	3.9 - 7.2
	**Men**	**18.1**	17.2 - 19.1	**6.8**	5.3 - 8.5	**6.0**	4.4 - 7.9	**5.4**	3.6 - 7.7
	**Women**	**17.5**	16.2 - 18.9	**6.9**	4.9 - 9.3	**6.1**	4.0 - 8.8	**5.4**	3.1 - 8.6
**Testis**									
	**All persons**	**90.0**	90.0 - 90.0	**82.1**	82.0 - 82.1	**81.2**	81.2 - 81.3	**80.9**	80.8 - 80.9
	**Men**	**90.0**	90.0 - 90.0	**82.1**	82.0 - 82.1	**81.2**	81.2 - 81.3	**80.9**	80.8 - 80.9
	**Women**								
**Uterus**									
	**All persons**	**76.0**	76.0 - 76.1	**61.6**	61.4 - 61.7	**59.8**	59.6 - 60.0	**57.8**	57.6 - 58.0
	**Men**								
	**Women**	**76.0**	76.0 - 76.1	**61.6**	61.4 - 61.7	**59.8**	59.6 - 60.0	**57.8**	57.6 - 58.0

Appendix Table 2. Standardised net survival at 1, 5, 7, and 10 years for all cancers combined (CSI) and for 22 selected cancers, by sex: adults diagnosed in England and Wales during 1990-91

		1 year		5 year		7 year		10 year	
		**NS**	**95% CI**	**NS**	**95% CI**	**NS**	**95% CI**	**NS**	**95% CI**
**All cancers combined (cancer survival index)**									
	**All persons**	**57.9**	57.9 - 57.9	**38.9**	38.8 - 39.0	**35.6**	35.5 - 35.7	**32.5**	32.4 - 32.6
	**Men**	**53.1**	53.0 - 53.1	**33.2**	33.0 - 33.4	**29.8**	29.5 - 30.0	**26.8**	26.5 - 27.2
	**Women**	**62.6**	62.6 - 62.6	**44.5**	44.4 - 44.6	**41.3**	41.1 - 41.4	**38.1**	37.9 - 38.3
**Bladder**									
	**All persons**	**74.9**	74.9 - 74.9	**58.2**	58.0 - 58.3	**54.8**	54.6 - 54.9	**50.6**	50.4 - 50.9
	**Men**	**78.1**	78.1 - 78.1	**60.7**	60.6 - 60.9	**57.1**	56.9 - 57.3	**52.7**	52.4 - 53.0
	**Women**	**66.7**	66.6 - 66.8	**51.5**	51.2 - 51.9	**48.6**	48.2 - 49.1	**45.3**	44.7 - 45.9
**Brain**									
	**All persons**	**27.4**	26.7 - 28.1	**12.4**	10.9 - 14.0	**10.7**	9.0 - 12.5	**9.2**	7.3 - 11.3
	**Men**	**27.3**	26.4 - 28.2	**11.6**	9.6 - 13.9	**9.9**	7.7 - 12.5	**8.5**	6.0 - 11.5
	**Women**	**27.5**	26.4 - 28.5	**13.6**	11.4 - 15.9	**11.8**	9.4 - 14.4	**10.1**	7.4 - 13.2
**Breast**									
	**All persons**	**86.6**	86.6 - 86.6	**68.1**	68.1 - 68.1	**63.1**	63.0 - 63.1	**57.6**	57.5 - 57.6
	**Men**								
	**Women**	**86.6**	86.6 - 86.6	**68.1**	68.1 - 68.1	**63.1**	63.0 - 63.1	**57.6**	57.5 - 57.6
**Cervix**									
	**All persons**	**79.3**	79.3 - 79.3	**60.0**	59.8 - 60.1	**57.2**	57.0 - 57.4	**54.3**	54.0 - 54.5
	**Men**								
	**Women**	**79.3**	79.3 - 79.3	**60.0**	59.8 - 60.1	**57.2**	57.0 - 57.4	**54.3**	54.0 - 54.5
**Colon**									
	**All persons**	**58.3**	58.2 - 58.4	**38.0**	37.7 - 38.3	**36.0**	35.6 - 36.4	**34.9**	34.4 - 35.4
	**Men**	**59.9**	59.8 - 60.0	**38.5**	38.1 - 39.0	**36.2**	35.6 - 36.8	**34.9**	34.1 - 35.7
	**Women**	**56.8**	56.6 - 56.9	**37.5**	37.0 - 37.9	**35.7**	35.2 - 36.3	**34.9**	34.2 - 35.6
**Hodgkin's lymphoma**									
	**All persons**	**86.5**	86.5 - 86.5	**73.8**	73.7 - 73.9	**71.0**	70.9 - 71.1	**67.6**	67.4 - 67.7
	**Men**	**86.4**	86.3 - 86.4	**73.2**	73.1 - 73.3	**70.2**	70.0 - 70.4	**66.5**	66.3 - 66.8
	**Women**	**86.6**	86.6 - 86.6	**74.7**	74.5 - 74.8	**72.1**	71.9 - 72.2	**69.0**	68.8 - 69.2
**Kidney**									
	**All persons**	**53.3**	53.1 - 53.5	**35.9**	35.3 - 36.5	**32.4**	31.8 - 33.1	**28.9**	27.9 - 29.8
	**Men**	**54.9**	54.7 - 55.1	**37.0**	36.4 - 37.7	**33.5**	32.6 - 34.3	**29.7**	28.6 - 30.9
	**Women**	**50.7**	50.3 - 51.1	**34.0**	33.0 - 35.0	**30.8**	29.6 - 31.9	**27.4**	25.9 - 28.9
**Larynx**									
	**All persons**	**81.2**	81.2 - 81.3	**61.1**	60.9 - 61.4	**57.6**	57.3 - 57.9	**52.6**	52.1 - 53.1
	**Men**	**81.2**	81.2 - 81.3	**61.1**	60.9 - 61.4	**57.6**	57.3 - 57.9	**52.6**	52.1 - 53.1
	**Women**								
**Leukaemia**									
	**All persons**	**53.4**	53.2 - 53.6	**30.5**	29.8 - 31.1	**25.8**	25.0 - 26.7	**21.4**	20.3 - 22.5
	**Men**	**54.6**	54.4 - 54.8	**30.4**	29.6 - 31.3	**25.6**	24.6 - 26.7	**21.1**	19.8 - 22.4
	**Women**	**51.9**	51.6 - 52.3	**30.5**	29.4 - 31.6	**26.1**	24.7 - 27.5	**21.7**	19.9 - 23.6
**Lung**									
	**All persons**	**19.4**	19.0 - 19.7	**5.3**	4.8 - 5.9	**4.2**	3.6 - 4.8	**3.2**	2.6 - 3.9
	**Men**	**19.3**	18.9 - 19.7	**5.4**	4.8 - 6.1	**4.2**	3.5 - 5.0	**3.2**	2.4 - 4.1
	**Women**	**19.4**	18.8 - 20.0	**5.1**	4.2 - 6.2	**4.1**	3.2 - 5.3	**3.4**	2.4 - 4.6
**Melanoma**									
	**All persons**	**91.4**	91.4 - 91.5	**75.0**	74.9 - 75.0	**72.0**	71.9 - 72.1	**70.0**	69.9 - 70.1
	**Men**	**88.8**	88.8 - 88.9	**67.3**	67.2 - 67.5	**63.8**	63.6 - 64.0	**61.6**	61.2 - 61.9
	**Women**	**93.4**	93.4 - 93.4	**80.6**	80.6 - 80.7	**78.0**	78.0 - 78.1	**76.2**	76.1 - 76.2
**Myeloma**									
	**All persons**	**54.6**	54.3 - 54.8	**20.0**	18.6 - 21.4	**14.3**	12.5 - 16.2	**9.6**	7.2 - 12.4
	**Men**	**54.7**	54.4 - 55.0	**20.0**	18.2 - 21.9	**14.4**	12.0 - 17.1	**9.8**	6.6 - 13.7
	**Women**	**54.4**	54.0 - 54.8	**20.0**	17.9 - 22.1	**14.2**	11.6 - 17.1	**9.4**	6.1 - 13.6
**Non-Hodgkin lymphoma**									
	**All persons**	**63.1**	63.0 - 63.2	**42.1**	41.7 - 42.4	**37.4**	36.9 - 37.8	**32.3**	31.7 - 32.9
	**Men**	**63.0**	62.9 - 63.1	**41.4**	41.0 - 41.9	**36.8**	36.1 - 37.4	**31.8**	31.0 - 32.7
	**Women**	**63.2**	63.1 - 63.3	**42.8**	42.3 - 43.3	**38.1**	37.4 - 38.7	**32.9**	32.0 - 33.8
**Oesophagus**									
	**All persons**	**22.9**	22.1 - 23.7	**5.6**	4.0 - 7.4	**4.7**	3.1 - 6.8	**4.2**	2.4 - 6.7
	**Men**	**23.0**	22.0 - 24.0	**5.2**	3.4 - 7.6	**4.4**	2.5 - 7.2	**3.9**	1.9 - 7.2
	**Women**	**22.7**	21.3 - 24.1	**6.1**	3.7 - 9.3	**5.2**	2.7 - 8.9	**4.6**	2.0 - 9.0
**Others**									
	**All persons**	**49.5**	49.4 - 49.7	**31.5**	31.2 - 31.9	**29.1**	28.7 - 29.5	**26.9**	26.4 - 27.4
	**Men**	**48.0**	47.8 - 48.2	**28.8**	28.3 - 29.4	**26.4**	25.7 - 27.0	**24.2**	23.4 - 25.0
	**Women**	**51.3**	51.1 - 51.4	**34.6**	34.2 - 35.1	**32.2**	31.7 - 32.7	**29.9**	29.3 - 30.5
**Ovary**									
	**All persons**	**55.4**	55.2 - 55.5	**28.6**	28.0 - 29.3	**25.9**	25.1 - 26.7	**24.0**	23.1 - 25.0
	**Men**								
	**Women**	**55.4**	55.2 - 55.5	**28.6**	28.0 - 29.3	**25.9**	25.1 - 26.7	**24.0**	23.1 - 25.0
**Pancreas**									
	**All persons**	**10.9**	9.7 - 12.2	**2.4**	1.0 - 4.9	**1.9**	0.5 - 5.0	**1.5**	0.2 - 5.5
	**Men**	**11.5**	9.8 - 13.2	**2.6**	0.8 - 6.3	**2.0**	0.4 - 6.6	**1.6**	0.1 - 7.6
	**Women**	**10.4**	8.6 - 12.3	**2.2**	0.5 - 6.5	**1.7**	0.2 - 7.4	**1.4**	0.0 - 9.3

Appendix Table 2. Standardised net survival at 1, 5, 7, and 10 years for all cancers combined (CSI) and for 22 selected cancers, by sex: adults diagnosed in England and Wales during 1990-91

		1 year		5 year		7 year		10 year	
		NS	95% CI	NS	95% CI	NS	95% CI	NS	95% CI
**Prostate**									
	**All persons**	**75.8**	75.8 - 75.8	**49.8**	49.6 - 50.0	**42.5**	42.1 - 42.8	**36.3**	35.8 - 36.8
	**Men**	**75.8**	75.8 - 75.8	**49.8**	49.6 - 50.0	**42.5**	42.1 - 42.8	**36.3**	35.8 - 36.8
	**Women**								
**Rectum**									
	**All persons**	**65.3**	65.2 - 65.3	**38.1**	37.8 - 38.5	**34.6**	34.1 - 35.1	**32.3**	31.6 - 32.9
	**Men**	**66.1**	66.0 - 66.1	**37.7**	37.2 - 38.2	**34.0**	33.3 - 34.6	**31.6**	30.7 - 32.5
	**Women**	**64.2**	64.1 - 64.3	**38.8**	38.2 - 39.3	**35.4**	34.7 - 36.1	**33.2**	32.3 - 34.2
**Stomach**									
	**All persons**	**25.2**	24.6 - 25.7	**9.8**	8.7 - 10.9	**8.6**	7.4 - 9.9	**7.7**	6.3 - 9.3
	**Men**	**25.5**	24.9 - 26.2	**9.6**	8.3 - 11.1	**8.5**	7.0 - 10.1	**7.6**	5.9 - 9.6
	**Women**	**24.5**	23.6 - 25.5	**10.0**	8.3 - 12.0	**8.8**	6.9 - 11.1	**7.9**	5.7 - 10.6
**Testis**									
	**All persons**	**95.6**	95.6 - 95.6	**91.9**	91.8 - 91.9	**91.4**	91.4 - 91.4	**91.1**	91.1 - 91.1
	**Men**	**95.6**	95.6 - 95.6	**91.9**	91.8 - 91.9	**91.4**	91.4 - 91.4	**91.1**	91.1 - 91.1
	**Women**								
**Uterus**									
	**All persons**	**80.6**	80.6 - 80.6	**66.0**	65.9 - 66.1	**63.9**	63.8 - 64.1	**61.7**	61.5 - 61.9
	**Men**								
	**Women**	**80.6**	80.6 - 80.6	**66.0**	65.9 - 66.1	**63.9**	63.8 - 64.1	**61.7**	61.5 - 61.9

Appendix Table 2. Standardised net survival at 1, 5, 7, and 10 years for all cancers combined (CSI) and for 22 selected cancers, by sex: adults diagnosed in England and Wales during 2000-01

		1 year		5 year		7 year		10 year	
		NS	95% CI	NS	95% CI	NS	95% CI	NS	95% CI
**All cancers combined (cancer survival index)**									
	**All persons**	**64.6**	64.6 - 64.6	**47.0**	47.0 - 47.1	**44.0**	43.9 - 44.1	**41.2**	41.1 - 41.2
	**Men**	**60.3**	60.3 - 60.3	**41.5**	41.3 - 41.6	**38.3**	38.1 - 38.4	**35.4**	35.2 - 35.6
	**Women**	**68.8**	68.8 - 68.8	**52.5**	52.4 - 52.6	**49.6**	49.5 - 49.7	**46.8**	46.7 - 46.9
**Bladder**									
	**All persons**	**74.7**	74.7 - 74.8	**56.4**	56.3 - 56.6	**53.2**	53.0 - 53.4	**49.6**	49.3 - 49.9
	**Men**	**78.5**	78.5 - 78.5	**59.7**	59.5 - 59.8	**56.4**	56.2 - 56.6	**52.6**	52.3 - 52.9
	**Women**	**65.1**	65.0 - 65.3	**48.1**	47.6 - 48.5	**45.1**	44.5 - 45.6	**41.8**	41.0 - 42.5
**Brain**									
	**All persons**	**33.0**	32.6 - 33.5	**15.6**	14.5 - 16.8	**13.6**	12.3 - 15.0	**11.9**	10.3 - 13.6
	**Men**	**33.3**	32.7 - 33.9	**14.8**	13.2 - 16.4	**12.8**	11.0 - 14.8	**11.1**	9.0 - 13.5
	**Women**	**32.7**	32.0 - 33.4	**16.8**	15.2 - 18.6	**14.8**	12.9 - 16.8	**12.9**	10.7 - 15.4
**Breast**									
	**All persons**	**92.7**	92.7 - 92.7	**80.2**	80.2 - 80.2	**76.4**	76.4 - 76.4	**72.0**	72.0 - 72.1
	**Men**								
	**Women**	**92.7**	92.7 - 92.7	**80.2**	80.2 - 80.2	**76.4**	76.4 - 76.4	**72.0**	72.0 - 72.1
**Cervix**									
	**All persons**	**82.4**	82.4 - 82.4	**65.1**	65.0 - 65.2	**62.7**	62.6 - 62.9	**60.3**	60.1 - 60.5
	**Men**								
	**Women**	**82.4**	82.4 - 82.4	**65.1**	65.0 - 65.2	**62.7**	62.6 - 62.9	**60.3**	60.1 - 60.5
**Colon**									
	**All persons**	**67.0**	66.9 - 67.0	**48.2**	48.0 - 48.3	**46.1**	45.8 - 46.3	**44.9**	44.6 - 45.2
	**Men**	**68.6**	68.5 - 68.6	**48.7**	48.5 - 49.0	**46.3**	46.0 - 46.6	**44.8**	44.3 - 45.2
	**Women**	**65.4**	65.3 - 65.5	**47.6**	47.3 - 47.8	**45.8**	45.5 - 46.2	**44.9**	44.5 - 45.3
**Hodgkin's lymphoma**									
	**All persons**	**89.4**	89.4 - 89.4	**79.9**	79.9 - 80.0	**77.9**	77.9 - 78.0	**75.4**	75.4 - 75.5
	**Men**	**89.0**	89.0 - 89.0	**79.8**	79.8 - 79.9	**77.7**	77.6 - 77.8	**75.0**	74.9 - 75.1
	**Women**	**90.0**	90.0 - 90.0	**80.1**	80.0 - 80.1	**78.2**	78.1 - 78.3	**76.0**	75.9 - 76.1
**Kidney**									
	**All persons**	**63.0**	62.9 - 63.1	**44.9**	44.6 - 45.2	**41.1**	40.8 - 41.5	**37.2**	36.7 - 37.8
	**Men**	**64.1**	64.0 - 64.2	**45.5**	45.1 - 45.8	**41.6**	41.1 - 42.1	**37.6**	36.9 - 38.2
	**Women**	**61.1**	60.9 - 61.2	**43.8**	43.3 - 44.3	**40.3**	39.7 - 41.0	**36.7**	35.8 - 37.5
**Larynx**									
	**All persons**	**83.5**	83.5 - 83.6	**64.5**	64.3 - 64.7	**60.6**	60.3 - 60.9	**54.7**	54.2 - 55.1
	**Men**	**83.5**	83.5 - 83.6	**64.5**	64.3 - 64.7	**60.6**	60.3 - 60.9	**54.7**	54.2 - 55.1
	**Women**								
**Leukaemia**									
	**All persons**	**62.0**	61.9 - 62.1	**40.3**	39.9 - 40.7	**35.8**	35.3 - 36.3	**31.3**	30.6 - 31.9
	**Men**	**63.9**	63.8 - 64.0	**41.2**	40.7 - 41.6	**36.4**	35.8 - 36.9	**31.6**	30.9 - 32.4
	**Women**	**59.7**	59.5 - 59.9	**39.2**	38.5 - 39.9	**35.0**	34.1 - 35.9	**30.8**	29.6 - 32.0
**Lung**									
	**All persons**	**24.1**	23.8 - 24.4	**6.7**	6.1 - 7.3	**5.2**	4.6 - 5.9	**4.0**	3.3 - 4.7
	**Men**	**23.5**	23.1 - 23.9	**6.4**	5.6 - 7.2	**4.8**	4.0 - 5.8	**3.6**	2.7 - 4.7
	**Women**	**25.2**	24.7 - 25.6	**7.2**	6.4 - 8.1	**5.8**	4.9 - 6.7	**4.7**	3.8 - 5.7
**Melanoma**									
	**All persons**	**94.9**	94.9 - 94.9	**83.2**	83.2 - 83.2	**81.1**	81.1 - 81.1	**79.8**	79.7 - 79.8
	**Men**	**93.5**	93.5 - 93.5	**78.0**	77.9 - 78.0	**75.4**	75.3 - 75.4	**73.8**	73.8 - 73.9
	**Women**	**96.0**	96.0 - 96.0	**87.1**	87.1 - 87.1	**85.3**	85.3 - 85.3	**84.1**	84.1 - 84.2
**Myeloma**									
	**All persons**	**64.4**	64.3 - 64.5	**28.7**	27.8 - 29.6	**21.4**	20.1 - 22.7	**14.9**	13.0 - 16.9
	**Men**	**65.6**	65.4 - 65.7	**29.8**	28.7 - 30.9	**22.3**	20.7 - 24.0	**15.6**	13.2 - 18.2
	**Women**	**63.1**	62.9 - 63.3	**27.4**	26.0 - 28.8	**20.4**	18.4 - 22.4	**14.1**	11.2 - 17.3
**Non-Hodgkin lymphoma**									
	**All persons**	**70.8**	70.7 - 70.8	**53.3**	53.2 - 53.5	**49.1**	48.9 - 49.3	**44.3**	44.0 - 44.6
	**Men**	**70.7**	70.6 - 70.7	**52.6**	52.4 - 52.9	**48.4**	48.1 - 48.7	**43.7**	43.3 - 44.2
	**Women**	**70.8**	70.8 - 70.9	**54.1**	53.9 - 54.4	**49.9**	49.6 - 50.2	**44.9**	44.5 - 45.4
**Oesophagus**									
	**All persons**	**31.7**	31.2 - 32.2	**8.7**	7.3 - 10.1	**7.4**	5.9 - 9.1	**6.7**	5.0 - 8.7
	**Men**	**33.1**	32.5 - 33.6	**8.9**	7.3 - 10.6	**7.6**	5.9 - 9.7	**6.9**	4.9 - 9.3
	**Women**	**29.6**	28.6 - 30.5	**8.3**	6.1 - 11.0	**7.1**	4.7 - 10.2	**6.4**	3.7 - 10.1
**Others**									
	**All persons**	**54.8**	54.7 - 54.9	**35.8**	35.6 - 36.1	**33.2**	32.9 - 33.5	**30.8**	30.4 - 31.2
	**Men**	**53.7**	53.5 - 53.8	**33.1**	32.7 - 33.4	**30.4**	29.9 - 30.8	**28.1**	27.5 - 28.6
	**Women**	**56.1**	56.0 - 56.2	**39.0**	38.7 - 39.3	**36.3**	35.9 - 36.7	**33.9**	33.4 - 34.4
**Ovary**									
	**All persons**	**64.5**	64.4 - 64.5	**38.4**	38.0 - 38.8	**35.0**	34.5 - 35.5	**32.4**	31.7 - 33.0
	**Men**								
	**Women**	**64.5**	64.4 - 64.5	**38.4**	38.0 - 38.8	**35.0**	34.5 - 35.5	**32.4**	31.7 - 33.0
**Pancreas**									
	**All persons**	**13.7**	12.7 - 14.8	**3.1**	1.7 - 5.3	**2.5**	1.0 - 5.1	**2.0**	0.5 - 5.3
	**Men**	**14.4**	13.0 - 15.8	**3.4**	1.5 - 6.5	**2.6**	0.8 - 6.5	**2.1**	0.4 - 7.1
	**Women**	**13.1**	11.6 - 14.7	**2.9**	1.1 - 6.4	**2.3**	0.5 - 6.7	**1.9**	0.2 - 7.7

Appendix Table 2. Standardised net survival at 1, 5, 7, and 10 years for all cancers combined (CSI) and for 22 selected cancers, by sex: adults diagnosed in England and Wales during 2000-01

		1 year		5 year		7 year		10 year	
		NS	95% CI	NS	95% CI	NS	95% CI	NS	95% CI
**Prostate**									
	**All persons**	**88.0**	88.0 - 88.0	**70.9**	70.9 - 71.0	**65.3**	65.2 - 65.4	**60.1**	60.0 - 60.3
	**Men**	**88.0**	88.0 - 88.0	**70.9**	70.9 - 71.0	**65.3**	65.2 - 65.4	**60.1**	60.0 - 60.3
	**Women**								
**Rectum**									
	**All persons**	**74.4**	74.4 - 74.4	**50.1**	49.9 - 50.2	**46.6**	46.3 - 46.8	**44.3**	43.9 - 44.6
	**Men**	**75.0**	75.0 - 75.1	**49.7**	49.5 - 50.0	**46.0**	45.7 - 46.4	**43.5**	43.0 - 44.0
	**Women**	**73.5**	73.4 - 73.5	**50.5**	50.2 - 50.8	**47.4**	47.0 - 47.8	**45.4**	44.8 - 46.0
**Stomach**									
	**All persons**	**33.9**	33.5 - 34.4	**13.9**	12.7 - 15.0	**12.2**	10.9 - 13.6	**11.0**	9.5 - 12.6
	**Men**	**34.9**	34.4 - 35.4	**13.8**	12.4 - 15.2	**12.1**	10.5 - 13.8	**10.9**	9.0 - 12.9
	**Women**	**32.2**	31.4 - 33.1	**14.0**	12.1 - 16.0	**12.4**	10.3 - 14.8	**11.3**	8.8 - 14.1
**Testis**									
	**All persons**	**97.7**	97.7 - 97.7	**95.7**	95.7 - 95.7	**95.4**	95.4 - 95.4	**95.1**	95.1 - 95.1
	**Men**	**97.7**	97.7 - 97.7	**95.7**	95.7 - 95.7	**95.4**	95.4 - 95.4	**95.1**	95.1 - 95.1
	**Women**								
**Uterus**									
	**All persons**	**86.4**	86.4 - 86.4	**72.3**	72.3 - 72.4	**70.3**	70.3 - 70.4	**68.4**	68.3 - 68.5
	**Men**								
	**Women**	**86.4**	86.4 - 86.4	**72.3**	72.3 - 72.4	**70.3**	70.3 - 70.4	**68.4**	68.3 - 68.5

Appendix Table 2. Standardised net survival at 1, 5, 7, and 10 years for all cancers combined (CSI) and for 22 selected cancers, by sex: adults diagnosed in England and Wales during 2005-06

		1 year		5 year		7 year		10 year	
		NS	95% CI	NS	95% CI	NS	95% CI	NS	95% CI
All cancers combined (cancer survival index)									
	All persons	67.7	67.7 - 67.7	50.9	50.8 - 50.9	48.0	47.9 - 48.0	45.2	45.2 - 45.3
	Men	63.8	63.8 - 63.8	45.7	45.6 - 45.8	42.7	42.6 - 42.8	40.0	39.8 - 40.2
	Women	71.6	71.6 - 71.6	56.0	55.9 - 56.0	53.2	53.1 - 53.3	50.4	50.3 - 50.5
Bladder									
	All persons	74.0	73.9 - 74.0	54.5	54.3 - 54.7	51.3	51.1 - 51.6	47.9	47.5 - 48.2
	Men	77.9	77.9 - 78.0	58.0	57.8 - 58.2	54.8	54.5 - 55.0	51.2	50.9 - 51.6
	Women	63.7	63.6 - 63.9	45.5	44.9 - 46.0	42.4	41.7 - 43.1	39.1	38.2 - 40.0
Brain									
	All persons	36.9	36.5 - 37.3	17.9	16.8 - 19.1	15.7	14.4 - 17.1	13.8	12.2 - 15.5
	Men	37.3	36.8 - 37.9	17.0	15.5 - 18.7	14.9	13.0 - 16.8	13.0	10.8 - 15.4
	Women	36.3	35.7 - 37.0	19.1	17.5 - 20.8	16.9	15.0 - 18.9	14.9	12.6 - 17.3
Breast									
	All persons	94.7	94.7 - 94.7	84.5	84.5 - 84.5	81.1	81.1 - 81.1	76.9	76.9 - 76.9
	Men								
	Women	94.7	94.7 - 94.7	84.5	84.5 - 84.5	81.1	81.1 - 81.1	76.9	76.9 - 76.9
Cervix									
	All persons	83.0	83.0 - 83.0	66.6	66.5 - 66.7	64.3	64.2 - 64.4	61.8	61.7 - 62.0
	Men								
	Women	83.0	83.0 - 83.0	66.6	66.5 - 66.7	64.3	64.2 - 64.4	61.8	61.7 - 62.0
Colon									
	All persons	70.2	70.2 - 70.2	52.3	52.1 - 52.4	50.3	50.1 - 50.5	49.2	49.0 - 49.5
	Men	71.9	71.9 - 72.0	53.1	52.9 - 53.3	50.8	50.5 - 51.1	49.3	48.9 - 49.6
	Women	68.5	68.4 - 68.5	51.4	51.2 - 51.7	49.9	49.6 - 50.1	49.1	48.8 - 49.5
Hodgkin's lymphoma									
	All persons	90.7	90.7 - 90.7	82.7	82.6 - 82.7	80.8	80.8 - 80.9	78.4	78.3 - 78.4
	Men	90.3	90.3 - 90.3	82.5	82.5 - 82.6	80.6	80.5 - 80.6	77.9	77.8 - 77.9
	Women	91.4	91.4 - 91.4	82.8	82.8 - 82.9	81.1	81.1 - 81.2	79.1	79.0 - 79.1
Kidney									
	All persons	68.4	68.3 - 68.4	50.6	50.3 - 50.8	46.8	46.5 - 47.1	42.8	42.4 - 43.3
	Men	69.1	69.1 - 69.2	50.7	50.4 - 51.0	46.9	46.4 - 47.3	42.8	42.2 - 43.3
	Women	67.1	67.0 - 67.2	50.3	49.9 - 50.7	46.7	46.2 - 47.3	43.0	42.3 - 43.7
Larynx									
	All persons	84.3	84.2 - 84.3	65.1	65.0 - 65.3	60.8	60.5 - 61.1	54.1	53.6 - 54.5
	Men	84.3	84.2 - 84.3	65.1	65.0 - 65.3	60.8	60.5 - 61.1	54.1	53.6 - 54.5
	Women								
Leukaemia									
	All persons	66.0	65.9 - 66.1	45.4	45.1 - 45.7	41.1	40.6 - 41.5	36.7	36.2 - 37.3
	Men	68.1	68.0 - 68.2	46.6	46.2 - 46.9	41.9	41.4 - 42.4	37.3	36.6 - 37.9
	Women	63.3	63.1 - 63.4	43.9	43.3 - 44.4	40.0	39.2 - 40.7	36.1	35.0 - 37.1
Lung									
	All persons	28.1	27.8 - 28.4	8.4	7.8 - 9.0	6.5	5.9 - 7.2	5.0	4.4 - 5.8
	Men	27.1	26.7 - 27.4	7.8	7.1 - 8.6	5.9	5.1 - 6.8	4.4	3.5 - 5.4
	Women	29.9	29.5 - 30.3	9.5	8.6 - 10.4	7.6	6.7 - 8.6	6.2	5.2 - 7.3
Melanoma									
	All persons	96.1	96.1 - 96.1	86.6	86.5 - 86.6	84.9	84.9 - 84.9	84.0	84.0 - 84.0
	Men	95.0	95.0 - 95.0	82.3	82.3 - 82.4	80.3	80.3 - 80.3	79.2	79.2 - 79.3
	Women	97.0	97.0 - 97.0	89.7	89.7 - 89.7	88.3	88.3 - 88.3	87.5	87.5 - 87.5
Myeloma									
	All persons	70.2	70.1 - 70.3	35.6	34.9 - 36.3	27.6	26.5 - 28.7	19.9	18.2 - 21.7
	Men	71.5	71.4 - 71.6	37.4	36.5 - 38.2	29.1	27.8 - 30.5	21.2	18.9 - 23.5
	Women	68.7	68.6 - 68.9	33.6	32.5 - 34.8	25.8	24.1 - 27.6	18.5	15.9 - 21.3
Non-Hodgkin lymphoma									
	All persons	74.6	74.5 - 74.6	59.8	59.7 - 60.0	56.1	55.9 - 56.2	51.7	51.5 - 52.0
	Men	74.6	74.5 - 74.6	59.1	59.0 - 59.3	55.4	55.1 - 55.6	51.1	50.8 - 51.5
	Women	74.6	74.5 - 74.6	60.6	60.5 - 60.8	56.9	56.6 - 57.1	52.4	52.0 - 52.7
Oesophagus									
	All persons	36.8	36.4 - 37.2	11.2	9.9 - 12.7	9.7	8.2 - 11.4	8.8	7.1 - 10.8
	Men	38.9	38.4 - 39.3	11.9	10.3 - 13.6	10.3	8.4 - 12.3	9.3	7.2 - 11.8
	Women	33.6	32.7 - 34.5	10.2	7.9 - 12.9	8.9	6.3 - 11.9	8.1	5.2 - 11.7
Others									
	All persons	58.3	58.2 - 58.4	39.0	38.8 - 39.2	36.3	36.0 - 36.6	33.9	33.5 - 34.2
	Men	57.5	57.4 - 57.6	36.5	36.2 - 36.8	33.7	33.3 - 34.1	31.3	30.8 - 31.9
	Women	59.2	59.1 - 59.3	41.8	41.6 - 42.1	39.2	38.8 - 39.6	36.7	36.3 - 37.2
Ovary									
	All persons	68.8	68.7 - 68.8	43.2	42.9 - 43.5	39.3	38.9 - 39.7	35.9	35.4 - 36.5
	Men								
	Women	68.8	68.7 - 68.8	43.2	42.9 - 43.5	39.3	38.9 - 39.7	35.9	35.4 - 36.5
Pancreas									
	All persons	16.1	15.1 - 17.1	3.9	2.3 - 6.1	3.1	1.4 - 5.8	2.5	0.8 - 5.9
	Men	16.7	15.4 - 18.1	4.1	2.0 - 7.3	3.2	1.1 - 7.1	2.6	0.6 - 7.6
	Women	15.4	13.9 - 17.0	3.7	1.6 - 7.2	3.0	0.9 - 7.3	2.4	0.4 - 8.1

Appendix Table 2. Standardised net survival at 1, 5, 7, and 10 years for all cancers combined (CSI) and for 22 selected cancers, by sex: adults diagnosed in England and Wales during 2005-06

		1 year		5 year		7 year		10 year	
		NS	95% CI	NS	95% CI	NS	95% CI	NS	95% CI
**Prostate**									
	**All persons**	**92.7**	92.7 - 92.7	**80.9**	80.9 - 80.9	**76.8**	76.8 - 76.8	**72.8**	72.8 - 72.9
	**Men**	**92.7**	92.7 - 92.7	**80.9**	80.9 - 80.9	**76.8**	76.8 - 76.8	**72.8**	72.8 - 72.9
	**Women**								
**Rectum**									
	**All persons**	**77.7**	77.7 - 77.8	**54.8**	54.7 - 55.0	**51.5**	51.3 - 51.7	**49.3**	49.0 - 49.6
	**Men**	**78.5**	78.5 - 78.5	**54.8**	54.6 - 55.0	**51.2**	51.0 - 51.5	**48.7**	48.3 - 49.1
	**Women**	**76.7**	76.6 - 76.7	**54.9**	54.6 - 55.1	**51.9**	51.6 - 52.3	**50.1**	49.6 - 50.6
**Stomach**									
	**All persons**	**37.7**	37.3 - 38.0	**15.9**	14.9 - 17.0	**14.0**	12.8 - 15.3	**12.6**	11.2 - 14.1
	**Men**	**38.9**	38.5 - 39.3	**15.9**	14.6 - 17.2	**13.9**	12.4 - 15.4	**12.4**	10.6 - 14.3
	**Women**	**35.4**	34.7 - 36.1	**16.0**	14.2 - 17.8	**14.2**	12.2 - 16.4	**12.9**	10.6 - 15.5
**Testis**									
	**All persons**	**98.1**	98.1 - 98.1	**96.5**	96.5 - 96.5	**96.2**	96.2 - 96.2	**96.0**	96.0 - 96.0
	**Men**	**98.1**	98.1 - 98.1	**96.5**	96.5 - 96.5	**96.2**	96.2 - 96.2	**96.0**	96.0 - 96.0
	**Women**								
**Uterus**									
	**All persons**	**88.6**	88.6 - 88.6	**75.0**	75.0 - 75.1	**73.0**	73.0 - 73.1	**71.2**	71.1 - 71.3
	**Men**								
	**Women**	**88.6**	88.6 - 88.6	**75.0**	75.0 - 75.1	**73.0**	73.0 - 73.1	**71.2**	71.1 - 71.3

Appendix Table 2. Standardised net survival at 1, 5, 7, and 10 years for all cancers combined (CSI) and for 22 selected cancers, by sex: adults diagnosed in England and Wales during 2010-11

		1 year		5 year		7 year		10 year	
		**NS**	**95% CI**	**NS**	**95% CI**	**NS**	**95% CI**	**NS**	**95% CI**
**All cancers combined (cancer survival index)**									
	**All persons**	**70.4**	70.4 - 70.4	**53.7**	53.6 - 53.7	**50.8**	50.7 - 50.8	**47.9**	47.8 - 47.9
	**Men**	**66.8**	66.8 - 66.8	**48.7**	48.6 - 48.8	**45.8**	45.7 - 45.9	**43.1**	42.9 - 43.3
	**Women**	**74.0**	74.0 - 74.0	**58.5**	58.5 - 58.5	**55.6**	55.6 - 55.7	**52.6**	52.5 - 52.7
**Bladder**									
	**All persons**	**73.1**	73.0 - 73.1	**52.4**	52.2 - 52.6	**49.2**	48.9 - 49.5	**45.7**	45.3 - 46.2
	**Men**	**77.2**	77.2 - 77.3	**56.1**	55.9 - 56.3	**52.8**	52.5 - 53.1	**49.3**	48.8 - 49.8
	**Women**	**62.3**	62.1 - 62.5	**42.9**	42.2 - 43.5	**39.8**	39.0 - 40.6	**36.6**	35.4 - 37.7
**Brain**									
	**All persons**	**41.2**	40.9 - 41.5	**20.7**	19.7 - 21.7	**18.3**	17.0 - 19.5	**16.0**	14.4 - 17.7
	**Men**	**41.9**	41.5 - 42.3	**19.9**	18.5 - 21.4	**17.5**	15.8 - 19.3	**15.3**	13.1 - 17.7
	**Women**	**40.3**	39.8 - 40.8	**21.7**	20.3 - 23.2	**19.3**	17.6 - 21.1	**17.1**	14.8 - 19.4
**Breast**									
	**All persons**	**95.8**	95.8 - 95.8	**86.6**	86.6 - 86.6	**83.1**	83.1 - 83.1	**78.3**	78.3 - 78.3
	**Men**								
	**Women**	**95.8**	95.8 - 95.8	**86.6**	86.6 - 86.6	**83.1**	83.1 - 83.1	**78.3**	78.3 - 78.3
**Cervix**									
	**All persons**	**83.7**	83.7 - 83.7	**68.0**	67.9 - 68.1	**65.6**	65.5 - 65.7	**62.9**	62.7 - 63.1
	**Men**								
	**Women**	**83.7**	83.7 - 83.7	**68.0**	67.9 - 68.1	**65.6**	65.5 - 65.7	**62.9**	62.7 - 63.1
**Colon**									
	**All persons**	**72.3**	72.3 - 72.3	**54.8**	54.7 - 54.9	**53.0**	52.8 - 53.1	**52.1**	51.8 - 52.3
	**Men**	**74.3**	74.3 - 74.4	**55.9**	55.8 - 56.1	**53.7**	53.4 - 53.9	**52.2**	51.9 - 52.6
	**Women**	**70.3**	70.3 - 70.4	**53.6**	53.4 - 53.8	**52.3**	52.0 - 52.5	**51.9**	51.6 - 52.2
**Hodgkin's lymphoma**									
	**All persons**	**91.9**	91.9 - 91.9	**84.9**	84.9 - 84.9	**83.0**	82.9 - 83.0	**80.2**	80.2 - 80.2
	**Men**	**91.6**	91.6 - 91.6	**84.7**	84.6 - 84.7	**82.6**	82.5 - 82.6	**79.4**	79.3 - 79.5
	**Women**	**92.3**	92.3 - 92.3	**85.2**	85.2 - 85.3	**83.5**	83.5 - 83.6	**81.2**	81.2 - 81.3
**Kidney**									
	**All persons**	**73.6**	73.6 - 73.7	**56.4**	56.2 - 56.5	**52.5**	52.2 - 52.7	**48.3**	47.9 - 48.6
	**Men**	**74.2**	74.2 - 74.2	**56.3**	56.1 - 56.5	**52.3**	51.9 - 52.6	**47.9**	47.4 - 48.4
	**Women**	**72.7**	72.6 - 72.7	**56.5**	56.2 - 56.7	**52.8**	52.5 - 53.2	**48.9**	48.3 - 49.4
**Larynx**									
	**All persons**	**84.5**	84.5 - 84.5	**64.5**	64.3 - 64.7	**59.4**	59.1 - 59.8	**51.6**	50.9 - 52.2
	**Men**	**84.5**	84.5 - 84.5	**64.5**	64.3 - 64.7	**59.4**	59.1 - 59.8	**51.6**	50.9 - 52.2
	**Women**								
**Leukaemia**									
	**All persons**	**69.4**	69.3 - 69.5	**50.0**	49.8 - 50.3	**46.0**	45.6 - 46.3	**41.9**	41.4 - 42.4
	**Men**	**71.6**	71.5 - 71.6	**51.2**	51.0 - 51.5	**46.8**	46.4 - 47.2	**42.3**	41.8 - 42.8
	**Women**	**66.6**	66.5 - 66.7	**48.5**	48.0 - 48.9	**44.9**	44.3 - 45.5	**41.3**	40.4 - 42.2
**Lung**									
	**All persons**	**33.2**	33.0 - 33.4	**11.1**	10.6 - 11.7	**8.7**	8.1 - 9.4	**6.8**	6.0 - 7.6
	**Men**	**31.5**	31.2 - 31.8	**10.0**	9.2 - 10.8	**7.6**	6.7 - 8.5	**5.6**	4.6 - 6.7
	**Women**	**36.2**	35.9 - 36.5	**13.1**	12.3 - 13.9	**10.7**	9.8 - 11.7	**8.8**	7.8 - 10.0
**Melanoma**									
	**All persons**	**97.2**	97.2 - 97.2	**89.5**	89.5 - 89.5	**88.3**	88.3 - 88.3	**87.8**	87.8 - 87.8
	**Men**	**96.3**	96.3 - 96.3	**86.3**	86.2 - 86.3	**84.7**	84.7 - 84.8	**84.0**	84.0 - 84.1
	**Women**	**97.9**	97.9 - 97.9	**92.0**	91.9 - 92.0	**90.9**	90.9 - 90.9	**90.6**	90.6 - 90.6
**Myeloma**									
	**All persons**	**76.0**	75.9 - 76.0	**43.7**	43.2 - 44.2	**35.1**	34.2 - 35.9	**26.3**	24.8 - 27.9
	**Men**	**77.5**	77.5 - 77.6	**45.7**	45.1 - 46.2	**36.9**	35.8 - 37.9	**27.8**	25.9 - 29.8
	**Women**	**74.3**	74.2 - 74.4	**41.5**	40.7 - 42.3	**33.1**	31.7 - 34.5	**24.7**	22.2 - 27.2
**Non-Hodgkin lymphoma**									
	**All persons**	**77.9**	77.9 - 78.0	**65.2**	65.2 - 65.3	**61.9**	61.8 - 62.0	**57.9**	57.7 - 58.1
	**Men**	**78.0**	77.9 - 78.0	**64.5**	64.4 - 64.6	**61.1**	60.9 - 61.2	**57.1**	56.9 - 57.4
	**Women**	**77.9**	77.9 - 78.0	**66.1**	65.9 - 66.2	**62.8**	62.6 - 62.9	**58.8**	58.5 - 59.1
**Oesophagus**									
	**All persons**	**40.8**	40.4 - 41.1	**13.6**	12.4 - 14.9	**11.9**	10.4 - 13.4	**10.8**	9.0 - 12.6
	**Men**	**43.3**	42.9 - 43.6	**14.4**	12.9 - 15.9	**12.4**	10.7 - 14.3	**11.1**	9.1 - 13.4
	**Women**	**36.8**	36.1 - 37.6	**12.5**	10.4 - 14.8	**11.0**	8.6 - 13.8	**10.1**	7.3 - 13.6
**Others**									
	**All persons**	**62.4**	62.4 - 62.5	**42.7**	42.6 - 42.9	**40.0**	39.7 - 40.2	**37.5**	37.2 - 37.8
	**Men**	**62.1**	62.0 - 62.2	**40.6**	40.3 - 40.8	**37.7**	37.3 - 38.0	**35.2**	34.7 - 35.6
	**Women**	**62.8**	62.7 - 62.8	**45.2**	45.0 - 45.5	**42.6**	42.3 - 42.9	**40.1**	39.7 - 40.6
**Ovary**									
	**All persons**	**72.6**	72.6 - 72.7	**47.1**	46.8 - 47.3	**42.3**	41.9 - 42.7	**37.9**	37.3 - 38.4
	**Men**								
	**Women**	**72.6**	72.6 - 72.7	**47.1**	46.8 - 47.3	**42.3**	41.9 - 42.7	**37.9**	37.3 - 38.4
**Pancreas**									
	**All persons**	**19.0**	18.2 - 19.9	**4.9**	3.3 - 7.0	**3.9**	2.1 - 6.4	**3.1**	1.3 - 6.2
	**Men**	**19.8**	18.7 - 21.0	**5.1**	3.0 - 8.0	**4.0**	1.8 - 7.6	**3.1**	0.9 - 7.8
	**Women**	**18.3**	17.0 - 19.6	**4.7**	2.6 - 7.9	**3.8**	1.5 - 7.7	**3.1**	0.8 - 8.2

Appendix Table 2. Standardised net survival at 1, 5, 7, and 10 years for all cancers combined (CSI) and for 22 selected cancers, by sex: adults diagnosed in England and Wales during 2010-11

		1 year		5 year		7 year		10 year	
		NS	95% CI	NS	95% CI	NS	95% CI	NS	95% CI
**Prostate**									
	**All persons**	**95.0**	95.0 - 95.0	**86.1**	86.1 - 86.1	**82.8**	82.8 - 82.8	**79.7**	79.6 - 79.7
	**Men**	**95.0**	95.0 - 95.0	**86.1**	86.1 - 86.1	**82.8**	82.8 - 82.8	**79.7**	79.6 - 79.7
	**Women**								
**Rectum**									
	**All persons**	**80.1**	80.0 - 80.1	**57.7**	57.5 - 57.8	**54.3**	54.2 - 54.5	**52.1**	51.9 - 52.4
	**Men**	**81.0**	81.0 - 81.0	**57.9**	57.8 - 58.1	**54.3**	54.1 - 54.5	**51.8**	51.4 - 52.2
	**Women**	**78.7**	78.6 - 78.7	**57.3**	57.1 - 57.5	**54.4**	54.1 - 54.7	**52.7**	52.2 - 53.1
**Stomach**									
	**All persons**	**40.8**	40.5 - 41.2	**17.8**	16.7 - 19.0	**15.7**	14.3 - 17.0	**14.0**	12.3 - 15.7
	**Men**	**42.3**	41.9 - 42.6	**17.8**	16.4 - 19.2	**15.4**	13.8 - 17.2	**13.6**	11.6 - 15.8
	**Women**	**38.2**	37.5 - 38.9	**17.9**	16.0 - 19.9	**16.0**	13.8 - 18.4	**14.6**	11.9 - 17.5
**Testis**									
	**All persons**	**98.3**	98.3 - 98.3	**96.9**	96.9 - 96.9	**96.6**	96.6 - 96.6	**96.4**	96.4 - 96.4
	**Men**	**98.3**	98.3 - 98.3	**96.9**	96.9 - 96.9	**96.6**	96.6 - 96.6	**96.4**	96.4 - 96.4
	**Women**								
**Uterus**									
	**All persons**	**89.7**	89.7 - 89.7	**76.4**	76.3 - 76.4	**74.3**	74.3 - 74.4	**72.4**	72.3 - 72.4
	**Men**								
	**Women**	**89.7**	89.7 - 89.7	**76.4**	76.3 - 76.4	**74.3**	74.3 - 74.4	**72.4**	72.3 - 72.4

Appendix Table 2. Standardised net survival at 1, 5, 7, and 10 years for all cancers combined (CSI) and for 22 selected cancers, by sex: adults diagnosed in England and Wales during 2015-16

		1 year		5 year		7 year		10 year	
		NS	95% CI	NS	95% CI	NS	95% CI	NS	95% CI
**All cancers combined (cancer survival index)**									
	**All persons**	**72.9**	72.9 - 72.9	**55.7**	55.7 - 55.8	**52.6**	52.5 - 52.6	**49.3**	49.2 - 49.4
	**Men**	**69.4**	69.4 - 69.4	**50.7**	50.6 - 50.8	**47.5**	47.4 - 47.7	**44.6**	44.4 - 44.9
	**Women**	**76.3**	76.3 - 76.3	**60.6**	60.6 - 60.7	**57.5**	57.4 - 57.6	**53.9**	53.8 - 54.1
**Bladder**									
	**All persons**	**72.2**	72.1 - 72.2	**50.3**	50.0 - 50.7	**47.0**	46.5 - 47.5	**43.5**	42.7 - 44.3
	**Men**	**76.6**	76.5 - 76.6	**54.2**	53.9 - 54.6	**50.8**	50.3 - 51.3	**47.1**	46.3 - 48.0
	**Women**	**60.9**	60.6 - 61.1	**40.3**	39.3 - 41.3	**37.2**	36.0 - 38.5	**34.0**	32.4 - 35.7
**Brain**									
	**All persons**	**46.1**	45.8 - 46.4	**23.6**	22.5 - 24.8	**20.8**	19.3 - 22.4	**18.2**	16.0 - 20.5
	**Men**	**46.8**	46.4 - 47.2	**22.6**	20.9 - 24.3	**19.7**	17.4 - 22.0	**17.1**	14.0 - 20.4
	**Women**	**45.2**	44.8 - 45.7	**25.1**	23.6 - 26.7	**22.4**	20.3 - 24.6	**19.8**	16.9 - 22.8
**Breast**									
	**All persons**	**96.3**	96.3 - 96.3	**87.3**	87.3 - 87.3	**83.4**	83.4 - 83.4	**77.4**	77.4 - 77.4
	**Men**								
	**Women**	**96.3**	96.3 - 96.3	**87.3**	87.3 - 87.3	**83.4**	83.4 - 83.4	**77.4**	77.4 - 77.4
**Cervix**									
	**All persons**	**84.5**	84.5 - 84.5	**69.2**	69.1 - 69.3	**66.7**	66.5 - 66.8	**63.7**	63.5 - 63.9
	**Men**								
	**Women**	**84.5**	84.5 - 84.5	**69.2**	69.1 - 69.3	**66.7**	66.5 - 66.8	**63.7**	63.5 - 63.9
**Colon**									
	**All persons**	**73.7**	73.6 - 73.7	**55.9**	55.8 - 56.1	**54.3**	54.1 - 54.5	**53.6**	53.3 - 53.9
	**Men**	**76.0**	76.0 - 76.0	**57.3**	57.1 - 57.5	**55.1**	54.8 - 55.4	**53.7**	53.3 - 54.2
	**Women**	**71.4**	71.3 - 71.4	**54.5**	54.3 - 54.8	**53.5**	53.2 - 53.8	**53.5**	53.1 - 53.9
**Hodgkin's lymphoma**									
	**All persons**	**92.8**	92.8 - 92.8	**86.7**	86.7 - 86.7	**84.5**	84.5 - 84.5	**81.2**	81.1 - 81.2
	**Men**	**92.7**	92.7 - 92.7	**86.2**	86.1 - 86.2	**83.7**	83.7 - 83.7	**79.8**	79.7 - 79.9
	**Women**	**92.9**	92.9 - 92.9	**87.4**	87.4 - 87.4	**85.6**	85.6 - 85.6	**82.9**	82.9 - 83.0
**Kidney**									
	**All persons**	**78.9**	78.8 - 78.9	**62.3**	62.2 - 62.5	**58.1**	57.9 - 58.4	**53.4**	53.0 - 53.9
	**Men**	**79.4**	79.4 - 79.4	**62.2**	62.1 - 62.4	**57.9**	57.6 - 58.2	**53.0**	52.4 - 53.6
	**Women**	**78.0**	77.9 - 78.0	**62.5**	62.3 - 62.7	**58.6**	58.2 - 59.0	**54.2**	53.5 - 54.9
**Larynx**									
	**All persons**	**84.4**	84.4 - 84.4	**62.9**	62.6 - 63.2	**57.0**	56.4 - 57.6	**47.7**	46.2 - 49.1
	**Men**	**84.4**	84.4 - 84.4	**62.9**	62.6 - 63.2	**57.0**	56.4 - 57.6	**47.7**	46.2 - 49.1
	**Women**								
**Leukaemia**									
	**All persons**	**72.5**	72.5 - 72.6	**54.4**	54.2 - 54.7	**50.6**	50.3 - 51.0	**46.8**	46.3 - 47.3
	**Men**	**74.6**	74.5 - 74.6	**55.4**	55.1 - 55.7	**51.2**	50.8 - 51.6	**46.9**	46.4 - 47.5
	**Women**	**69.9**	69.8 - 70.0	**53.2**	52.7 - 53.7	**49.9**	49.3 - 50.6	**46.7**	45.7 - 47.6
**Lung**									
	**All persons**	**39.5**	39.3 - 39.7	**15.1**	14.4 - 15.8	**12.1**	11.2 - 12.9	**9.5**	8.5 - 10.6
	**Men**	**36.8**	36.5 - 37.1	**13.1**	12.0 - 14.1	**10.0**	8.7 - 11.4	**7.4**	5.8 - 9.2
	**Women**	**44.1**	43.9 - 44.4	**18.6**	17.8 - 19.4	**15.6**	14.6 - 16.6	**13.1**	11.9 - 14.3
**Melanoma**									
	**All persons**	**98.2**	98.2 - 98.2	**92.4**	92.4 - 92.4	**91.5**	91.5 - 91.5	**91.3**	91.3 - 91.3
	**Men**	**97.5**	97.5 - 97.5	**90.0**	90.0 - 90.0	**88.9**	88.9 - 88.9	**88.4**	88.4 - 88.4
	**Women**	**98.7**	98.7 - 98.7	**94.1**	94.1 - 94.1	**93.4**	93.4 - 93.4	**93.4**	93.4 - 93.4
**Myeloma**									
	**All persons**	**81.7**	81.7 - 81.8	**52.6**	52.2 - 53.1	**43.7**	42.8 - 44.6	**34.0**	32.0 - 36.0
	**Men**	**83.2**	83.2 - 83.2	**54.1**	53.6 - 54.6	**45.0**	43.9 - 46.1	**35.1**	32.5 - 37.7
	**Women**	**80.1**	80.1 - 80.2	**51.0**	50.3 - 51.7	**42.2**	40.7 - 43.7	**32.7**	29.7 - 35.9
**Non-Hodgkin lymphoma**									
	**All persons**	**81.0**	81.0 - 81.0	**69.5**	69.4 - 69.5	**66.3**	66.2 - 66.5	**62.6**	62.4 - 62.8
	**Men**	**81.1**	81.0 - 81.1	**68.8**	68.7 - 68.9	**65.4**	65.3 - 65.6	**61.5**	61.2 - 61.8
	**Women**	**80.9**	80.9 - 80.9	**70.3**	70.2 - 70.4	**67.4**	67.2 - 67.5	**63.8**	63.5 - 64.1
**Oesophagus**									
	**All persons**	**44.0**	43.7 - 44.4	**15.8**	14.2 - 17.5	**13.7**	11.6 - 15.9	**12.3**	9.7 - 15.3
	**Men**	**46.5**	46.2 - 46.9	**16.1**	14.2 - 18.2	**13.7**	11.2 - 16.5	**11.9**	8.7 - 15.7
	**Women**	**40.1**	39.3 - 40.9	**15.3**	12.5 - 18.4	**13.8**	10.4 - 17.6	**12.8**	8.7 - 17.9
**Others**									
	**All persons**	**67.1**	67.0 - 67.1	**47.2**	47.0 - 47.4	**44.3**	44.0 - 44.6	**41.7**	41.4 - 42.1
	**Men**	**67.2**	67.2 - 67.3	**45.3**	45.0 - 45.6	**42.2**	41.8 - 42.6	**39.5**	39.0 - 40.1
	**Women**	**66.9**	66.9 - 67.0	**49.3**	49.0 - 49.6	**46.7**	46.3 - 47.0	**44.3**	43.8 - 44.8
**Ovary**									
	**All persons**	**76.4**	76.3 - 76.4	**50.6**	50.3 - 50.9	**44.8**	44.3 - 45.3	**39.0**	38.2 - 39.8
	**Men**								
	**Women**	**76.4**	76.3 - 76.4	**50.6**	50.3 - 50.9	**44.8**	44.3 - 45.3	**39.0**	38.2 - 39.8
**Pancreas**									
	**All persons**	**22.8**	21.9 - 23.7	**6.4**	4.3 - 9.0	**5.0**	2.7 - 8.3	**3.9**	1.4 - 8.2
	**Men**	**23.8**	22.6 - 25.0	**6.5**	3.8 - 10.3	**5.0**	2.0 - 9.9	**3.8**	0.9 - 10.4
	**Women**	**21.9**	20.6 - 23.3	**6.2**	3.4 - 10.2	**5.0**	1.9 - 10.1	**4.0**	0.9 - 10.8

Appendix Table 2. Standardised net survival at 1, 5, 7, and 10 years for all cancers combined (CSI) and for 22 selected cancers, by sex: adults diagnosed in England and Wales during 2015-16

		1 year		5 year		7 year		10 year	
		NS	95% CI	NS	95% CI	NS	95% CI	NS	95% CI
**Prostate**									
	**All persons**	**95.2**	95.2 - 95.2	**86.3**	86.3 - 86.3	**83.1**	83.0 - 83.1	**79.8**	79.8 - 79.9
	**Men**	**95.2**	95.2 - 95.2	**86.3**	86.3 - 86.3	**83.1**	83.0 - 83.1	**79.8**	79.8 - 79.9
	**Women**								
**Rectum**									
	**All persons**	**81.7**	81.7 - 81.7	**59.1**	58.9 - 59.3	**55.7**	55.5 - 56.0	**53.5**	53.1 - 53.9
	**Men**	**83.0**	83.0 - 83.0	**59.6**	59.4 - 59.8	**55.9**	55.6 - 56.2	**53.3**	52.7 - 53.8
	**Women**	**79.9**	79.9 - 79.9	**58.4**	58.1 - 58.6	**55.5**	55.1 - 55.9	**53.7**	53.1 - 54.4
**Stomach**									
	**All persons**	**43.8**	43.4 - 44.2	**19.8**	18.3 - 21.3	**17.3**	15.5 - 19.3	**15.4**	13.0 - 17.8
	**Men**	**45.4**	44.9 - 45.8	**19.7**	17.9 - 21.6	**17.0**	14.7 - 19.4	**14.8**	11.9 - 18.0
	**Women**	**40.9**	40.0 - 41.7	**19.9**	17.5 - 22.5	**17.9**	14.9 - 21.2	**16.4**	12.7 - 20.4
**Testis**									
	**All persons**	**98.4**	98.4 - 98.4	**97.0**	97.0 - 97.0	**96.7**	96.7 - 96.7	**96.4**	96.4 - 96.5
	**Men**	**98.4**	98.4 - 98.4	**97.0**	97.0 - 97.0	**96.7**	96.7 - 96.7	**96.4**	96.4 - 96.5
	**Women**								
**Uterus**									
	**All persons**	**90.0**	90.0 - 90.0	**76.7**	76.7 - 76.8	**74.5**	74.4 - 74.6	**72.2**	72.1 - 72.3
	**Men**								
	**Women**	**90.0**	90.0 - 90.0	**76.7**	76.7 - 76.8	**74.5**	74.4 - 74.6	**72.2**	72.1 - 72.3

Appendix Table 2. Standardised net survival at 1, 5, 7, and 10 years for all cancers combined (CSI) and for 22 selected cancers, by sex: adults diagnosed in England and Wales during 2018

		1 year		5 year		7 year		10 year	
		**NS**	**95% CI**	**NS**	**95% CI**	**NS**	**95% CI**	**NS**	**95% CI**
**All cancers combined (cancer survival index)**									
	**All persons**	**74.0**	74.0 - 74.0	**56.6**	56.6 - 56.7	**53.3**	53.3 - 53.4	**49.8**	49.7 - 49.9
	**Men**	**70.6**	70.6 - 70.6	**51.5**	51.4 - 51.6	**48.2**	48.0 - 48.4	**45.1**	44.8 - 45.4
	**Women**	**77.4**	77.4 - 77.4	**61.7**	61.6 - 61.7	**58.4**	58.3 - 58.5	**54.4**	54.3 - 54.6
**Bladder**									
	**All persons**	**71.8**	71.7 - 71.8	**49.3**	48.8 - 49.7	**45.9**	45.2 - 46.5	**42.3**	41.2 - 43.4
	**Men**	**76.1**	76.1 - 76.2	**53.2**	52.7 - 53.6	**49.6**	49.0 - 50.3	**45.9**	44.7 - 47.1
	**Women**	**60.5**	60.1 - 60.8	**39.3**	38.0 - 40.5	**36.1**	34.6 - 37.7	**33.0**	31.0 - 35.0
**Brain**									
	**All persons**	**48.4**	48.1 - 48.8	**25.1**	23.7 - 26.5	**22.1**	20.2 - 24.0	**19.2**	16.6 - 22.0
	**Men**	**49.0**	48.6 - 49.4	**23.9**	21.9 - 25.9	**20.8**	18.1 - 23.6	**18.0**	14.2 - 22.1
	**Women**	**47.6**	47.1 - 48.1	**26.7**	24.9 - 28.6	**23.8**	21.3 - 26.4	**21.0**	17.5 - 24.7
**Breast**									
	**All persons**	**96.5**	96.5 - 96.5	**87.5**	87.5 - 87.5	**83.3**	83.3 - 83.3	**76.6**	76.5 - 76.6
	**Men**								
	**Women**	**96.5**	96.5 - 96.5	**87.5**	87.5 - 87.5	**83.3**	83.3 - 83.3	**76.6**	76.5 - 76.6
**Cervix**									
	**All persons**	**84.3**	84.3 - 84.3	**69.3**	69.1 - 69.4	**66.6**	66.5 - 66.8	**63.5**	63.3 - 63.8
	**Men**								
	**Women**	**84.3**	84.3 - 84.3	**69.3**	69.1 - 69.4	**66.6**	66.5 - 66.8	**63.5**	63.3 - 63.8
**Colon**									
	**All persons**	**74.5**	74.4 - 74.5	**56.4**	56.2 - 56.6	**54.9**	54.6 - 55.1	**54.3**	53.9 - 54.7
	**Men**	**76.8**	76.8 - 76.8	**57.8**	57.5 - 58.1	**55.5**	55.2 - 55.9	**54.2**	53.6 - 54.8
	**Women**	**72.1**	72.1 - 72.2	**55.1**	54.8 - 55.4	**54.2**	53.8 - 54.6	**54.4**	53.9 - 54.9
**Hodgkin's lymphoma**									
	**All persons**	**93.2**	93.2 - 93.2	**87.5**	87.5 - 87.5	**85.3**	85.2 - 85.3	**81.6**	81.6 - 81.7
	**Men**	**93.2**	93.1 - 93.2	**86.9**	86.8 - 86.9	**84.2**	84.2 - 84.3	**80.0**	79.9 - 80.1
	**Women**	**93.2**	93.2 - 93.2	**88.4**	88.4 - 88.4	**86.6**	86.6 - 86.6	**83.7**	83.7 - 83.8
**Kidney**									
	**All persons**	**81.1**	81.1 - 81.1	**65.0**	64.8 - 65.2	**60.6**	60.4 - 60.9	**55.6**	55.1 - 56.2
	**Men**	**81.7**	81.7 - 81.8	**65.1**	64.9 - 65.3	**60.5**	60.2 - 60.9	**55.3**	54.6 - 56.0
	**Women**	**80.1**	80.0 - 80.1	**64.9**	64.6 - 65.2	**60.8**	60.4 - 61.3	**56.2**	55.3 - 57.0
**Larynx**									
	**All persons**	**84.4**	84.3 - 84.4	**62.0**	61.6 - 62.4	**55.6**	54.8 - 56.4	**45.5**	43.4 - 47.6
	**Men**	**84.4**	84.3 - 84.4	**62.0**	61.6 - 62.4	**55.6**	54.8 - 56.4	**45.5**	43.4 - 47.6
	**Women**								
**Leukaemia**									
	**All persons**	**73.9**	73.8 - 73.9	**56.4**	56.1 - 56.6	**52.7**	52.3 - 53.1	**49.0**	48.4 - 49.6
	**Men**	**75.8**	75.8 - 75.9	**57.2**	56.8 - 57.5	**53.0**	52.6 - 53.5	**48.9**	48.3 - 49.5
	**Women**	**71.4**	71.2 - 71.5	**55.4**	54.8 - 55.9	**52.2**	51.5 - 53.0	**49.1**	48.1 - 50.2
**Lung**									
	**All persons**	**42.8**	42.6 - 43.0	**17.4**	16.7 - 18.2	**14.1**	13.1 - 15.1	**11.1**	9.9 - 12.4
	**Men**	**39.6**	39.3 - 39.9	**14.9**	13.7 - 16.1	**11.4**	9.9 - 13.1	**8.5**	6.5 - 10.7
	**Women**	**48.3**	48.1 - 48.5	**21.9**	21.0 - 22.8	**18.6**	17.5 - 19.7	**15.7**	14.4 - 17.1
**Melanoma**									
	**All persons**	**98.6**	98.6 - 98.6	**93.6**	93.6 - 93.6	**92.8**	92.8 - 92.8	**92.7**	92.7 - 92.7
	**Men**	**98.0**	98.0 - 98.0	**91.6**	91.6 - 91.7	**90.7**	90.7 - 90.7	**90.2**	90.2 - 90.3
	**Women**	**99.0**	99.0 - 99.0	**95.0**	95.0 - 95.0	**94.5**	94.5 - 94.5	**94.6**	94.6 - 94.6
**Myeloma**									
	**All persons**	**84.5**	84.5 - 84.5	**57.0**	56.6 - 57.4	**47.9**	47.0 - 48.9	**37.8**	35.5 - 40.0
	**Men**	**85.6**	85.6 - 85.6	**57.9**	57.4 - 58.4	**48.7**	47.5 - 50.0	**38.5**	35.5 - 41.5
	**Women**	**83.3**	83.2 - 83.3	**56.0**	55.4 - 56.7	**47.0**	45.6 - 48.5	**36.9**	33.6 - 40.3
**Non-Hodgkin lymphoma**									
	**All persons**	**82.3**	82.3 - 82.3	**71.2**	71.1 - 71.3	**68.2**	68.1 - 68.3	**64.6**	64.3 - 64.8
	**Men**	**82.5**	82.5 - 82.5	**70.6**	70.5 - 70.7	**67.3**	67.1 - 67.5	**63.4**	63.0 - 63.8
	**Women**	**82.1**	82.1 - 82.2	**71.9**	71.8 - 72.1	**69.2**	69.0 - 69.4	**65.9**	65.5 - 66.2
**Oesophagus**									
	**All persons**	**45.5**	45.1 - 45.9	**16.8**	14.8 - 19.0	**14.6**	11.9 - 17.5	**13.0**	9.6 - 16.9
	**Men**	**47.9**	47.4 - 48.3	**16.8**	14.3 - 19.5	**14.1**	10.8 - 17.9	**12.1**	7.8 - 17.4
	**Women**	**41.8**	40.8 - 42.7	**16.9**	13.5 - 20.6	**15.3**	11.1 - 20.1	**14.3**	9.2 - 20.6
**Others**									
	**All persons**	**69.2**	69.1 - 69.2	**49.2**	49.0 - 49.4	**46.3**	46.0 - 46.6	**43.7**	43.3 - 44.2
	**Men**	**69.5**	69.4 - 69.5	**47.5**	47.2 - 47.8	**44.3**	43.9 - 44.8	**41.5**	40.9 - 42.2
	**Women**	**68.8**	68.8 - 68.9	**51.2**	50.9 - 51.5	**48.5**	48.1 - 48.9	**46.2**	45.6 - 46.8
**Ovary**									
	**All persons**	**78.1**	78.0 - 78.1	**52.1**	51.8 - 52.4	**45.8**	45.2 - 46.3	**39.3**	38.3 - 40.3
	**Men**								
	**Women**	**78.1**	78.0 - 78.1	**52.1**	51.8 - 52.4	**45.8**	45.2 - 46.3	**39.3**	38.3 - 40.3
**Pancreas**									
	**All persons**	**24.8**	23.8 - 25.8	**7.2**	4.6 - 10.5	**5.6**	2.7 - 9.8	**4.3**	1.4 - 9.9
	**Men**	**25.8**	24.5 - 27.2	**7.3**	3.9 - 12.1	**5.5**	2.0 - 11.9	**4.1**	0.7 - 13.0
	**Women**	**23.8**	22.3 - 25.4	**7.1**	3.6 - 12.0	**5.6**	1.9 - 12.1	**4.4**	0.8 - 13.3

Appendix Table 2. Standardised net survival at 1, 5, 7, and 10 years for all cancers combined (CSI) and for 22 selected cancers, by sex: adults diagnosed in England and Wales during 2018

		1 year		5 year		7 year		10 year	
		**NS**	**95% CI**	**NS**	**95% CI**	**NS**	**95% CI**	**NS**	**95% CI**
**Prostate**									
	**All persons**	**95.1**	95.1 - 95.1	**85.7**	85.7 - 85.8	**82.3**	82.3 - 82.3	**78.9**	78.9 - 79.0
	**Men**	**95.1**	95.1 - 95.1	**85.7**	85.7 - 85.8	**82.3**	82.3 - 82.3	**78.9**	78.9 - 79.0
	**Women**								
**Rectum**									
	**All persons**	**82.5**	82.4 - 82.5	**59.6**	59.4 - 59.8	**56.2**	55.9 - 56.5	**53.9**	53.4 - 54.4
	**Men**	**83.8**	83.8 - 83.8	**60.1**	59.9 - 60.4	**56.3**	55.9 - 56.7	**53.6**	53.0 - 54.3
	**Women**	**80.6**	80.5 - 80.6	**58.9**	58.5 - 59.2	**56.0**	55.5 - 56.5	**54.2**	53.5 - 55.0
**Stomach**									
	**All persons**	**45.2**	44.8 - 45.7	**20.8**	19.1 - 22.6	**18.2**	16.1 - 20.5	**16.1**	13.3 - 19.0
	**Men**	**46.9**	46.4 - 47.4	**20.7**	18.6 - 22.9	**17.8**	15.1 - 20.7	**15.4**	11.9 - 19.2
	**Women**	**42.2**	41.3 - 43.1	**21.0**	18.2 - 24.0	**18.9**	15.5 - 22.7	**17.3**	13.1 - 22.0
**Testis**									
	**All persons**	**98.4**	98.4 - 98.4	**97.0**	97.0 - 97.0	**96.8**	96.8 - 96.8	**96.5**	96.5 - 96.5
	**Men**	**98.4**	98.4 - 98.4	**97.0**	97.0 - 97.0	**96.8**	96.8 - 96.8	**96.5**	96.5 - 96.5
	**Women**								
**Uterus**									
	**All persons**	**90.0**	90.0 - 90.0	**76.6**	76.5 - 76.7	**74.2**	74.1 - 74.3	**71.7**	71.5 - 71.9
	**Men**								
	**Women**	**90.0**	90.0 - 90.0	**76.6**	76.5 - 76.7	**74.2**	74.1 - 74.3	**71.7**	71.5 - 71.9

Appendix Table 3. Age-sex-cancer weights: to produce the cancer survival index for all adults, all cancers and all ages combined

Sex	Age group (years)	Weights: Bladder	Brain	Breast	Cervix	Colon	HL
**Men**	**15-44**	0.0006956	0.0017485	-	-	0.0011023	0.0019679
**Men**	**45-54**	0.0023960	0.0016976	-	-	0.0030582	0.0004227
**Men**	**55-64**	0.0066648	0.0021752	-	-	0.0079584	0.0003866
**Men**	**65-74**	0.0135838	0.0023839	-	-	0.0148707	0.0003304
**Men**	**75-99**	0.0157871	0.0012910	-	-	0.0157817	0.0001793
**Women**	**15-44**	0.0002836	0.0012816	0.0203422	0.0059946	0.0010475	0.0015639
**Women**	**45-54**	0.0007625	0.0009552	0.0394991	0.0023144	0.0027906	0.0002515
**Women**	**55-64**	0.0019973	0.0014087	0.0371995	0.0017418	0.0060026	0.0002354
**Women**	**65-74**	0.0044026	0.0018033	0.0322765	0.0017016	0.0121523	0.0002408
**Women**	**75-99**	0.0077310	0.0012548	0.0379968	0.0020829	0.0211395	0.0002167
		0.0543043	0.0159998	0.1673141	0.0138353	0.0859038	0.0057952

Sex	Age group (years)	Weights: Kidney	Larynx	Leukaemia	Lung	Melanoma	Myeloma
**Men**	**15-44**	0.0007264	0.0002114	0.0015304	0.0010448	0.0023505	0.0002314
**Men**	**45-54**	0.0019063	0.0010836	0.0012843	0.0060160	0.0019893	0.0006689
**Men**	**55-64**	0.0032883	0.0020374	0.0024093	0.0173830	0.0022127	0.0013993
**Men**	**65-74**	0.0045458	0.0025284	0.0038448	0.0349266	0.0022715	0.0023786
**Men**	**75-99**	0.0034327	0.0017190	0.0042622	0.0314002	0.0018528	0.0024107
**Women**	**15-44**	0.0004669	-	0.0010970	0.0009351	0.0038836	0.0001405
**Women**	**45-54**	0.0009405	-	0.0009766	0.0041043	0.0027304	0.0004441
**Women**	**55-64**	0.0016267	-	0.0014194	0.0089912	0.0024013	0.0010475
**Women**	**65-74**	0.0025043	-	0.0024040	0.0197014	0.0025364	0.0018501
**Women**	**75-99**	0.0027411	-	0.0044227	0.0190432	0.0029337	0.0028776
		0.0221790	0.0075798	0.0236507	0.1435458	0.0251622	0.0134487

Sex	Age group (years)	Weights: NHL	Oesophagus	Others	Ovary	Pancreas	Prostate
**Men**	**15-44**	0.0024802	0.0004013	0.0042260	-	0.0003064	0.0000896
**Men**	**45-54**	0.0027224	0.0017659	0.0061765	-	0.0010930	0.0019598
**Men**	**55-64**	0.0039170	0.0035438	0.0094527	-	0.0024816	0.0138700
**Men**	**65-74**	0.0050621	0.0056775	0.0123022	-	0.0038889	0.0372035
**Men**	**75-99**	0.0044815	0.0057444	0.0112467	-	0.0040508	0.0457439
**Women**	**15-44**	0.0015277	0.0001204	0.0046073	0.0029873	0.0002100	-
**Women**	**45-54**	0.0020508	0.0005271	0.0042314	0.0046528	0.0007104	-
**Women**	**55-64**	0.0030327	0.0012856	0.0057712	0.0061618	0.0016963	-
**Women**	**65-74**	0.0042247	0.0030501	0.0089484	0.0068173	0.0034541	-
**Women**	**75-99**	0.0055906	0.0060093	0.0144118	0.0063705	0.0060788	-
		0.0350897	0.0281254	0.0813742	0.0269897	0.0239703	0.0988668

Sex	Age group (years)	Weights: Rectum	Stomach	Testis	Uterus		All cancers
**Men**	**15-44**	0.0007826	0.0005579	0.0066206	-		0.0270738
**Men**	**45-54**	0.0030434	0.0017244	0.0010448	-		0.0400531
**Men**	**55-64**	0.0073698	0.0044347	0.0003371	-		0.0913217
**Men**	**65-74**	0.0116212	0.0092949	0.0001391	-		0.1668539
**Men**	**75-99**	0.0102246	0.0101457	0.0000883	-		0.1698426
**Women**	**15-44**	0.0007746	0.0003585	-	0.0006756		0.0482979
**Women**	**45-54**	0.0020655	0.0006020	-	0.0030809		0.0736901
**Women**	**55-64**	0.0036615	0.0015518	-	0.0063250		0.0935573
**Women**	**65-74**	0.0064534	0.0037993	-	0.0064253		0.1247459
**Women**	**75-99**	0.0101390	0.0078929	-	0.0056307		0.1645636
		0.0561356	0.0403621	0.0082299	0.0221375		1

All weights are based on the numbers of patients diagnosed in England and Wales, 1996-99

Appendix Table 4. Age-cancer weights for each sex: to produce a separate cancer survival index for men and women, for all cancers and all ages combined

Weights

Age group	Bladder		Brain		Breast		Cervix		Colon	
**(years)**	**Men**	**Women**	**Men**	**Women**	**Men**	**Women**	**Men**	**Women**	**Men**	**Women**
**15-44**	0.0014049	0.0005618	0.0035312	0.0025385	-	0.0402932	-	0.0118738	0.0022263	0.0020748
**45-54**	0.0048389	0.0015104	0.0034286	0.0018920	-	0.0782386	-	0.0045842	0.0061763	0.0055275
**55-64**	0.0134603	0.0039562	0.0043931	0.0027903	-	0.0736836	-	0.0034501	0.0160729	0.0118897
**65-74**	0.0274339	0.0087206	0.0048146	0.0035720	-	0.0639322	-	0.0033706	0.0300330	0.0240710
**75-99**	0.0318837	0.0153133	0.0026072	0.0024855	-	0.0752629	-	0.0041258	0.0318729	0.0418725
	0.0790217	0.0300623	0.0187747	0.0132783	-	0.3314105	-	0.0274045	0.0863814	0.0854355

Weights

Age group	Hodgkin's lymphoma		Kidney		Larynx		Leukaemia		Lung	
**(years)**	**Men**	**Women**	**Men**	**Women**	**Men**	**Women**	**Men**	**Women**	**Men**	**Women**
**15-44**	0.0039743	0.0030976	0.0014671	0.0009248	0.0004269	-	0.0030908	0.0021729	0.0021101	0.0018522
**45-54**	0.0008538	0.0004982	0.0038500	0.0018628	0.0021884	-	0.0025937	0.0019344	0.0121499	0.0081297
**55-64**	0.0007808	0.0004664	0.0066410	0.0032222	0.0041148	-	0.0048659	0.0028115	0.0351070	0.0178094
**65-74**	0.0006673	0.0004770	0.0091807	0.0049605	0.0051064	-	0.0077649	0.0047617	0.0705381	0.0390239
**75-99**	0.0003620	0.0004293	0.0069328	0.0054295	0.0034718	-	0.0086079	0.0087603	0.0634162	0.0377202
	0.0066382	0.0049685	0.0280716	0.0163998	0.0153083	-	0.0269232	0.0204408	0.1833213	0.1045354

Weights

Age group	Melanoma		Myeloma		Non-Hodgkin lymphoma		Oesophagus		Others	
**(years)**	**Men**	**Women**	**Men**	**Women**	**Men**	**Women**	**Men**	**Women**	**Men**	**Women**
**15-44**	0.0047470	0.0076924	0.0004674	0.0002782	0.0050091	0.0030261	0.0008105	0.0002385	0.0085349	0.0091260
**45-54**	0.0040176	0.0054083	0.0013509	0.0008797	0.0054981	0.0040622	0.0035664	0.0010440	0.0124741	0.0083814
**55-64**	0.0044687	0.0047564	0.0028261	0.0020748	0.0079108	0.0060071	0.0071570	0.0025465	0.0190908	0.0114313
**65-74**	0.0045876	0.0050241	0.0048038	0.0036647	0.0102235	0.0083681	0.0114664	0.0060416	0.0248456	0.0177247
**75-99**	0.0037420	0.0058111	0.0048686	0.0056998	0.0090510	0.0110736	0.0116015	0.0119030	0.0227139	0.0285465
	0.0215629	0.0286923	0.0143168	0.0125972	0.0376925	0.0325371	0.0346018	0.0217736	0.0876593	0.0752099

Weights

Age group	Ovary		Pancreas		Prostate		Rectum		Stomach	
**(years)**	**Men**	**Women**	**Men**	**Women**	**Men**	**Women**	**Men**	**Women**	**Men**	**Women**
**15-44**	-	0.0059170	0.0006187	0.0004160	0.0001810	-	0.0015805	0.0015342	0.0011266	0.0007102
**45-54**	-	0.0092161	0.0022074	0.0014071	0.0039581	-	0.0061466	0.0040913	0.0034826	0.0011924
**55-64**	-	0.0122051	0.0050118	0.0033600	0.0280121	-	0.0148841	0.0072526	0.0089564	0.0030738
**65-74**	-	0.0135035	0.0078541	0.0068418	0.0751366	-	0.0234704	0.0127827	0.0187720	0.0075255
**75-99**	-	0.0126184	0.0081810	0.0120408	0.0923848	-	0.0206497	0.0200830	0.0204903	0.0156339
	-	0.0534601	0.0238730	0.0240657	0.1996726	-	0.0667313	0.0457438	0.0528279	0.0281358

Weights

Age group	Testis		Uterus		All cancers	
**(years)**	**Men**	**Women**	**Men**	**Women**	**Men**	**Women**
**15-44**	0.0133711	-	-	0.0013382	0.0546784	0.0956664
**45-54**	0.0021101	-	-	0.0061025	0.0808915	0.1459628
**55-64**	0.0006808	-	-	0.0125284	0.1844344	0.1853154
**65-74**	0.0002810	-	-	0.0127271	0.3369799	0.2470933
**75-99**	0.0001783	-	-	0.0111531	0.3430156	0.3259625
	0.0166213	-	-	0.0438493	1	1

All weights are based on the numbers of patients diagnosed in England and Wales, 1996-99

Appendix Table 5. Age-sex weights for each cancer: to produce age- and sex-standardised survival estimates for each cancer

Sex	Age group (years)	Weights: Bladder	Brain	Breast	Cervix	Colon	HL
**Men**	**15-44**	0.0128101	0.1092809	-	-	0.0128321	0.3395660
**Men**	**45-54**	0.0441209	0.1061037	-	-	0.0355997	0.0729455
**Men**	**55-64**	0.1227305	0.1359532	-	-	0.0926434	0.0667128
**Men**	**65-74**	0.2501417	0.1489967	-	-	0.1731087	0.0570175
**Men**	**75-99**	0.2907152	0.0806856	-	-	0.1837138	0.0309326
**Women**	**15-44**	0.0052226	0.0801003	0.1215809	0.4332817	0.0121936	0.2698523
**Women**	**45-54**	0.0140418	0.0596990	0.2360777	0.1672790	0.0324851	0.0433980
**Women**	**55-64**	0.0367797	0.0880435	0.2223333	0.1258944	0.0698757	0.0406279
**Women**	**65-74**	0.0810731	0.1127090	0.1929095	0.1229936	0.1414645	0.0415512
**Women**	**75-99**	0.1423644	0.0784281	0.2270986	0.1505512	0.2460834	0.0373961
		1	1	1	1	1	1

Sex	Age group (years)	Weights: Kidney	Larynx	Leukaemia	Lung	Melanoma	Myeloma
**Men**	**15-44**	0.0327523	0.0278856	0.0647095	0.0072785	0.0934127	0.0172088
**Men**	**45-54**	0.0859521	0.1429580	0.0543017	0.0419098	0.0790579	0.0497364
**Men**	**55-64**	0.1482598	0.2687963	0.1018723	0.1210975	0.0879366	0.1040485
**Men**	**65-74**	0.2049581	0.3335687	0.1625658	0.2433133	0.0902759	0.1768626
**Men**	**75-99**	0.1547741	0.2267914	0.1802138	0.2187471	0.0736350	0.1792500
**Women**	**15-44**	0.0210507	-	0.0463827	0.0065143	0.1543410	0.0104446
**Women**	**45-54**	0.0424030	-	0.0412919	0.0285922	0.1085119	0.0330250
**Women**	**55-64**	0.0733458	-	0.0600147	0.0626363	0.0954330	0.0778872
**Women**	**65-74**	0.1129139	-	0.1016460	0.1372481	0.1008028	0.1375709
**Women**	**75-99**	0.1235901	-	0.1870015	0.1326630	0.1165931	0.2139660
		1	1	1	1	1	1

Sex	Age group (years)	Weights: NHL	Oesophagus	Others	Ovary	Pancreas	Prostate
**Men**	**15-44**	0.0706824	0.0142694	0.0519333	-	0.0127804	0.0009066
**Men**	**45-54**	0.0775829	0.0627854	0.0759025	-	0.0455966	0.0198230
**Men**	**55-64**	0.1116279	0.1259989	0.1161636	-	0.1035272	0.1402901
**Men**	**65-74**	0.1442623	0.2018645	0.1511804	-	0.1622391	0.3762990
**Men**	**75-99**	0.1277164	0.2042428	0.1382094	-	0.1689921	0.4626813
**Women**	**15-44**	0.0435379	0.0042808	0.0566187	0.1106815	0.0087621	-
**Women**	**45-54**	0.0584445	0.0187405	0.0519991	0.1723916	0.0296350	-
**Women**	**55-64**	0.0864278	0.0457097	0.0709213	0.2283024	0.0707668	-
**Women**	**65-74**	0.1203965	0.1084475	0.1099658	0.2525899	0.1441009	-
**Women**	**75-99**	0.1593214	0.2136606	0.1771059	0.2360347	0.2535997	-
		1	1	1	1	1	1

Sex	Age group (years)	Weights: Rectum	Stomach	Testis	Uterus
**Men**	**15-44**	0.0139412	0.0138212	0.8044538	-
**Men**	**45-54**	0.0542157	0.0427231	0.1269506	-
**Men**	**55-64**	0.1312854	0.1098737	0.0409623	-
**Men**	**65-74**	0.2070206	0.2302874	0.0169051	-
**Men**	**75-99**	0.1821410	0.2513672	0.0107282	-
**Women**	**15-44**	0.0137982	0.0088827	-	0.0305173
**Women**	**45-54**	0.0367952	0.0149150	-	0.1391709
**Women**	**55-64**	0.0652257	0.0384475	-	0.2857143
**Women**	**65-74**	0.1149612	0.0941301	-	0.2902465
**Women**	**75-99**	0.1806158	0.1955520	-	0.2543510
		1	1	1	1

All weights are based on the numbers of patients diagnosed in England and Wales, 1996-99

Appendix Table 6. Age weights for each cancer and each sex: to produce age-standardised survival estimates for each cancer in men and women

Age group	Weights									
	Bladder		Brain		Breast		Cervix		Colon	
(years)	Men	Women	Men	Women	Men	Women	Men	Women	Men	Women
**15-44**	0.0177790	0.0186866	0.1880846	0.1911794	-	0.1215809	-	0.4332817	0.0257726	0.0242851
**45-54**	0.0612350	0.0502424	0.1826162	0.1424865	-	0.2360777	-	0.1672790	0.0715001	0.0646982
**55-64**	0.1703364	0.1315998	0.2339905	0.2101377	-	0.2223333	-	0.1258944	0.1860691	0.1391663
**65-74**	0.3471690	0.2900837	0.2564398	0.2690082	-	0.1929095	-	0.1229936	0.3476792	0.2817443
**75-99**	0.4034806	0.5093874	0.1388689	0.1871882	-	0.2270986	-	0.1505512	0.3689791	0.4901061
	1	1	1	1	-	1	-	1	1	1

Age group	Weights									
	Hodgkin's lymphoma		Kidney		Larynx		Leukaemia		Lung	
(years)	Men	Women	Men	Women	Men	Women	Men	Women	Men	Women
**15-44**	0.5986976	0.6234667	0.0522618	0.0563904	0.0278856	-	0.1148018	0.1063002	0.0115103	0.0177186
**45-54**	0.1286121	0.1002667	0.1371511	0.1135886	0.1429580	-	0.0963372	0.0946331	0.0662766	0.0777693
**55-64**	0.1176231	0.0938667	0.2365736	0.1964776	0.2687963	-	0.1807326	0.1375421	0.1915050	0.1703676
**65-74**	0.1005291	0.0960000	0.3270452	0.3024721	0.3335687	-	0.2884094	0.2329531	0.3847786	0.3733080
**75-99**	0.0545381	0.0864000	0.2469682	0.3310713	0.2267914	-	0.3197190	0.4285714	0.3459294	0.3608365
	1	1	1	1	1	-	1	1	1	1

Age group	Weights									
	Melanoma		Myeloma		Non-Hodgkin lymphoma		Oesophagus		Others	
(years)	Men	Women	Men	Women	Men	Women	Men	Women	Men	Women
**15-44**	0.2201478	0.2681012	0.0326477	0.0220867	0.1328937	0.0930043	0.0234247	0.0109529	0.0973648	0.1213402
**45-54**	0.1863175	0.1884928	0.0943574	0.0698359	0.1458677	0.1248473	0.1030686	0.0479494	0.1423024	0.1114399
**55-64**	0.2072422	0.1657739	0.1973957	0.1647034	0.2098774	0.1846242	0.2068400	0.1169527	0.2177839	0.1519924
**65-74**	0.2127553	0.1751016	0.3355350	0.2909129	0.2712350	0.2571871	0.3313813	0.2774735	0.2834335	0.2356692
**75-99**	0.1735372	0.2025305	0.3400642	0.4524611	0.2401262	0.3403372	0.3352854	0.5466715	0.2591154	0.3795582
	1	1	1	1	1	1	1	1	1	1

Age group	Weights									
	Ovary		Pancreas		Prostate		Rectum		Stomach	
(years)	Men	Women	Men	Women	Men	Women	Men	Women	Men	Women
**15-44**	-	0.1106815	0.0259167	0.0172869	0.0009066	-	0.0236852	0.0335399	0.0213266	0.0252402
**45-54**	-	0.1723916	0.0924627	0.0584673	0.0198230	-	0.0921090	0.0894398	0.0659234	0.0423809
**55-64**	-	0.2283024	0.2099366	0.1396168	0.1402901	-	0.2230455	0.1585472	0.1695392	0.1092484
**65-74**	-	0.2525899	0.3289950	0.2842986	0.3762990	-	0.3517146	0.2794416	0.3553419	0.2674703
**75-99**	-	0.2360347	0.3426890	0.5003303	0.4626813	-	0.3094457	0.4390315	0.3878689	0.5556602
	-	1	1	1	1	-	1	1	1	1

Age group	Weights			
	Testis		Uterus	
(years)	Men	Women	Men	Women
**15-44**	0.8044538	-	-	0.0305173
**45-54**	0.1269506	-	-	0.1391709
**55-64**	0.0409623	-	-	0.2857143
**65-74**	0.0169051	-	-	0.2902465
**75-99**	0.0107282	-	-	0.2543510
	1	-	-	1

All weights are based on the numbers of patients diagnosed in England and Wales, 1996-99

Appendix Table 7. One-year survival by age and sex, for each cancer: adults diagnosed in England and Wales during 1971-72

Age group	One-year survival									
	Bladder		Brain		Breast		Cervix		Colon	
(years)	Men	Women	Men	Women	Men	Women	Men	Women	Men	Women
**15-44**	90.2	83.6	48.5	48.0	-	88.5	-	83.9	58.8	64.0
**45-54**	82.5	77.0	25.0	26.2	-	87.6	-	78.6	51.6	54.3
**55-64**	74.5	69.4	12.2	12.9	-	82.8	-	72.4	47.7	49.3
**65-74**	66.0	60.2	6.1	6.5	-	76.5	-	63.5	42.1	42.8
**75-99**	47.9	40.1	2.6	4.0	-	60.9	-	45.9	29.0	26.8

Age group	One-year survival									
	Hodgkin's lymphoma		Kidney		Larynx		Leukaemia		Lung	
(years)	Men	Women	Men	Women	Men	Women	Men	Women	Men	Women
**15-44**	86.2	90.1	58.1	56.3	85.3	-	46.0	42.5	29.6	32.4
**45-54**	70.3	75.4	51.8	51.4	84.6	-	47.8	44.0	28.1	24.7
**55-64**	54.0	59.9	46.0	46.4	82.1	-	45.9	44.0	23.0	18.8
**65-74**	37.0	45.0	40.3	39.4	76.3	-	39.1	37.7	15.8	14.0
**75-99**	22.0	28.5	28.3	27.0	62.6	-	29.2	25.1	7.9	8.3

Age group	One-year survival									
	Melanoma		Myeloma		Non-Hodgkin lymphoma		Oesophagus		Others	
(years)	Men	Women	Men	Women	Men	Women	Men	Women	Men	Women
**15-44**	78.6	90.3	67.7	58.0	61.8	65.7	28.4	30.0	62.8	69.0
**45-54**	75.0	88.0	53.9	52.5	61.2	64.5	20.9	26.3	50.5	53.8
**55-64**	71.8	85.4	44.2	47.4	54.6	58.3	17.4	23.4	41.9	43.1
**65-74**	66.5	81.3	36.2	40.8	45.4	48.1	14.6	19.7	35.8	34.0
**75-99**	57.4	70.9	23.5	28.5	32.2	34.9	8.8	10.6	33.6	26.2

Age group	One-year survival									
	Ovary		Pancreas		Prostate		Rectum		Stomach	
(years)	Men	Women	Men	Women	Men	Women	Men	Women	Men	Women
**15-44**	-	66.6	14.2	18.4	64.1	-	63.6	66.3	28.5	27.9
**45-54**	-	55.0	11.5	12.8	64.4	-	62.0	64.1	24.4	24.5
**55-64**	-	45.1	10.0	10.8	65.7	-	58.7	60.6	19.8	20.8
**65-74**	-	34.8	9.0	9.9	66.3	-	51.5	52.9	15.0	16.4
**75-99**	-	23.1	6.9	7.4	53.8	-	35.9	33.5	8.4	9.0

Age group	One-year survival			
	Testis		Uterus	
(years)	Men	Women	Men	Women
**15-44**	80.2	-	-	90.0
**45-54**	79.0	-	-	87.6
**55-64**	69.9	-	-	82.8
**65-74**	54.9	-	-	72.5
**75-99**	32.1	-	-	50.5

Appendix Table 7. One-year survival by age and sex, for each cancer: adults diagnosed in England and Wales during 1980-81

Age group (years)	Bladder		Brain		Breast		Cervix		Colon	
	Men	Women	Men	Women	Men	Women	Men	Women	Men	Women
**15-44**	92.5	86.6	57.0	57.8	-	90.3	-	87.6	66.6	70.6
**45-54**	87.2	81.2	30.5	33.4	-	89.9	-	81.2	61.3	63.7
**55-64**	82.3	76.5	15.7	16.9	-	86.4	-	74.6	58.3	59.8
**65-74**	75.9	69.1	7.3	7.9	-	80.3	-	65.4	52.5	53.2
**75-99**	62.8	51.3	3.1	4.3	-	64.2	-	46.8	39.6	36.0

One-year survival

Age group (years)	Hodgkin's lymphoma		Kidney		Larynx		Leukaemia		Lung	
	Men	Women	Men	Women	Men	Women	Men	Women	Men	Women
**15-44**	92.5	93.6	65.6	63.5	86.7	-	57.1	55.1	32.3	34.8
**45-54**	81.1	82.6	58.3	57.8	85.8	-	57.5	55.5	29.1	26.8
**55-64**	67.6	70.6	52.6	52.3	83.7	-	54.5	54.4	24.3	21.5
**65-74**	50.4	54.2	46.3	44.1	78.8	-	45.7	46.5	17.4	16.3
**75-99**	34.3	36.7	34.1	30.7	66.0	-	33.9	31.8	9.9	10.0

One-year survival

Age group (years)	Melanoma		Myeloma		Non-Hodgkin lymphoma		Oesophagus		Others	
	Men	Women	Men	Women	Men	Women	Men	Women	Men	Women
**15-44**	87.0	94.4	74.2	70.2	69.9	73.8	30.7	35.5	68.4	78.2
**45-54**	84.4	92.5	64.1	63.8	68.2	72.2	24.4	31.3	56.0	63.0
**55-64**	82.1	90.8	55.3	58.1	62.0	66.4	21.4	28.0	46.6	52.1
**65-74**	78.5	87.6	45.1	49.7	51.7	54.9	17.9	23.2	38.0	40.6
**75-99**	72.0	79.6	30.7	34.6	38.3	39.7	11.4	12.1	33.0	30.0

One-year survival

Age group (years)	Ovary		Pancreas		Prostate		Rectum		Stomach	
	Men	Women	Men	Women	Men	Women	Men	Women	Men	Women
**15-44**	-	75.2	17.5	22.9	77.8	-	70.0	73.4	33.5	34.3
**45-54**	-	63.4	13.7	15.1	76.7	-	69.2	71.8	29.2	30.5
**55-64**	-	53.0	11.7	12.3	75.8	-	66.4	68.9	24.7	26.8
**65-74**	-	40.4	9.9	10.3	73.1	-	59.0	60.8	18.9	21.3
**75-99**	-	25.8	7.2	6.8	57.8	-	43.9	41.0	11.8	12.1

One-year survival

Age group (years)	Testis		Uterus	
	Men	Women	Men	Women
**15-44**	91.3	-	-	90.7
**45-54**	89.4	-	-	89.1
**55-64**	83.8	-	-	85.6
**65-74**	71.9	-	-	76.5
**75-99**	48.9	-	-	56.0

Appendix Table 7. One-year survival by age and sex, for each cancer: adults diagnosed in England and Wales during 1990-91

Age group	One-year survival									
	Bladder		Brain		Breast		Cervix		Colon	
(years)	Men	Women	Men	Women	Men	Women	Men	Women	Men	Women
**15-44**	92.9	87.2	66.1	67.7	-	93.4	-	90.8	73.8	77.0
**45-54**	89.7	83.4	39.0	43.0	-	93.4	-	84.9	70.5	72.8
**55-64**	86.0	79.2	20.4	22.8	-	91.0	-	77.9	68.1	69.6
**65-74**	81.4	73.7	9.6	10.1	-	86.6	-	68.8	63.2	63.8
**75-99**	69.5	57.1	3.5	4.8	-	71.5	-	49.9	49.7	45.9

Age group	One-year survival									
	Hodgkin's lymphoma		Kidney		Larynx		Leukaemia		Lung	
(years)	Men	Women	Men	Women	Men	Women	Men	Women	Men	Women
**15-44**	95.8	96.3	72.7	71.8	88.3	-	68.9	67.1	35.3	38.0
**45-54**	87.7	88.9	66.1	66.3	87.4	-	67.9	66.3	30.9	31.2
**55-64**	77.4	78.2	60.4	59.9	85.4	-	63.9	64.2	25.6	25.0
**65-74**	62.4	65.1	54.3	51.6	81.5	-	54.8	56.2	20.2	20.0
**75-99**	43.1	46.7	40.4	35.5	71.1	-	39.9	38.7	12.2	12.8

Age group	One-year survival									
	Melanoma		Myeloma		Non-Hodgkin lymphoma		Oesophagus		Others	
(years)	Men	Women	Men	Women	Men	Women	Men	Women	Men	Women
**15-44**	92.7	97.0	80.8	80.0	77.8	81.3	35.0	43.2	73.8	83.9
**45-54**	91.1	95.7	74.5	75.2	75.7	79.8	31.2	38.8	63.2	71.8
**55-64**	89.5	94.4	66.5	68.6	69.4	74.1	28.2	34.8	52.4	60.1
**65-74**	87.3	92.5	56.4	59.6	60.0	63.2	24.0	28.9	41.6	47.8
**75-99**	82.6	86.3	38.3	41.4	45.0	46.2	15.5	15.1	33.3	33.5

Age group	One-year survival									
	Ovary		Pancreas		Prostate		Rectum		Stomach	
(years)	Men	Women	Men	Women	Men	Women	Men	Women	Men	Women
**15-44**	-	83.6	21.6	28.7	89.5	-	77.0	80.9	42.2	43.4
**45-54**	-	73.1	17.2	19.4	88.4	-	76.9	80.1	38.1	40.0
**55-64**	-	62.2	14.1	14.8	86.5	-	74.5	77.5	33.1	35.5
**65-74**	-	48.7	11.4	11.7	82.8	-	68.1	70.6	27.1	29.8
**75-99**	-	29.7	7.6	6.7	66.3	-	53.5	50.7	17.7	17.8

Age group	One-year survival			
	Testis		Uterus	
(years)	Men	Women	Men	Women
**15-44**	96.5	-	-	92.0
**45-54**	95.2	-	-	91.4
**55-64**	91.8	-	-	88.8
**65-74**	84.2	-	-	81.9
**75-99**	64.1	-	-	62.7

Appendix Table 7. One-year survival by age and sex, for each cancer: adults diagnosed in England and Wales during 2000-01

Age group (years)	Bladder		Brain		Breast		Cervix		Colon	
	One-year survival									
	Men	Women	Men	Women	Men	Women	Men	Women	Men	Women
**15-44**	91.9	84.4	74.6	76.7	-	96.7	-	93.5	79.9	82.7
**45-54**	89.2	81.4	47.7	51.6	-	96.9	-	88.1	77.8	79.7
**55-64**	86.3	78.0	27.3	29.3	-	95.9	-	81.9	76.2	77.4
**65-74**	81.7	72.1	13.5	13.2	-	93.2	-	72.1	71.7	72.1
**75-99**	70.2	55.5	4.9	5.2	-	82.6	-	52.7	59.3	55.3

Age group (years)	Hodgkin's lymphoma		Kidney		Larynx		Leukaemia		Lung	
	One-year survival									
	Men	Women	Men	Women	Men	Women	Men	Women	Men	Women
**15-44**	97.1	97.8	80.7	81.2	89.7	-	79.3	76.3	40.6	44.0
**45-54**	91.0	92.7	74.7	76.3	88.7	-	77.6	74.9	34.3	37.5
**55-64**	82.2	84.6	69.6	70.8	87.2	-	73.7	72.6	29.4	31.9
**65-74**	68.1	73.7	63.7	62.3	84.0	-	64.2	63.9	24.5	25.8
**75-99**	48.5	54.9	50.0	45.5	74.4	-	48.3	45.8	16.6	17.7

Age group (years)	Melanoma		Myeloma		Non-Hodgkin lymphoma		Oesophagus		Others	
	One-year survival									
	Men	Women	Men	Women	Men	Women	Men	Women	Men	Women
**15-44**	95.9	98.4	87.6	88.2	84.7	87.7	44.5	52.9	80.1	88.7
**45-54**	94.9	97.5	83.3	83.7	82.3	86.3	42.2	48.8	70.7	78.1
**55-64**	94.0	96.8	77.5	78.3	77.2	81.8	39.9	44.6	60.4	67.7
**65-74**	92.5	95.4	67.6	69.5	68.2	71.8	34.5	37.2	47.3	53.5
**75-99**	89.4	91.2	49.6	49.1	53.0	53.8	23.8	20.3	35.7	36.2

Age group (years)	Ovary		Pancreas		Prostate		Rectum		Stomach	
	One-year survival									
	Men	Women	Men	Women	Men	Women	Men	Women	Men	Women
**15-44**	-	90.4	28.9	36.1	97.0	-	83.8	87.3	51.9	52.3
**45-54**	-	82.1	22.5	25.7	96.3	-	83.9	86.9	48.1	49.2
**55-64**	-	73.0	18.3	20.1	95.3	-	82.2	85.0	43.5	44.9
**65-74**	-	59.0	14.2	14.9	92.8	-	76.9	79.2	36.8	38.3
**75-99**	-	37.0	8.8	7.8	81.5	-	64.4	61.9	26.1	24.6

Age group (years)	Testis		Uterus	
	One-year survival			
	Men	Women	Men	Women
**15-44**	98.3	-	-	94.0
**45-54**	97.4	-	-	94.1
**55-64**	95.3	-	-	92.5
**65-74**	89.9	-	-	87.7
**75-99**	72.9	-	-	72.8

Appendix Table 7. One-year survival by age and sex, for each cancer: adults diagnosed in England and Wales during 2005-06

Age group (years)	Bladder Men	Bladder Women	Brain Men	Brain Women	Breast Men	Breast Women	Cervix Men	Cervix Women	Colon Men	Colon Women
**15-44**	90.8	82.3	78.6	80.7	-	97.7	-	94.5	82.2	84.6
**45-54**	88.6	79.9	53.5	57.4	-	97.9	-	89.1	80.6	82.1
**55-64**	85.9	76.8	32.7	34.5	-	97.2	-	83.2	79.3	80.1
**65-74**	81.4	71.1	16.7	16.1	-	95.4	-	73.5	75.0	75.1
**75-99**	69.4	53.9	6.0	6.1	-	86.7	-	50.4	62.9	58.7

One-year survival

Age group (years)	Hodgkin's lymphoma Men	Hodgkin's lymphoma Women	Kidney Men	Kidney Women	Larynx Men	Larynx Women	Leukaemia Men	Leukaemia Women	Lung Men	Lung Women
**15-44**	97.7	98.3	84.3	85.6	90.0	-	83.4	80.0	44.3	48.5
**45-54**	92.8	94.1	79.1	81.5	89.0	-	81.7	78.5	37.5	42.6
**55-64**	84.8	87.7	74.6	76.7	87.6	-	77.9	76.1	32.6	37.1
**65-74**	70.6	77.1	68.9	68.7	84.9	-	68.8	67.8	28.4	31.0
**75-99**	50.6	57.8	55.3	51.8	75.6	-	52.3	49.2	20.0	21.7

One-year survival

Age group (years)	Melanoma Men	Melanoma Women	Myeloma Men	Myeloma Women	Non-Hodgkin lymphoma Men	Non-Hodgkin lymphoma Women	Oesophagus Men	Oesophagus Women	Others Men	Others Women
**15-44**	96.9	98.8	90.7	91.3	87.6	90.3	49.8	57.4	83.2	90.8
**45-54**	96.2	98.2	87.4	87.7	85.5	89.1	48.3	53.6	75.2	81.7
**55-64**	95.5	97.6	82.6	82.9	80.7	85.2	46.3	49.6	65.1	71.5
**65-74**	94.3	96.6	74.0	74.9	72.4	76.1	40.8	42.0	51.7	57.6
**75-99**	91.8	93.1	56.5	55.6	57.8	58.1	28.7	23.7	38.2	38.5

One-year survival

Age group (years)	Ovary Men	Ovary Women	Pancreas Men	Pancreas Women	Prostate Men	Prostate Women	Rectum Men	Rectum Women	Stomach Men	Stomach Women
**15-44**	-	92.8	33.4	42.8	98.6	-	86.2	89.4	55.5	55.8
**45-54**	-	86.1	26.3	30.8	98.2	-	86.6	89.2	52.2	53.0
**55-64**	-	77.6	21.5	23.9	97.6	-	85.2	87.5	47.7	48.7
**65-74**	-	64.4	16.6	17.7	96.1	-	80.3	82.1	41.1	41.9
**75-99**	-	41.1	10.1	9.0	88.2	-	68.6	65.8	30.0	27.4

One-year survival

Age group (years)	Testis Men	Testis Women	Uterus Men	Uterus Women
**15-44**	98.7	-	-	94.7
**45-54**	97.9	-	-	95.0
**55-64**	96.0	-	-	93.9
**65-74**	91.0	-	-	89.8
**75-99**	74.6	-	-	76.9

Appendix Table 7. One-year survival by age and sex, for each cancer: adults diagnosed in England and Wales during 2010-11

Age group (years)	Bladder Men	Bladder Women	Brain Men	Brain Women	Breast Men	Breast Women	Cervix Men	Cervix Women	Colon Men	Colon Women
**15-44**	89.8	80.0	83.4	84.7	-	98.3	-	95.3	83.9	85.9
**45-54**	88.0	78.4	59.7	63.4	-	98.4	-	90.0	82.7	83.7
**55-64**	85.3	75.4	37.7	39.5	-	97.9	-	83.8	81.4	81.8
**65-74**	80.9	69.8	21.2	20.1	-	96.5	-	74.6	77.6	77.2
**75-99**	68.5	52.4	7.6	7.4	-	88.9	-	50.7	65.3	60.6

Age group (years)	Hodgkin's lymphoma Men	Hodgkin's lymphoma Women	Kidney Men	Kidney Women	Larynx Men	Larynx Women	Leukaemia Men	Leukaemia Women	Lung Men	Lung Women
**15-44**	98.3	98.6	87.6	89.3	90.1	-	86.6	83.3	49.3	55.1
**45-54**	93.9	95.3	83.4	85.8	89.0	-	84.9	81.7	41.6	49.0
**55-64**	86.7	89.2	79.3	81.4	87.7	-	81.0	79.0	36.6	43.6
**65-74**	74.5	79.2	74.5	74.7	85.2	-	72.6	71.4	33.2	37.9
**75-99**	54.4	60.9	61.0	58.3	76.2	-	55.9	52.5	24.3	27.1

Age group (years)	Melanoma Men	Melanoma Women	Myeloma Men	Myeloma Women	Non-Hodgkin lymphoma Men	Non-Hodgkin lymphoma Women	Oesophagus Men	Oesophagus Women	Others Men	Others Women
**15-44**	97.8	99.2	93.3	94.1	90.0	92.4	53.5	61.7	86.5	92.7
**45-54**	97.2	98.8	91.0	91.4	88.1	91.4	52.9	58.1	79.7	84.9
**55-64**	96.6	98.3	87.2	87.2	83.6	87.8	51.0	53.8	69.9	75.2
**65-74**	95.8	97.6	80.2	80.6	76.4	80.0	45.6	46.1	57.1	62.2
**75-99**	93.8	95.0	64.0	62.0	61.9	62.1	32.5	26.1	42.1	42.0

Age group (years)	Ovary Men	Ovary Women	Pancreas Men	Pancreas Women	Prostate Men	Prostate Women	Rectum Men	Rectum Women	Stomach Men	Stomach Women
**15-44**	-	94.6	38.2	47.2	99.2	-	88.1	90.7	58.6	58.7
**45-54**	-	89.2	31.1	36.1	99.1	-	88.6	90.6	55.5	56.1
**55-64**	-	81.3	25.3	28.2	98.6	-	87.2	89.0	51.2	51.8
**65-74**	-	69.5	19.9	21.2	97.6	-	83.0	84.2	44.7	44.9
**75-99**	-	45.2	12.0	10.7	91.5	-	71.6	68.0	32.9	29.9

Age group (years)	Testis Men	Testis Women	Uterus Men	Uterus Women
**15-44**	98.9	-	-	94.9
**45-54**	98.2	-	-	95.5
**55-64**	96.2	-	-	94.4
**65-74**	91.6	-	-	90.9
**75-99**	74.4	-	-	79.3

All values: One-year survival

Appendix Table 7. One-year survival by age and sex, for each cancer: adults diagnosed in England and Wales during 2015-16

Age group (years)	Bladder		Brain		Breast		Cervix		Colon	
	Men	Women	Men	Women	Men	Women	Men	Women	Men	Women
**15-44**	88.4	77.7	86.4	88.7	-	98.5	-	95.9	85.2	86.7
**45-54**	87.2	76.7	65.3	69.6	-	98.7	-	90.8	84.3	84.8
**55-64**	84.9	74.2	44.7	46.8	-	98.3	-	84.6	83.3	83.2
**65-74**	80.4	68.8	26.5	25.0	-	97.1	-	75.2	79.6	78.6
**75-99**	67.6	50.7	9.9	9.5	-	89.9	-	52.1	66.7	61.3

Age group (years)	Hodgkin's lymphoma		Kidney		Larynx		Leukaemia		Lung	
	Men	Women	Men	Women	Men	Women	Men	Women	Men	Women
**15-44**	98.7	98.9	90.7	92.1	90.1	-	89.1	86.2	54.6	61.8
**45-54**	95.0	95.6	87.3	89.4	88.8	-	87.4	84.5	46.1	56.5
**55-64**	88.7	90.1	83.9	85.9	87.5	-	83.9	81.9	41.3	51.6
**65-74**	77.7	80.8	79.9	80.2	85.1	-	75.9	74.6	38.6	46.1
**75-99**	57.5	63.0	67.6	64.9	76.2	-	59.0	56.3	29.9	35.1

Age group (years)	Melanoma		Myeloma		Non-Hodgkin lymphoma		Oesophagus		Others	
	Men	Women	Men	Women	Men	Women	Men	Women	Men	Women
**15-44**	98.5	99.5	95.3	96.1	92.0	94.0	56.0	64.7	89.7	94.6
**45-54**	98.1	99.3	93.8	94.2	90.2	93.1	56.2	61.9	83.8	87.9
**55-64**	97.7	99.0	91.3	91.3	86.4	90.1	54.6	57.7	75.5	79.6
**65-74**	97.1	98.6	85.8	85.9	79.9	82.9	49.0	50.0	63.1	67.3
**75-99**	95.7	96.8	71.8	69.4	66.1	66.3	35.4	28.9	47.3	46.7

Age group (years)	Ovary		Pancreas		Prostate		Rectum		Stomach	
	Men	Women	Men	Women	Men	Women	Men	Women	Men	Women
**15-44**	-	96.2	44.3	54.4	99.4	-	89.4	91.6	61.4	61.5
**45-54**	-	91.4	36.5	42.1	99.3	-	90.0	91.6	58.4	58.7
**55-64**	-	85.2	30.3	33.8	98.9	-	88.9	90.2	54.4	54.8
**65-74**	-	73.9	24.0	25.5	97.9	-	84.9	85.5	48.1	48.0
**75-99**	-	50.1	14.5	13.1	91.7	-	74.0	69.3	35.8	32.4

Age group (years)	Testis		Uterus	
	Men	Women	Men	Women
**15-44**	99.0	-	-	94.8
**45-54**	98.1	-	-	95.6
**55-64**	96.4	-	-	94.7
**65-74**	91.4	-	-	91.2
**75-99**	71.4	-	-	79.9

(All values: One-year survival)

Appendix Table 7. One-year survival by age and sex, for each cancer: adults diagnosed in England and Wales during 2018

Age group (years)	Bladder Men	Bladder Women	Brain Men	Brain Women	Breast Men	Breast Women	Cervix Men	Cervix Women	Colon Men	Colon Women
**15-44**	87.8	76.5	88.0	90.1	-	98.6	-	96.1	85.6	86.7
**45-54**	86.8	75.9	67.5	71.9	-	98.8	-	91.0	85.0	85.2
**55-64**	84.6	73.5	48.0	50.4	-	98.4	-	85.3	84.0	83.7
**65-74**	80.0	67.7	28.7	28.1	-	97.2	-	74.9	80.3	79.0
**75-99**	67.1	50.9	11.0	10.7	-	90.6	-	49.9	67.6	62.4

One-year survival

Age group (years)	Hodgkin's lymphoma Men	Hodgkin's lymphoma Women	Kidney Men	Kidney Women	Larynx Men	Larynx Women	Leukaemia Men	Leukaemia Women	Lung Men	Lung Women
**15-44**	98.9	99.1	92.0	93.3	89.9	-	90.2	87.4	57.3	65.1
**45-54**	95.6	95.9	88.9	90.8	88.7	-	88.5	85.8	48.4	60.3
**55-64**	89.7	91.1	86.0	87.5	87.4	-	85.2	83.3	43.8	55.6
**65-74**	78.9	81.9	82.2	82.1	85.0	-	76.9	75.9	41.3	50.1
**75-99**	58.2	62.8	70.9	67.8	76.4	-	60.5	57.9	33.0	39.7

One-year survival

Age group (years)	Melanoma Men	Melanoma Women	Myeloma Men	Myeloma Women	Non-Hodgkin lymphoma Men	Non-Hodgkin lymphoma Women	Oesophagus Men	Oesophagus Women	Others Men	Others Women
**15-44**	98.8	99.7	96.3	96.9	92.9	94.7	56.9	67.0	90.9	95.1
**45-54**	98.5	99.5	95.0	95.2	91.1	93.8	57.5	63.7	85.6	89.2
**55-64**	98.2	99.2	92.8	92.6	87.7	91.0	56.1	59.5	78.0	81.6
**65-74**	97.7	98.9	87.9	87.9	81.2	84.0	50.4	51.4	65.6	69.3
**75-99**	96.4	97.6	75.5	74.4	68.3	68.2	36.7	30.7	49.8	49.1

One-year survival

Age group (years)	Ovary Men	Ovary Women	Pancreas Men	Pancreas Women	Prostate Men	Prostate Women	Rectum Men	Rectum Women	Stomach Men	Stomach Women
**15-44**	-	96.7	47.4	57.8	99.5	-	90.0	92.0	62.9	62.7
**45-54**	-	92.4	39.5	45.2	99.3	-	90.6	92.0	59.8	60.1
**55-64**	-	86.7	33.2	36.8	99.0	-	89.6	90.6	55.9	56.2
**65-74**	-	75.7	26.0	27.5	97.8	-	85.7	86.0	49.6	49.3
**75-99**	-	53.0	15.9	14.5	91.5	-	74.9	70.3	37.3	33.8

One-year survival

Age group (years)	Testis Men	Testis Women	Uterus Men	Uterus Women
**15-44**	99.1	-	-	94.5
**45-54**	98.1	-	-	95.5
**55-64**	96.4	-	-	94.7
**65-74**	90.9	-	-	91.1
**75-99**	74.2	-	-	79.9

Appendix Table 8. Five-year survival by age and sex, for each cancer: adults diagnosed in England and Wales during 1971-72

Age group (years)	Bladder		Brain		Breast		Cervix		Colon	
	Men	Women	Men	Women	Men	Women	Men	Women	Men	Women
**15-44**	82.3	70.6	26.6	28.7	-	61.4	-	66.0	35.0	39.8
**45-54**	67.2	62.2	8.9	10.5	-	59.4	-	56.3	26.2	27.3
**55-64**	54.3	54.0	3.6	4.3	-	53.2	-	49.0	25.7	26.2
**65-74**	44.6	44.8	1.8	2.1	-	50.7	-	41.2	25.4	25.6
**75-99**	30.0	26.6	1.3	1.9	-	44.6	-	28.5	20.9	18.1

Age group (years)	HL		Kidney		Larynx		Leukaemia		Lung	
	Men	Women	Men	Women	Men	Women	Men	Women	Men	Women
**15-44**	69.3	74.8	39.0	38.0	71.9	-	18.2	19.2	13.6	11.5
**45-54**	52.0	57.0	32.7	32.8	68.1	-	21.8	21.0	12.5	7.8
**55-64**	34.7	39.7	29.1	29.3	63.7	-	21.0	22.0	9.0	5.4
**65-74**	19.9	26.0	27.3	25.6	58.4	-	16.2	18.7	4.9	3.8
**75-99**	10.1	13.6	21.1	18.6	49.4	-	10.1	12.3	1.7	2.0

Age group (years)	Melanoma		Myeloma		NHL		Oesophagus		Other	
	Men	Women	Men	Women	Men	Women	Men	Women	Men	Women
**15-44**	47.4	72.2	36.2	23.8	44.5	47.7	11.1	12.2	42.8	53.1
**45-54**	42.6	67.3	21.3	18.9	40.6	44.4	7.1	9.9	32.1	35.7
**55-64**	39.9	63.1	15.8	16.2	33.5	38.4	5.8	8.6	26.2	26.4
**65-74**	37.4	56.8	13.7	14.0	25.9	29.8	4.9	7.2	23.5	20.3
**75-99**	32.1	42.4	10.7	10.5	17.8	20.7	2.7	3.5	26.5	17.4

Age group (years)	Ovary		Pancreas		Prostate		Rectum		Stomach	
	Men	Women	Men	Women	Men	Women	Men	Women	Men	Women
**15-44**	-	41.0	5.9	7.9	9.7	-	34.6	35.7	11.6	11.3
**45-54**	-	24.9	3.8	4.0	19.1	-	30.2	32.4	9.5	9.7
**55-64**	-	18.2	3.1	3.0	28.3	-	29.9	32.0	7.5	8.0
**65-74**	-	15.3	2.9	2.9	36.9	-	29.5	30.4	5.6	6.3
**75-99**	-	12.5	2.9	2.9	31.8	-	23.5	20.1	3.1	3.3

Age group (years)	Testis		Uterus	
	Men	Women	Men	Women
**15-44**	64.6	-	-	82.2
**45-54**	67.9	-	-	77.7
**55-64**	58.8	-	-	69.6
**65-74**	44.7	-	-	55.6
**75-99**	25.3	-	-	35.8

(All tables: Five-year survival)

Appendix Table 8. Five-year survival by age and sex, for each cancer: adults diagnosed in England and Wales during 1980-81

Age group (years)	Five-year survival: Bladder		Brain		Breast		Cervix		Colon	
	Men	Women	Men	Women	Men	Women	Men	Women	Men	Women
**15-44**	85.5	74.8	32.0	35.4	-	64.5	-	71.3	40.7	44.7
**45-54**	73.9	67.9	10.2	13.6	-	65.8	-	58.3	33.0	34.7
**55-64**	64.9	62.4	4.0	5.3	-	61.6	-	49.6	32.9	34.0
**65-74**	56.5	54.3	1.7	2.3	-	57.3	-	40.6	31.9	32.9
**75-99**	44.1	36.4	1.0	1.7	-	46.7	-	26.6	26.4	23.5

Age group (years)	Five-year survival: HL		Kidney		Larynx		Leukaemia		Lung	
	Men	Women	Men	Women	Men	Women	Men	Women	Men	Women
**15-44**	79.0	82.3	46.5	45.4	71.8	-	28.7	29.9	14.4	12.4
**45-54**	63.1	66.3	38.2	38.8	66.9	-	31.4	30.8	12.1	8.4
**55-64**	45.3	50.6	33.8	34.0	63.0	-	29.1	30.5	8.9	6.1
**65-74**	27.3	33.0	30.7	28.3	58.2	-	21.5	24.9	5.0	4.2
**75-99**	15.2	18.1	23.5	19.6	49.8	-	12.9	15.7	2.0	2.2

Age group (years)	Five-year survival: Melanoma		Myeloma		NHL		Oesophagus		Other	
	Men	Women	Men	Women	Men	Women	Men	Women	Men	Women
**15-44**	62.0	82.0	38.6	34.5	51.5	55.0	9.5	13.5	47.1	63.0
**45-54**	57.3	77.3	26.1	26.3	46.1	51.3	6.6	10.8	35.0	43.7
**55-64**	53.9	73.8	19.6	21.5	38.6	44.6	5.7	9.2	27.5	32.8
**65-74**	50.7	68.0	14.9	16.6	29.2	33.5	4.6	7.3	22.3	24.0
**75-99**	44.3	54.4	10.0	10.6	20.0	21.9	2.5	3.1	22.3	18.7

Age group (years)	Five-year survival: Ovary		Pancreas		Prostate		Rectum		Stomach	
	Men	Women	Men	Women	Men	Women	Men	Women	Men	Women
**15-44**	-	50.0	6.4	9.3	25.6	-	37.2	41.4	13.8	14.9
**45-54**	-	30.5	3.8	4.1	34.7	-	34.4	38.9	11.5	12.8
**55-64**	-	21.8	2.9	2.8	40.4	-	34.1	38.3	9.4	11.0
**65-74**	-	16.6	2.5	2.3	43.7	-	32.5	35.0	7.0	8.6
**75-99**	-	11.9	2.1	1.8	32.7	-	25.1	22.6	4.2	4.5

Age group (years)	Five-year survival: Testis		Uterus	
	Men	Women	Men	Women
**15-44**	83.2	-	-	82.4
**45-54**	82.8	-	-	79.0
**55-64**	76.3	-	-	72.7
**65-74**	63.3	-	-	59.3
**75-99**	41.1	-	-	39.6

Appendix Table 8. Five-year survival by age and sex, for each cancer: adults diagnosed in England and Wales during 1990-91

Age group (years)	Bladder Men	Bladder Women	Brain Men	Brain Women	Breast Men	Breast Women	Cervix Men	Cervix Women	Colon Men	Colon Women
**15-44**	85.3	75.1	39.5	44.0	-	71.8	-	76.7	47.5	51.9
**45-54**	77.2	70.4	13.4	18.6	-	75.6	-	63.7	42.1	44.7
**55-64**	69.9	65.4	4.8	7.2	-	73.1	-	53.0	42.3	44.2
**65-74**	63.3	59.0	1.9	2.6	-	68.1	-	43.1	40.7	42.6
**75-99**	51.1	41.0	0.9	1.5	-	53.5	-	27.5	33.3	31.0

Age group (years)	HL Men	HL Women	Kidney Men	Kidney Women	Larynx Men	Larynx Women	Leukaemia Men	Leukaemia Women	Lung Men	Lung Women
**15-44**	86.7	88.7	54.3	54.8	72.4	-	43.4	43.3	15.0	14.1
**45-54**	73.5	75.8	45.8	47.9	67.9	-	44.0	42.6	11.7	10.2
**55-64**	57.6	59.3	40.4	41.2	63.9	-	39.6	40.8	8.4	7.2
**65-74**	38.8	42.6	36.2	33.8	60.3	-	29.9	33.3	5.4	5.1
**75-99**	21.4	24.2	26.4	21.5	53.4	-	17.0	19.8	2.3	2.6

Age group (years)	Melanoma Men	Melanoma Women	Myeloma Men	Myeloma Women	NHL Men	NHL Women	Oesophagus Men	Oesophagus Women	Other Men	Other Women
**15-44**	75.2	89.1	44.5	46.9	60.7	64.6	9.2	16.2	52.3	69.9
**45-54**	71.2	85.5	35.1	38.0	54.9	61.0	7.8	13.1	40.3	52.6
**55-64**	67.7	82.4	26.6	29.4	46.2	53.4	6.9	11.0	30.3	39.4
**65-74**	64.1	78.1	19.0	21.2	36.1	40.9	5.5	8.3	22.5	28.6
**75-99**	56.6	65.6	10.6	11.6	24.4	26.0	2.8	3.1	19.5	20.0

Age group (years)	Ovary Men	Ovary Women	Pancreas Men	Pancreas Women	Prostate Men	Prostate Women	Rectum Men	Rectum Women	Stomach Men	Stomach Women
**15-44**	-	62.0	7.5	11.8	54.9	-	43.9	50.8	18.1	20.3
**45-54**	-	40.4	4.5	5.1	58.8	-	42.8	49.7	15.6	18.2
**55-64**	-	28.5	3.1	3.0	60.1	-	42.5	48.8	13.0	15.6
**65-74**	-	20.8	2.3	2.1	57.7	-	39.7	44.3	10.2	12.6
**75-99**	-	13.0	1.7	1.3	39.8	-	30.1	28.5	6.2	6.6

Age group (years)	Testis Men	Testis Women	Uterus Men	Uterus Women
**15-44**	92.9	-	-	83.2
**45-54**	91.7	-	-	81.7
**55-64**	87.2	-	-	76.4
**65-74**	77.9	-	-	65.0
**75-99**	56.2	-	-	44.7

(All values: five-year survival)

Appendix Table 8. Five-year survival by age and sex, for each cancer: adults diagnosed in England and Wales during 2000-01

Age group (years)	Bladder Men	Bladder Women	Brain Men	Brain Women	Breast Men	Breast Women	Cervix Men	Cervix Women	Colon Men	Colon Women
**15-44**	82.5	69.6	48.3	53.6	-	82.3	-	82.7	57.0	61.4
**45-54**	74.9	66.4	17.9	23.9	-	86.7	-	69.4	53.1	55.5
**55-64**	68.9	62.5	6.7	9.5	-	85.7	-	58.9	53.4	55.4
**65-74**	62.2	55.6	2.6	3.3	-	81.2	-	46.8	51.1	53.1
**75-99**	50.3	37.4	1.2	1.5	-	66.2	-	29.6	42.8	40.5

Five-year survival

Age group (years)	HL Men	HL Women	Kidney Men	Kidney Women	Larynx Men	Larynx Women	Leukaemia Men	Leukaemia Women	Lung Men	Lung Women
**15-44**	91.5	92.7	64.7	67.4	75.2	-	59.3	56.7	17.4	17.9
**45-54**	81.4	82.5	55.9	60.4	69.9	-	57.9	54.9	12.5	13.6
**55-64**	67.8	68.1	49.9	53.4	66.8	-	52.6	52.1	9.2	10.3
**65-74**	49.2	52.0	44.5	43.9	64.0	-	40.3	42.3	6.5	7.2
**75-99**	29.9	30.1	32.6	28.4	58.0	-	23.9	25.6	3.2	3.9

Five-year survival

Age group (years)	Melanoma Men	Melanoma Women	Myeloma Men	Myeloma Women	NHL Men	NHL Women	Oesophagus Men	Oesophagus Women	Other Men	Other Women
**15-44**	84.8	93.6	57.9	63.4	72.4	76.4	13.4	21.7	60.6	76.8
**45-54**	81.7	90.8	49.7	52.1	66.4	73.0	12.7	18.2	48.3	60.2
**55-64**	78.8	88.8	40.8	41.8	58.5	66.3	12.0	15.5	36.9	47.1
**65-74**	75.2	85.3	28.9	29.8	47.6	53.3	9.4	11.2	25.5	32.9
**75-99**	67.5	75.3	16.1	15.1	33.8	35.2	5.0	4.1	19.4	21.2

Five-year survival

Age group (years)	Ovary Men	Ovary Women	Pancreas Men	Pancreas Women	Prostate Men	Prostate Women	Rectum Men	Rectum Women	Stomach Men	Stomach Women
**15-44**	-	75.0	11.4	16.0	83.5	-	55.7	63.2	23.7	26.6
**45-54**	-	53.8	6.4	7.6	84.1	-	56.1	63.1	21.1	24.3
**55-64**	-	40.5	4.2	4.6	82.9	-	55.7	62.1	18.3	21.5
**65-74**	-	29.4	2.9	2.8	78.7	-	51.8	56.4	14.6	17.3
**75-99**	-	17.5	1.8	1.5	60.4	-	40.7	39.0	9.3	9.6

Five-year survival

Age group (years)	Testis Men	Testis Women	Uterus Men	Uterus Women
**15-44**	96.5	-	-	85.5
**45-54**	95.3	-	-	85.3
**55-64**	92.3	-	-	81.6
**65-74**	85.0	-	-	72.1
**75-99**	65.2	-	-	53.6

Appendix Table 8. Five-year survival by age and sex, for each cancer: adults diagnosed in England and Wales during 2005-06

Age group (years)	Bladder Men	Bladder Women	Brain Men	Brain Women	Breast Men	Breast Women	Cervix Men	Cervix Women	Colon Men	Colon Women
**15-44**	79.7	65.8	53.2	58.6	-	86.3	-	85.1	61.1	65.1
**45-54**	73.1	63.5	21.7	28.2	-	90.2	-	71.5	57.8	59.6
**55-64**	67.3	60.0	8.7	11.8	-	89.6	-	60.9	58.2	59.6
**65-74**	60.8	53.3	3.3	4.0	-	86.0	-	48.5	55.6	57.1
**75-99**	48.5	34.7	1.4	1.7	-	71.3	-	27.7	46.8	44.1

Age group (years)	HL Men	HL Women	Kidney Men	Kidney Women	Larynx Men	Larynx Women	Leukaemia Men	Leukaemia Women	Lung Men	Lung Women
**15-44**	93.3	94.4	70.1	73.9	74.7	-	66.3	63.0	19.8	21.5
**45-54**	85.1	85.8	61.8	67.7	70.0	-	64.4	60.9	14.1	16.9
**55-64**	72.5	73.9	55.8	60.9	67.0	-	58.9	57.7	10.7	13.3
**65-74**	53.6	57.4	49.9	50.9	64.8	-	46.1	47.5	8.1	9.7
**75-99**	33.3	33.8	36.7	33.5	59.3	-	27.5	28.9	4.2	5.2

Age group (years)	Melanoma Men	Melanoma Women	Myeloma Men	Myeloma Women	NHL Men	NHL Women	Oesophagus Men	Oesophagus Women	Other Men	Other Women
**15-44**	88.4	95.2	66.1	71.0	77.8	81.7	16.6	25.4	65.3	80.1
**45-54**	85.9	92.9	59.1	61.1	72.7	78.8	16.4	21.9	53.8	65.0
**55-64**	83.3	91.1	50.2	50.1	65.2	72.8	15.8	18.9	41.5	51.3
**65-74**	79.9	88.3	37.1	36.9	54.6	60.7	12.8	13.9	28.6	36.4
**75-99**	72.7	79.3	21.4	19.5	40.5	41.5	6.9	5.2	20.5	22.5

Age group (years)	Ovary Men	Ovary Women	Pancreas Men	Pancreas Women	Prostate Men	Prostate Women	Rectum Men	Rectum Women	Stomach Men	Stomach Women
**15-44**	-	79.9	14.0	21.5	91.9	-	60.7	67.8	26.1	29.6
**45-54**	-	60.7	8.1	10.4	91.9	-	61.7	68.0	23.7	27.4
**55-64**	-	46.3	5.3	6.0	90.8	-	61.3	67.0	20.8	24.3
**65-74**	-	34.2	3.5	3.6	87.4	-	57.1	61.0	16.8	19.7
**75-99**	-	20.0	2.1	1.7	72.2	-	45.1	42.9	11.0	11.0

Age group (years)	Testis Men	Testis Women	Uterus Men	Uterus Women
**15-44**	97.3	-	-	86.2
**45-54**	96.2	-	-	86.7
**55-64**	93.3	-	-	83.7
**65-74**	86.3	-	-	75.0
**75-99**	66.8	-	-	57.6

(All values: Five-year survival)

Appendix Table 8. Five-year survival by age and sex, for each cancer: adults diagnosed in England and Wales during 2010-11

Age group (years)	Bladder		Brain		Breast		Cervix		Colon	
	Men	Women	Men	Women	Men	Women	Men	Women	Men	Women
**15-44**	77.0	61.7	60.3	64.3	-	88.1	-	86.9	63.7	67.7
**45-54**	71.1	60.5	26.0	33.2	-	92.0	-	73.3	61.0	62.1
**55-64**	65.4	57.3	10.5	14.1	-	91.6	-	61.8	61.4	62.2
**65-74**	59.1	50.8	4.4	5.2	-	88.5	-	49.7	58.8	59.7
**75-99**	46.5	32.2	1.7	2.0	-	73.4	-	27.7	49.0	45.8

Five-year survival

Age group (years)	HL		Kidney		Larynx		Leukaemia		Lung	
	Men	Women	Men	Women	Men	Women	Men	Women	Men	Women
**15-44**	94.7	95.7	75.1	79.7	74.5	-	72.1	68.9	23.8	27.5
**45-54**	87.2	89.0	67.8	74.0	69.1	-	69.9	66.5	16.8	22.1
**55-64**	75.5	77.9	61.6	67.2	66.0	-	63.7	62.7	13.0	17.8
**65-74**	58.4	62.3	55.9	57.8	64.2	-	51.3	52.7	10.6	13.7
**75-99**	37.0	39.1	41.3	38.9	59.1	-	31.0	32.5	5.9	7.5

Five-year survival

Age group (years)	Melanoma		Myeloma		NHL		Oesophagus		Other	
	Men	Women	Men	Women	Men	Women	Men	Women	Men	Women
**15-44**	91.4	96.4	73.4	79.3	82.1	85.8	18.9	30.3	70.4	83.4
**45-54**	89.4	94.7	68.1	70.7	77.5	83.4	19.6	26.5	59.7	69.6
**55-64**	87.1	93.2	59.7	59.5	70.3	77.8	19.0	22.8	46.4	55.5
**65-74**	84.3	91.0	46.3	46.3	60.8	67.0	15.6	17.1	32.8	40.5
**75-99**	77.7	83.3	28.0	25.5	46.0	47.2	8.4	6.4	22.5	24.7

Five-year survival

Age group (years)	Ovary		Pancreas		Prostate		Rectum		Stomach	
	Men	Women	Men	Women	Men	Women	Men	Women	Men	Women
**15-44**	-	83.6	16.8	24.7	95.6	-	63.8	70.4	28.5	32.4
**45-54**	-	66.0	10.3	13.4	95.4	-	65.3	71.1	26.2	30.3
**55-64**	-	50.9	6.7	7.9	94.3	-	64.8	69.8	23.1	27.0
**65-74**	-	38.3	4.4	4.8	91.6	-	60.4	64.0	19.0	22.0
**75-99**	-	21.8	2.5	2.1	78.6	-	47.5	44.7	12.4	12.5

Five-year survival

Age group (years)	Testis		Uterus	
	Men	Women	Men	Women
**15-44**	97.7	-	-	86.3
**45-54**	96.6	-	-	87.4
**55-64**	93.6	-	-	84.4
**65-74**	87.0	-	-	76.5
**75-99**	65.6	-	-	60.0

Appendix Table 8. Five-year survival by age and sex, for each cancer: adults diagnosed in England and Wales during 2015-16

Age group (years)	Bladder Men	Bladder Women	Brain Men	Brain Women	Breast Men	Breast Women	Cervix Men	Cervix Women	Colon Men	Colon Women
**15-44**	73.5	57.4	64.3	70.6	-	88.4	-	88.5	65.2	69.0
**45-54**	68.7	57.4	30.2	39.0	-	93.0	-	74.8	62.9	63.6
**55-64**	63.7	54.8	13.5	18.1	-	92.7	-	63.0	63.4	63.8
**65-74**	57.3	48.6	5.7	6.7	-	89.5	-	50.2	60.5	61.1
**75-99**	44.5	29.5	2.2	2.5	-	73.6	-	28.4	49.6	46.2

Age group (years)	HL Men	HL Women	Kidney Men	Kidney Women	Larynx Men	Larynx Women	Leukaemia Men	Leukaemia Women	Lung Men	Lung Women
**15-44**	95.6	96.9	80.0	84.1	73.7	-	76.9	74.2	28.7	34.8
**45-54**	88.8	90.5	73.4	79.4	67.0	-	74.3	71.6	20.2	29.1
**55-64**	77.9	81.1	67.8	73.4	64.1	-	68.5	67.7	16.1	24.4
**65-74**	61.6	67.1	62.3	64.4	62.7	-	55.8	57.7	13.9	19.6
**75-99**	39.1	44.7	46.9	44.7	57.9	-	34.2	36.8	8.5	11.8

Age group (years)	Melanoma Men	Melanoma Women	Myeloma Men	Myeloma Women	NHL Men	NHL Women	Oesophagus Men	Oesophagus Women	Other Men	Other Women
**15-44**	94.1	97.5	79.5	85.7	85.4	88.7	20.2	34.4	75.6	86.7
**45-54**	92.6	96.2	75.7	78.8	81.0	86.5	21.8	31.3	65.3	74.0
**55-64**	90.9	95.1	68.9	70.0	74.6	81.7	21.4	27.3	52.6	61.0
**65-74**	88.6	93.4	55.6	57.0	65.5	71.5	17.5	20.9	37.6	45.4
**75-99**	82.9	87.3	35.6	34.2	50.7	52.2	9.4	8.1	25.2	27.8

Age group (years)	Ovary Men	Ovary Women	Pancreas Men	Pancreas Women	Prostate Men	Prostate Women	Rectum Men	Rectum Women	Stomach Men	Stomach Women
**15-44**	-	87.2	21.0	31.6	96.6	-	65.4	71.9	30.8	35.3
**45-54**	-	69.9	13.1	17.4	96.3	-	67.6	73.0	28.4	33.0
**55-64**	-	55.9	8.7	10.6	95.3	-	67.1	71.8	25.4	29.8
**65-74**	-	41.7	5.7	6.4	92.4	-	62.3	65.5	21.1	24.6
**75-99**	-	23.6	3.1	2.7	78.2	-	48.4	45.0	13.9	14.1

Age group (years)	Testis Men	Testis Women	Uterus Men	Uterus Women
**15-44**	97.9	-	-	85.7
**45-54**	96.5	-	-	87.4
**55-64**	93.7	-	-	84.8
**65-74**	86.3	-	-	76.9
**75-99**	61.5	-	-	60.5

All values: Five-year survival

Appendix Table 8. Five-year survival by age and sex, for each cancer: adults diagnosed in England and Wales during 2018

Age group	Five-year survival									
	Bladder		Brain		Breast		Cervix		Colon	
(years)	Men	Women	Men	Women	Men	Women	Men	Women	Men	Women
**15-44**	72.1	55.3	66.8	72.9	-	88.2	-	88.8	64.8	67.8
**45-54**	67.5	55.8	32.0	41.4	-	93.3	-	75.2	63.7	64.2
**55-64**	62.8	53.5	15.1	20.4	-	93.1	-	64.1	64.2	64.4
**65-74**	56.1	46.8	6.3	7.8	-	89.6	-	49.7	61.0	61.5
**75-99**	43.6	29.1	2.4	2.8	-	73.9	-	26.7	49.9	46.9

Age group	Five-year survival									
	HL		Kidney		Larynx		Leukaemia		Lung	
(years)	Men	Women	Men	Women	Men	Women	Men	Women	Men	Women
**15-44**	96.2	97.3	82.0	86.1	72.1	-	78.9	76.6	31.4	38.7
**45-54**	89.7	91.6	75.9	81.5	66.1	-	76.2	74.0	22.0	33.1
**55-64**	79.2	83.5	70.7	75.8	63.0	-	70.6	70.2	17.8	28.2
**65-74**	62.7	69.9	65.1	66.9	61.8	-	57.3	59.9	15.8	22.9
**75-99**	39.1	46.2	50.1	47.3	57.3	-	35.8	38.8	10.2	14.6

Age group	Five-year survival									
	Melanoma		Myeloma		NHL		Oesophagus		Other	
(years)	Men	Women	Men	Women	Men	Women	Men	Women	Men	Women
**15-44**	95.2	98.0	82.6	88.6	86.8	89.8	20.4	37.9	77.8	87.8
**45-54**	93.9	96.8	79.1	82.2	82.4	87.8	22.7	34.0	67.8	76.1
**55-64**	92.5	95.9	72.7	73.9	76.6	83.2	22.4	29.7	55.6	63.6
**65-74**	90.4	94.3	59.5	61.5	67.3	73.0	18.2	22.6	39.7	47.3
**75-99**	85.2	89.2	39.5	40.4	53.0	54.3	9.9	9.3	26.6	29.5

Age group	Five-year survival									
	Ovary		Pancreas		Prostate		Rectum		Stomach	
(years)	Men	Women	Men	Women	Men	Women	Men	Women	Men	Women
**15-44**	-	88.2	23.3	34.9	96.9	-	66.0	72.5	32.1	36.7
**45-54**	-	71.8	14.8	19.6	96.5	-	68.5	73.8	29.6	34.5
**55-64**	-	57.9	10.0	12.3	95.4	-	68.0	72.5	26.6	31.2
**65-74**	-	43.1	6.3	7.1	92.0	-	62.9	65.9	22.2	25.7
**75-99**	-	24.8	3.4	3.1	77.2	-	48.4	45.5	14.7	15.0

Age group	Five-year survival			
	Testis		Uterus	
(years)	Men	Women	Men	Women
**15-44**	98.0	-	-	84.9
**45-54**	96.5	-	-	87.3
**55-64**	93.6	-	-	84.8
**65-74**	85.4	-	-	76.7
**75-99**	64.2	-	-	60.4

Appendix Table 9. Seven-year survival by age and sex, for each cancer: adults diagnosed in England and Wales during 1971-72

Age group (years)	Bladder		Brain		Breast		Cervix		Colon	
	Men	Women	Men	Women	Men	Women	Men	Women	Men	Women
**15-44**	81.2	68.0	23.0	25.1	-	54.4	-	64.1	32.2	37.3
**45-54**	64.8	59.3	7.6	8.6	-	52.1	-	53.3	23.9	25.0
**55-64**	51.2	50.8	3.3	3.8	-	46.4	-	45.6	24.0	24.3
**65-74**	41.6	41.6	1.9	2.0	-	45.4	-	37.9	24.2	24.4
**75-99**	28.0	23.7	1.6	2.0	-	42.0	-	26.1	20.9	18.2

Seven-year survival

Age group (years)	HL		Kidney		Larynx		Leukaemia		Lung	
	Men	Women	Men	Women	Men	Women	Men	Women	Men	Women
**15-44**	65.2	70.9	36.0	35.3	70.7	-	14.6	16.4	11.9	10.1
**45-54**	48.3	52.6	29.7	29.9	66.1	-	17.4	17.0	10.9	6.7
**55-64**	31.8	35.3	26.4	26.6	61.3	-	16.4	17.7	7.7	4.6
**65-74**	18.0	22.3	25.0	23.3	56.1	-	12.0	14.9	4.0	3.2
**75-99**	9.4	11.0	19.5	16.9	47.9	-	6.7	10.1	1.3	1.6

Seven-year survival

Age group (years)	Melanoma		Myeloma		NHL		Oesophagus		Other	
	Men	Women	Men	Women	Men	Women	Men	Women	Men	Women
**15-44**	42.6	68.3	29.9	19.0	41.7	44.8	10.3	11.0	40.5	50.8
**45-54**	38.4	63.3	16.3	14.2	36.3	40.0	6.5	8.8	30.1	33.4
**55-64**	36.3	59.4	12.1	12.0	28.9	33.6	5.3	7.8	24.7	24.6
**65-74**	34.6	53.5	11.0	10.6	21.8	25.2	4.6	6.6	22.5	19.0
**75-99**	30.6	39.7	10.1	8.9	14.8	17.3	2.5	3.2	26.4	16.9

Seven-year survival

Age group (years)	Ovary		Pancreas		Prostate		Rectum		Stomach	
	Men	Women	Men	Women	Men	Women	Men	Women	Men	Women
**15-44**	-	37.7	5.4	7.1	4.4	-	30.9	32.0	10.3	10.1
**45-54**	-	22.2	3.3	3.5	11.6	-	26.7	29.1	8.4	8.6
**55-64**	-	16.4	2.6	2.5	20.5	-	26.9	29.2	6.6	7.1
**65-74**	-	14.2	2.5	2.4	29.8	-	27.3	28.3	5.0	5.6
**75-99**	-	12.7	2.6	2.6	26.4	-	22.7	19.3	2.8	2.9

Seven-year survival

Age group (years)	Testis		Uterus	
	Men	Women	Men	Women
**15-44**	63.2	-	-	81.1
**45-54**	67.1	-	-	76.4
**55-64**	58.2	-	-	68.0
**65-74**	44.4	-	-	53.9
**75-99**	25.3	-	-	35.1

Appendix Table 9. Seven-year survival by age and sex, for each cancer: adults diagnosed in England and Wales during 1980-81

Age group (years)	Bladder		Brain		Breast		Cervix		Colon	
	**Men**	**Women**	**Men**	**Women**	**Men**	**Women**	**Men**	**Women**	**Men**	**Women**
**15-44**	84.1	72.5	27.4	30.7	-	57.5	-	69.4	37.4	41.7
**45-54**	71.1	65.2	8.4	11.1	-	59.1	-	54.8	30.0	31.8
**55-64**	61.2	59.5	3.5	4.6	-	55.3	-	45.6	30.4	31.4
**65-74**	52.7	51.3	1.7	2.1	-	52.0	-	36.7	29.9	31.0
**75-99**	40.8	33.3	1.3	1.8	-	43.3	-	23.6	25.7	23.1

Seven-year survival

Age group (years)	HL		Kidney		Larynx		Leukaemia		Lung	
	**Men**	**Women**	**Men**	**Women**	**Men**	**Women**	**Men**	**Women**	**Men**	**Women**
**15-44**	75.4	79.3	43.2	42.5	70.1	-	24.6	26.4	12.2	10.8
**45-54**	58.8	62.4	34.7	35.6	64.2	-	26.5	26.1	10.1	7.1
**55-64**	41.0	46.2	30.4	30.8	59.8	-	24.1	25.4	7.2	5.1
**65-74**	23.9	28.9	27.8	25.5	55.1	-	16.7	20.2	3.9	3.4
**75-99**	13.0	15.0	21.1	17.3	47.5	-	9.0	12.6	1.5	1.7

Seven-year survival

Age group (years)	Melanoma		Myeloma		NHL		Oesophagus		Other	
	**Men**	**Women**	**Men**	**Women**	**Men**	**Women**	**Men**	**Women**	**Men**	**Women**
**15-44**	57.4	78.9	31.4	28.1	48.3	51.7	8.3	11.8	44.2	60.5
**45-54**	52.9	73.8	19.5	19.8	41.4	46.5	5.8	9.4	32.2	40.7
**55-64**	49.9	70.4	14.2	15.5	33.5	39.4	5.0	8.0	25.2	30.2
**65-74**	47.3	64.7	10.8	11.7	24.4	28.4	4.1	6.4	20.6	21.9
**75-99**	41.9	51.3	8.0	7.9	16.4	18.1	2.2	2.7	21.4	17.6

Seven-year survival

Age group (years)	Ovary		Pancreas		Prostate		Rectum		Stomach	
	**Men**	**Women**	**Men**	**Women**	**Men**	**Women**	**Men**	**Women**	**Men**	**Women**
**15-44**	-	46.5	5.7	8.2	15.5	-	32.7	36.9	12.2	13.3
**45-54**	-	27.0	3.2	3.4	24.9	-	30.1	34.6	10.1	11.3
**55-64**	-	19.2	2.3	2.2	31.6	-	30.2	34.4	8.3	9.7
**65-74**	-	15.0	1.9	1.8	36.1	-	29.4	31.9	6.2	7.5
**75-99**	-	11.6	1.8	1.5	26.6	-	23.4	20.9	3.7	3.9

Seven-year survival

Age group (years)	Testis		Uterus	
	**Men**	**Women**	**Men**	**Women**
**15-44**	82.3	-	-	81.0
**45-54**	82.2	-	-	77.5
**55-64**	75.6	-	-	70.8
**65-74**	62.5	-	-	57.1
**75-99**	40.5	-	-	38.3

Appendix Table 9. Seven-year survival by age and sex, for each cancer: adults diagnosed in England and Wales during 1990-91

Age group (years)	Bladder Men	Bladder Women	Brain Men	Brain Women	Breast Men	Breast Women	Cervix Men	Cervix Women	Colon Men	Colon Women
**15-44**	83.7	72.8	34.1	38.6	-	65.4	-	74.9	44.0	48.6
**45-54**	74.4	67.8	10.9	15.2	-	70.3	-	60.4	38.7	41.3
**55-64**	66.2	62.7	4.0	6.0	-	68.1	-	49.1	39.2	41.2
**65-74**	59.4	56.1	1.7	2.4	-	63.3	-	39.1	38.0	40.3
**75-99**	47.6	37.9	1.1	1.6	-	49.2	-	24.4	32.0	30.2

Age group (years)	HL Men	HL Women	Kidney Men	Kidney Women	Larynx Men	Larynx Women	Leukaemia Men	Leukaemia Women	Lung Men	Lung Women
**15-44**	84.3	86.7	50.9	51.8	70.1	-	39.1	39.8	12.4	12.2
**45-54**	70.0	72.7	42.0	44.4	64.6	-	39.1	38.0	9.5	8.6
**55-64**	53.2	55.4	36.5	37.7	60.1	-	34.4	35.6	6.6	5.9
**65-74**	34.3	38.5	32.6	30.4	56.5	-	24.7	28.0	4.1	4.1
**75-99**	18.2	20.7	23.3	18.7	50.4	-	12.6	16.0	1.6	2.0

Age group (years)	Melanoma Men	Melanoma Women	Myeloma Men	Myeloma Women	NHL Men	NHL Women	Oesophagus Men	Oesophagus Women	Other Men	Other Women
**15-44**	71.5	87.0	36.4	39.6	57.5	61.2	7.8	14.0	49.2	67.3
**45-54**	67.3	82.9	26.8	29.8	50.3	56.5	6.5	11.3	37.0	49.2
**55-64**	64.0	79.7	19.1	21.5	40.8	48.1	5.9	9.4	27.3	36.1
**65-74**	60.7	75.4	13.0	14.4	30.8	35.4	4.7	7.1	20.1	25.8
**75-99**	53.9	62.6	7.5	7.8	20.3	21.6	2.3	2.6	18.0	18.3

Age group (years)	Ovary Men	Ovary Women	Pancreas Men	Pancreas Women	Prostate Men	Prostate Women	Rectum Men	Rectum Women	Stomach Men	Stomach Women
**15-44**	-	58.7	6.4	10.3	43.8	-	39.0	46.0	16.0	18.2
**45-54**	-	36.1	3.7	4.2	49.3	-	37.9	45.0	13.7	16.2
**55-64**	-	24.9	2.4	2.3	51.8	-	37.9	44.4	11.4	13.8
**65-74**	-	18.3	1.7	1.6	50.2	-	35.8	40.5	8.9	11.1
**75-99**	-	12.2	1.3	1.1	33.0	-	27.5	26.2	5.4	5.8

Age group (years)	Testis Men	Testis Women	Uterus Men	Uterus Women
**15-44**	92.4	-	-	81.6
**45-54**	91.3	-	-	80.1
**55-64**	86.6	-	-	74.3
**65-74**	77.0	-	-	62.5
**75-99**	55.0	-	-	42.9

All values: Seven-year survival

Appendix Table 9. Seven-year survival by age and sex, for each cancer: adults diagnosed in England and Wales during 2000-01

Age group (years)	Bladder Men	Bladder Women	Brain Men	Brain Women	Breast Men	Breast Women	Cervix Men	Cervix Women	Colon Men	Colon Women
**15-44**	80.9	66.9	42.4	47.9	-	77.5	-	81.5	53.8	58.3
**45-54**	72.1	63.6	14.8	19.9	-	83.3	-	66.7	49.6	52.2
**55-64**	65.5	59.6	5.7	8.1	-	82.5	-	55.4	50.3	52.5
**65-74**	58.6	52.5	2.3	3.0	-	77.6	-	43.1	48.3	50.9
**75-99**	47.2	34.4	1.3	1.6	-	61.6	-	26.7	41.3	39.6

Age group (years)	HL Men	HL Women	Kidney Men	Kidney Women	Larynx Men	Larynx Women	Leukaemia Men	Leukaemia Women	Lung Men	Lung Women
**15-44**	90.2	91.5	61.8	64.9	72.6	-	55.6	54.1	14.4	15.6
**45-54**	79.1	80.3	52.2	57.3	66.1	-	53.7	51.3	10.0	11.5
**55-64**	64.4	65.0	45.9	49.9	62.5	-	47.9	47.8	7.2	8.5
**65-74**	45.0	48.4	40.5	40.1	59.8	-	35.0	37.4	4.9	5.7
**75-99**	26.3	26.7	28.9	24.9	54.6	-	18.9	21.3	2.2	2.9

Age group (years)	Melanoma Men	Melanoma Women	Myeloma Men	Myeloma Women	NHL Men	NHL Women	Oesophagus Men	Oesophagus Women	Other Men	Other Women
**15-44**	82.4	92.3	50.2	57.1	69.9	74.1	11.4	19.0	57.7	74.6
**45-54**	79.0	89.1	40.7	43.8	62.6	69.5	10.9	15.9	44.8	56.9
**55-64**	76.0	87.0	31.5	32.6	53.9	62.0	10.3	13.4	33.6	43.6
**65-74**	72.6	83.4	20.5	21.1	42.6	48.2	8.1	9.6	22.9	29.7
**75-99**	65.3	73.0	11.1	10.0	29.6	30.7	4.2	3.5	17.7	19.3

Age group (years)	Ovary Men	Ovary Women	Pancreas Men	Pancreas Women	Prostate Men	Prostate Women	Rectum Men	Rectum Women	Stomach Men	Stomach Women
**15-44**	-	72.4	10.0	14.3	77.7	-	51.3	59.1	21.0	24.1
**45-54**	-	49.2	5.3	6.4	79.1	-	51.6	59.1	18.6	21.9
**55-64**	-	35.9	3.3	3.6	78.2	-	51.4	58.3	16.1	19.2
**65-74**	-	25.8	2.2	2.1	73.7	-	48.0	53.0	12.8	15.4
**75-99**	-	16.1	1.4	1.1	54.0	-	37.8	36.6	8.2	8.4

Age group (years)	Testis Men	Testis Women	Uterus Men	Uterus Women
**15-44**	96.3	-	-	83.9
**45-54**	95.0	-	-	83.7
**55-64**	91.9	-	-	79.7
**65-74**	84.1	-	-	69.7
**75-99**	63.6	-	-	51.6

All values: Seven-year survival

Appendix Table 9. Seven-year survival by age and sex, for each cancer: adults diagnosed in England and Wales during 2005-06

Age group (years)	Bladder Men	Bladder Women	Brain Men	Brain Women	Breast Men	Breast Women	Cervix Men	Cervix Women	Colon Men	Colon Women
**15-44**	77.9	62.8	47.3	52.9	-	82.1	-	83.9	58.2	62.3
**45-54**	70.3	60.6	18.1	23.8	-	87.4	-	68.8	54.5	56.6
**55-64**	63.9	57.0	7.3	10.0	-	87.0	-	57.3	55.2	56.9
**65-74**	57.2	50.2	2.9	3.6	-	83.0	-	44.7	52.9	55.0
**75-99**	45.5	31.6	1.5	1.8	-	66.5	-	24.8	45.3	43.4

Age group (years)	HL Men	HL Women	Kidney Men	Kidney Women	Larynx Men	Larynx Women	Leukaemia Men	Leukaemia Women	Lung Men	Lung Women
**15-44**	92.3	93.4	67.5	71.8	71.6	-	63.0	61.0	16.5	18.9
**45-54**	83.0	83.9	58.3	64.9	65.8	-	60.6	58.1	11.3	14.5
**55-64**	69.3	71.1	51.9	57.5	62.3	-	54.5	54.0	8.3	11.0
**65-74**	49.1	53.8	45.9	47.1	60.2	-	41.0	43.0	6.1	7.7
**75-99**	29.1	30.2	32.7	29.5	55.4	-	22.4	24.6	3.0	3.9

Age group (years)	Melanoma Men	Melanoma Women	Myeloma Men	Myeloma Women	NHL Men	NHL Women	Oesophagus Men	Oesophagus Women	Other Men	Other Women
**15-44**	86.5	94.2	59.3	65.5	75.8	79.8	14.3	22.5	62.6	78.1
**45-54**	83.8	91.6	50.6	53.3	69.6	76.1	14.3	19.3	50.4	61.9
**55-64**	81.1	89.7	40.6	40.9	61.1	69.3	13.8	16.6	38.1	47.8
**65-74**	77.8	86.8	27.7	27.3	50.0	56.2	11.1	12.1	25.8	33.1
**75-99**	70.9	77.4	15.0	13.2	36.4	37.3	5.8	4.4	18.7	20.5

Age group (years)	Ovary Men	Ovary Women	Pancreas Men	Pancreas Women	Prostate Men	Prostate Women	Rectum Men	Rectum Women	Stomach Men	Stomach Women
**15-44**	-	77.4	12.4	19.5	88.8	-	56.6	64.1	23.1	26.9
**45-54**	-	55.8	6.7	8.8	89.1	-	57.5	64.4	20.9	24.8
**55-64**	-	41.1	4.2	4.8	88.0	-	57.3	63.5	18.2	21.9
**65-74**	-	29.7	2.6	2.7	84.1	-	53.3	57.8	14.7	17.6
**75-99**	-	17.8	1.6	1.4	66.9	-	42.2	40.5	9.6	9.7

Age group (years)	Testis Men	Testis Women	Uterus Men	Uterus Women
**15-44**	97.1	-	-	84.6
**45-54**	95.9	-	-	85.2
**55-64**	92.8	-	-	81.9
**65-74**	85.4	-	-	72.7
**75-99**	65.0	-	-	55.5

Appendix Table 9. Seven-year survival by age and sex, for each cancer: adults diagnosed in England and Wales during 2010-11

Age group (years)	Bladder		Brain		Breast		Cervix		Colon	
	Men	Women	Men	Women	Men	Women	Men	Women	Men	Women
**15-44**	75.0	58.5	54.3	58.6	-	84.0	-	85.8	61.0	65.2
**45-54**	68.1	57.5	21.8	28.2	-	89.5	-	70.6	57.9	59.4
**55-64**	61.9	54.2	8.8	11.9	-	89.3	-	58.0	58.5	59.8
**65-74**	55.5	47.6	3.8	4.5	-	85.4	-	45.6	56.1	57.9
**75-99**	43.5	29.2	1.8	2.1	-	67.9	-	24.6	47.7	45.3

Seven-year survival

Age group (years)	HL		Kidney		Larynx		Leukaemia		Lung	
	Men	Women	Men	Women	Men	Women	Men	Women	Men	Women
**15-44**	93.7	94.8	72.6	77.8	70.9	-	69.3	67.5	20.0	24.6
**45-54**	85.0	87.3	64.4	71.5	64.2	-	66.6	64.4	13.4	19.2
**55-64**	71.8	75.1	57.7	64.0	60.5	-	59.7	59.6	10.1	15.0
**65-74**	53.1	58.4	51.7	53.9	58.9	-	46.3	48.7	8.0	11.2
**75-99**	31.4	34.8	36.7	34.5	54.5	-	25.8	28.2	4.2	5.7

Seven-year survival

Age group (years)	Melanoma		Myeloma		NHL		Oesophagus		Other	
	Men	Women	Men	Women	Men	Women	Men	Women	Men	Women
**15-44**	90.1	95.7	67.6	75.0	80.5	84.3	16.2	27.3	67.9	81.7
**45-54**	87.8	93.8	60.5	63.9	74.9	81.3	17.0	23.7	56.4	66.8
**55-64**	85.4	92.2	50.3	50.5	66.7	74.8	16.6	20.4	42.9	52.1
**65-74**	82.7	89.9	36.1	36.0	56.6	63.2	13.5	15.1	29.6	37.1
**75-99**	76.4	81.9	20.3	18.1	42.1	43.3	7.0	5.5	20.4	22.5

Seven-year survival

Age group (years)	Ovary		Pancreas		Prostate		Rectum		Stomach	
	Men	Women	Men	Women	Men	Women	Men	Women	Men	Women
**15-44**	-	81.1	14.7	22.4	93.9	-	59.9	66.9	25.1	29.6
**45-54**	-	60.7	8.5	11.4	93.7	-	61.2	67.6	22.9	27.6
**55-64**	-	44.7	5.2	6.3	92.5	-	60.8	66.5	20.1	24.4
**65-74**	-	32.6	3.2	3.6	89.3	-	56.7	60.8	16.5	19.8
**75-99**	-	18.7	1.9	1.7	74.1	-	44.4	42.3	10.7	11.1

Seven-year survival

Age group (years)	Testis		Uterus	
	Men	Women	Men	Women
**15-44**	97.5	-	-	84.6
**45-54**	96.3	-	-	85.9
**55-64**	93.0	-	-	82.5
**65-74**	86.0	-	-	74.1
**75-99**	63.4	-	-	57.7

Appendix Table 9. Seven-year survival by age and sex, for each cancer: adults diagnosed in England and Wales during 2015-16

Age group	Seven-year survival									
	Bladder		Brain		Breast		Cervix		Colon	
(years)	Men	Women	Men	Women	Men	Women	Men	Women	Men	Women
**15-44**	81.2	57.4	64.3	70.6	-	88.4	-	88.5	65.2	69.0
**45-54**	64.8	57.4	30.2	39.0	-	93.0	-	74.8	62.9	63.6
**55-64**	51.2	54.8	13.5	18.1	-	92.7	-	63.0	63.4	63.8
**65-74**	41.6	48.6	5.7	6.7	-	89.5	-	50.2	60.5	61.1
**75-99**	28.0	29.5	2.2	2.5	-	73.6	-	28.4	49.6	46.2

Age group	Seven-year survival									
	HL		Kidney		Larynx		Leukaemia		Lung	
(years)	Men	Women	Men	Women	Men	Women	Men	Women	Men	Women
**15-44**	71.2	54.0	58.1	65.0	0.0	-	0.0	87.4	62.6	66.7
**45-54**	65.5	54.2	25.3	33.4	0.0	-	0.0	71.9	59.9	61.2
**55-64**	59.9	51.6	11.1	15.2	0.0	-	0.0	59.0	60.5	61.7
**65-74**	53.5	45.4	4.8	5.8	0.0	-	0.0	45.8	57.8	59.5
**75-99**	41.5	26.6	2.2	2.5	0.0	-	0.0	24.9	48.4	46.1

Age group	Seven-year survival									
	Melanoma		Myeloma		NHL		Oesophagus		Other	
(years)	Men	Women	Men	Women	Men	Women	Men	Women	Men	Women
**15-44**	93.1	97.1	74.5	82.3	84.0	87.6	17.0	31.5	73.3	85.3
**45-54**	91.5	95.6	69.0	73.2	78.6	84.7	18.7	28.6	62.1	71.5
**55-64**	89.7	94.4	60.3	62.0	71.3	79.3	18.5	24.8	49.0	57.7
**65-74**	87.3	92.7	45.2	46.8	61.4	68.1	14.9	18.8	34.1	41.9
**75-99**	81.8	86.4	26.5	25.3	46.7	48.5	7.7	7.2	22.8	25.5

Age group	Seven-year survival									
	Ovary		Pancreas		Prostate		Rectum		Stomach	
(years)	Men	Women	Men	Women	Men	Women	Men	Women	Men	Women
**15-44**	-	84.7	18.3	28.8	95.3	-	61.5	68.5	27.0	32.4
**45-54**	-	64.0	10.7	14.9	95.0	-	63.5	69.7	24.8	30.1
**55-64**	-	48.7	6.7	8.5	93.7	-	63.1	68.6	22.1	27.1
**65-74**	-	34.7	4.1	4.8	90.2	-	58.4	62.3	18.2	22.2
**75-99**	-	19.2	2.2	2.1	73.4	-	45.1	42.5	11.8	12.5

Age group	Seven-year survival			
	Testis		Uterus	
(years)	Men	Women	Men	Women
**15-44**	97.7	-	-	83.8
**45-54**	96.2	-	-	85.7
**55-64**	93.2	-	-	82.9
**65-74**	85.1	-	-	74.4
**75-99**	58.8	-	-	57.9

Appendix Table 9. Seven-year survival by age and sex, for each cancer: adults diagnosed in England and Wales during 2018

Age group (years)	Bladder Men	Bladder Women	Brain Men	Brain Women	Breast Men	Breast Women	Cervix Men	Cervix Women	Colon Men	Colon Women
**15-44**	69.6	51.8	60.6	67.4	-	83.1	-	87.7	62.1	65.6
**45-54**	64.1	52.5	26.8	35.4	-	90.6	-	72.2	60.6	61.9
**55-64**	58.9	50.2	12.3	17.0	-	90.7	-	59.9	61.3	62.5
**65-74**	52.2	43.5	5.2	6.6	-	86.0	-	45.1	58.3	60.1
**75-99**	40.4	26.1	2.4	2.7	-	66.3	-	23.1	48.7	46.9

Age group (years)	HL Men	HL Women	Kidney Men	Kidney Women	Larynx Men	Larynx Women	Leukaemia Men	Leukaemia Women	Lung Men	Lung Women
**15-44**	95.2	96.6	79.7	84.5	67.3	-	76.7	75.9	26.7	35.4
**45-54**	87.2	89.8	72.5	79.2	59.8	-	73.5	72.7	17.8	29.6
**55-64**	74.5	80.5	66.5	72.6	56.1	-	67.2	68.0	14.0	24.6
**65-74**	55.3	65.4	60.3	62.6	55.1	-	52.8	56.6	12.2	19.4
**75-99**	30.7	40.5	44.4	41.9	51.5	-	30.6	34.4	7.5	11.7

Age group (years)	Melanoma Men	Melanoma Women	Myeloma Men	Myeloma Women	NHL Men	NHL Women	Oesophagus Men	Oesophagus Women	Other Men	Other Women
**15-44**	94.4	97.6	78.4	85.8	85.4	88.9	16.9	35.0	75.6	86.5
**45-54**	92.9	96.3	72.9	77.2	80.1	86.2	19.2	31.2	64.7	73.7
**55-64**	91.4	95.3	64.5	66.5	73.4	81.0	19.2	27.2	51.9	60.5
**65-74**	89.2	93.7	49.0	51.4	63.2	69.7	15.4	20.5	36.1	43.9
**75-99**	84.2	88.5	29.8	30.6	48.9	50.7	8.0	8.3	24.0	27.1

Age group (years)	Ovary Men	Ovary Women	Pancreas Men	Pancreas Women	Prostate Men	Prostate Women	Rectum Men	Rectum Women	Stomach Men	Stomach Women
**15-44**	-	85.7	20.2	31.9	95.6	-	62.0	69.1	28.1	33.8
**45-54**	-	65.6	12.0	16.8	95.2	-	64.5	70.5	25.7	31.6
**55-64**	-	50.1	7.6	9.8	93.8	-	64.0	69.3	23.0	28.4
**65-74**	-	35.3	4.5	5.3	89.7	-	58.9	62.7	19.1	23.3
**75-99**	-	19.6	2.4	2.4	72.2	-	45.0	42.9	12.5	13.4

Age group (years)	Testis Men	Testis Women	Uterus Men	Uterus Women
**15-44**	97.8	-	-	82.7
**45-54**	96.2	-	-	85.5
**55-64**	93.0	-	-	82.8
**65-74**	84.1	-	-	74.0
**75-99**	61.5	-	-	57.7

All values: Seven-year survival

Appendix Table 10. Ten-year survival by age and sex, for each cancer: adults diagnosed in England and Wales during 1971-72

Age group (years)	Bladder Men	Bladder Women	Brain Men	Brain Women	Breast Men	Breast Women	Cervix Men	Cervix Women	Colon Men	Colon Women
**15-44**	79.9	65.1	19.6	21.5	-	47.9	-	62.3	30.2	35.9
**45-54**	62.2	55.9	6.6	7.0	-	45.3	-	50.1	22.6	23.8
**55-64**	47.8	47.1	3.3	3.4	-	40.1	-	41.6	23.3	23.4
**65-74**	38.2	37.8	2.1	2.0	-	40.2	-	33.8	23.8	24.0
**75-99**	25.5	20.4	2.3	2.3	-	38.6	-	22.7	21.6	19.0

Ten-year survival

Age group (years)	HL Men	HL Women	Kidney Men	Kidney Women	Larynx Men	Larynx Women	Leukaemia Men	Leukaemia Women	Lung Men	Lung Women
**15-44**	59.9	66.2	33.1	32.8	69.0	-	11.6	13.9	10.4	9.2
**45-54**	43.1	46.8	26.8	27.2	63.5	-	13.4	13.3	9.5	6.0
**55-64**	27.8	29.5	23.8	24.0	58.1	-	12.2	13.4	6.5	4.1
**65-74**	15.6	17.4	22.7	21.0	52.5	-	8.2	11.2	3.3	2.8
**75-99**	8.6	7.8	17.8	15.2	44.4	-	4.0	8.0	1.0	1.4

Ten-year survival

Age group (years)	Melanoma Men	Melanoma Women	Myeloma Men	Myeloma Women	NHL Men	NHL Women	Oesophagus Men	Oesophagus Women	Other Men	Other Women
**15-44**	38.9	64.9	24.0	15.0	38.8	41.8	9.8	10.1	38.5	48.7
**45-54**	35.6	60.1	12.2	10.4	31.9	35.2	6.3	8.1	28.5	31.5
**55-64**	34.2	56.9	9.2	8.6	24.2	28.2	5.2	7.2	23.6	23.1
**65-74**	33.1	51.8	8.9	7.9	17.4	20.2	4.5	6.2	21.9	18.0
**75-99**	30.2	39.1	10.0	7.8	11.7	13.6	2.5	3.1	26.6	16.8

Ten-year survival

Age group (years)	Ovary Men	Ovary Women	Pancreas Men	Pancreas Women	Prostate Men	Prostate Women	Rectum Men	Rectum Women	Stomach Men	Stomach Women
**15-44**	-	35.0	5.1	6.6	2.0	-	28.3	29.6	9.4	9.3
**45-54**	-	20.4	3.1	3.2	7.1	-	24.7	27.3	7.6	7.8
**55-64**	-	15.5	2.3	2.2	15.1	-	25.4	27.8	6.0	6.4
**65-74**	-	13.9	2.1	2.1	24.4	-	26.3	27.3	4.5	5.1
**75-99**	-	13.8	2.4	2.4	22.3	-	22.9	19.5	2.6	2.7

Ten-year survival

Age group (years)	Testis Men	Testis Women	Uterus Men	Uterus Women
**15-44**	62.9	-	-	79.6
**45-54**	66.7	-	-	74.9
**55-64**	57.8	-	-	66.3
**65-74**	43.9	-	-	52.0
**75-99**	24.8	-	-	34.1

Appendix Table 10. Ten-year survival by age and sex, for each cancer: adults diagnosed in England and Wales during 1980-81

Age group	Ten-year survival									
	Bladder		Brain		Breast		Cervix		Colon	
(years)	Men	Women	Men	Women	Men	Women	Men	Women	Men	Women
**15-44**	82.4	69.9	22.9	26.0	-	51.0	-	67.4	35.0	39.7
**45-54**	67.7	62.2	7.0	8.9	-	52.7	-	51.1	28.2	30.0
**55-64**	56.9	56.2	3.3	4.0	-	49.1	-	41.1	28.9	29.9
**65-74**	48.1	47.7	1.8	2.1	-	46.4	-	32.0	28.8	30.1
**75-99**	36.7	29.8	1.7	2.0	-	38.6	-	19.8	25.7	23.6

Age group	Ten-year survival									
	HL		Kidney		Larynx		Leukaemia		Lung	
(years)	Men	Women	Men	Women	Men	Women	Men	Women	Men	Women
**15-44**	71.1	75.7	40.0	39.6	67.8	-	21.0	23.2	10.3	9.7
**45-54**	53.2	57.2	31.2	32.4	60.8	-	21.9	21.6	8.4	6.2
**55-64**	35.5	40.4	27.1	27.7	55.7	-	19.2	20.3	5.9	4.4
**65-74**	19.6	23.6	24.7	22.5	50.6	-	12.2	15.5	3.0	2.9
**75-99**	10.5	11.2	18.6	15.0	43.1	-	5.8	9.7	1.0	1.4

Age group	Ten-year survival									
	Melanoma		Myeloma		NHL		Oesophagus		Other	
(years)	Men	Women	Men	Women	Men	Women	Men	Women	Men	Women
**15-44**	53.7	76.1	24.8	22.5	45.0	48.2	7.6	10.6	41.8	58.1
**45-54**	49.7	71.0	14.0	14.2	36.4	41.3	5.3	8.4	29.8	38.0
**55-64**	47.4	67.9	9.8	10.5	28.1	33.5	4.6	7.3	23.3	27.8
**65-74**	45.3	62.8	7.6	7.7	19.5	22.8	3.8	5.8	19.2	20.2
**75-99**	41.2	50.2	6.6	5.9	12.8	14.0	2.1	2.5	20.8	16.8

Age group	Ten-year survival									
	Ovary		Pancreas		Prostate		Rectum		Stomach	
(years)	Men	Women	Men	Women	Men	Women	Men	Women	Men	Women
**15-44**	-	43.7	5.1	7.4	9.2	-	29.6	33.6	11.0	12.1
**45-54**	-	24.5	2.8	2.9	17.7	-	27.4	31.8	9.1	10.2
**55-64**	-	17.5	1.9	1.8	24.7	-	27.8	31.9	7.4	8.6
**65-74**	-	14.0	1.5	1.4	30.0	-	27.5	30.0	5.6	6.7
**75-99**	-	12.0	1.5	1.3	22.0	-	22.7	20.2	3.4	3.5

Age group	Ten-year survival			
	Testis		Uterus	
(years)	Men	Women	Men	Women
**15-44**	82.0	-	-	79.1
**45-54**	81.7	-	-	75.7
**55-64**	74.9	-	-	68.7
**65-74**	61.4	-	-	54.7
**75-99**	38.9	-	-	36.6

Appendix Table 10. Ten-year survival by age and sex, for each cancer: adults diagnosed in England and Wales during 1990-91

Age group (years)	Bladder		Brain		Breast		Cervix		Colon	
	Men	Women	Men	Women	Men	Women	Men	Women	Men	Women
**15-44**	81.7	70.3	28.6	32.9	-	59.4	-	73.3	41.5	46.2
**45-54**	71.0	65.0	8.9	12.2	-	65.0	-	57.1	36.3	39.0
**55-64**	61.8	59.6	3.6	5.2	-	62.8	-	44.7	37.2	39.2
**65-74**	54.6	52.8	1.7	2.3	-	57.8	-	34.5	36.3	38.8
**75-99**	43.1	34.5	1.4	1.8	-	43.4	-	20.7	31.7	30.3

Age group (years)	HL		Kidney		Larynx		Leukaemia		Lung	
	Men	Women	Men	Women	Men	Women	Men	Women	Men	Women
**15-44**	81.7	84.5	47.5	48.8	67.0	-	35.3	36.6	10.3	10.8
**45-54**	65.3	68.9	38.0	41.0	60.4	-	34.3	33.4	7.7	7.4
**55-64**	47.5	50.5	32.5	34.0	55.1	-	29.3	30.1	5.1	4.9
**65-74**	28.6	33.4	28.8	26.8	51.2	-	19.6	22.6	3.0	3.3
**75-99**	14.2	16.7	20.0	15.8	45.1	-	8.7	12.2	1.1	1.5

Age group (years)	Melanoma		Myeloma		NHL		Oesophagus		Other	
	Men	Women	Men	Women	Men	Women	Men	Women	Men	Women
**15-44**	68.4	84.9	28.8	32.6	54.1	57.8	6.9	12.5	46.4	64.8
**45-54**	64.4	80.6	19.3	22.2	45.2	51.5	5.8	10.0	33.9	46.0
**55-64**	61.5	77.7	12.6	14.4	35.1	42.1	5.2	8.4	24.7	33.0
**65-74**	58.7	73.6	8.2	8.8	25.2	29.2	4.2	6.3	18.2	23.2
**75-99**	53.3	61.4	5.2	4.9	16.1	17.0	2.1	2.4	16.8	16.9

Age group (years)	Ovary		Pancreas		Prostate		Rectum		Stomach	
	Men	Women	Men	Women	Men	Women	Men	Women	Men	Women
**15-44**	-	56.1	5.7	9.2	34.3	-	35.6	42.4	14.4	16.6
**45-54**	-	32.9	3.1	3.5	41.0	-	34.6	41.7	12.3	14.6
**55-64**	-	22.3	1.9	1.8	44.5	-	34.8	41.3	10.2	12.4
**65-74**	-	16.6	1.3	1.2	43.7	-	33.2	37.9	8.1	9.9
**75-99**	-	12.0	1.1	0.8	27.6	-	26.2	24.9	4.9	5.2

Age group (years)	Testis		Uterus	
	Men	Women	Men	Women
**15-44**	92.2	-	-	79.5
**45-54**	90.9	-	-	78.1
**55-64**	85.8	-	-	72.1
**65-74**	75.7	-	-	59.9
**75-99**	52.6	-	-	40.9

Appendix Table 10. Ten-year survival by age and sex, for each cancer: adults diagnosed in England and Wales during 2000-01

Age group (years)	Bladder		Brain		Breast		Cervix		Colon	
	Men	Women	Men	Women	Men	Women	Men	Women	Men	Women
**15-44**	79.0	64.0	36.3	41.8	-	72.9	-	80.4	51.5	56.0
**45-54**	68.9	60.6	12.3	16.4	-	79.9	-	64.0	47.2	49.9
**55-64**	61.6	56.5	5.1	7.0	-	79.1	-	51.6	48.0	50.4
**65-74**	54.5	49.2	2.3	2.9	-	73.3	-	38.9	46.3	49.4
**75-99**	43.5	31.0	1.7	1.9	-	55.3	-	23.1	40.8	39.6

Age group (years)	HL		Kidney		Larynx		Leukaemia		Lung	
	Men	Women	Men	Women	Men	Women	Men	Women	Men	Women
**15-44**	88.8	90.2	58.9	62.5	68.9	-	52.2	52.0	11.8	13.9
**45-54**	75.8	77.8	48.3	54.1	61.0	-	49.4	47.9	7.9	9.9
**55-64**	59.5	61.4	41.6	46.2	56.6	-	43.0	43.3	5.5	7.0
**65-74**	38.9	44.2	36.3	36.1	53.6	-	29.7	32.2	3.6	4.5
**75-99**	20.9	22.9	24.9	21.2	48.3	-	14.3	17.0	1.5	2.2

Age group (years)	Melanoma		Myeloma		NHL		Oesophagus		Other	
	Men	Women	Men	Women	Men	Women	Men	Women	Men	Women
**15-44**	80.3	91.1	42.5	50.6	67.3	71.6	10.1	17.1	55.1	72.5
**45-54**	76.9	87.7	31.6	35.2	58.3	65.5	9.8	14.2	41.6	53.7
**55-64**	74.2	85.6	22.4	23.7	48.7	56.9	9.4	12.0	30.6	40.2
**65-74**	71.2	82.2	13.2	13.4	37.0	42.2	7.3	8.7	20.6	26.8
**75-99**	65.3	72.2	7.0	6.0	25.1	25.7	3.8	3.1	16.4	17.6

Age group (years)	Ovary		Pancreas		Prostate		Rectum		Stomach	
	Men	Women	Men	Women	Men	Women	Men	Women	Men	Women
**15-44**	-	70.1	9.0	12.9	71.9	-	48.2	56.1	18.9	22.2
**45-54**	-	45.3	4.5	5.4	74.2	-	48.4	56.2	16.7	20.0
**55-64**	-	32.3	2.6	2.8	73.6	-	48.3	55.5	14.4	17.5
**65-74**	-	23.0	1.6	1.6	68.9	-	45.2	50.6	11.5	14.0
**75-99**	-	15.2	1.1	0.9	48.3	-	36.2	35.4	7.4	7.6

Age group (years)	Testis		Uterus	
	Men	Women	Men	Women
**15-44**	96.1	-	-	82.1
**45-54**	94.6	-	-	82.1
**55-64**	91.2	-	-	77.9
**65-74**	82.7	-	-	67.5
**75-99**	60.5	-	-	49.7

All tables: Ten-year survival

Appendix Table 10. Ten-year survival by age and sex, for each cancer: adults diagnosed in England and Wales during 2005-06

Age group (years)	Bladder		Brain		Breast		Cervix		Colon	
	Men	Women	Men	Women	Men	Women	Men	Women	Men	Women
**15-44**	75.9	59.7	41.0	46.6	-	77.9	-	82.8	56.2	60.2
**45-54**	67.2	57.5	15.1	19.7	-	84.5	-	66.0	52.2	54.5
**55-64**	60.2	53.8	6.4	8.7	-	84.1	-	53.5	53.0	55.0
**65-74**	53.3	46.9	2.8	3.5	-	79.0	-	40.3	50.9	53.7
**75-99**	42.2	28.4	1.9	2.1	-	59.6	-	21.3	44.8	43.5

Ten-year survival

Age group (years)	HL		Kidney		Larynx		Leukaemia		Lung	
	Men	Women	Men	Women	Men	Women	Men	Women	Men	Women
**15-44**	91.1	92.3	64.9	69.6	67.0	-	60.0	59.5	13.5	16.9
**45-54**	80.1	81.6	54.6	62.1	59.9	-	56.8	55.5	8.8	12.5
**55-64**	64.3	67.6	47.7	54.0	55.5	-	50.0	50.3	6.3	9.2
**65-74**	42.2	49.4	41.5	43.0	53.2	-	35.7	38.3	4.5	6.2
**75-99**	22.5	25.9	28.3	25.4	48.3	-	17.5	20.2	2.0	2.9

Ten-year survival

Age group (years)	Melanoma		Myeloma		NHL		Oesophagus		Other	
	Men	Women	Men	Women	Men	Women	Men	Women	Men	Women
**15-44**	85.0	93.3	52.4	59.5	73.7	77.8	12.8	20.6	60.3	76.3
**45-54**	82.2	90.6	41.4	45.0	66.1	72.9	12.9	17.6	47.4	58.9
**55-64**	79.7	88.8	30.5	31.2	56.5	64.9	12.6	15.1	35.0	44.4
**65-74**	76.8	86.0	18.7	18.2	44.8	50.8	10.1	11.0	23.3	30.0
**75-99**	71.2	77.0	9.6	8.0	31.9	32.3	5.2	4.0	17.3	18.7

Ten-year survival

Age group (years)	Ovary		Pancreas		Prostate		Rectum		Stomach	
	Men	Women	Men	Women	Men	Women	Men	Women	Men	Women
**15-44**	-	75.1	11.2	17.9	85.5	-	53.9	61.4	20.7	25.0
**45-54**	-	51.5	5.6	7.6	86.2	-	54.5	61.8	18.6	22.8
**55-64**	-	36.6	3.3	3.8	85.1	-	54.3	61.0	16.2	20.0
**65-74**	-	26.0	2.0	2.1	80.8	-	50.6	55.6	13.1	16.0
**75-99**	-	16.3	1.3	1.1	62.0	-	40.5	39.4	8.5	8.8

Ten-year survival

Age group (years)	Testis		Uterus	
	Men	Women	Men	Women
**15-44**	96.9	-	-	82.8
**45-54**	95.6	-	-	83.7
**55-64**	92.2	-	-	80.2
**65-74**	83.9	-	-	70.6
**75-99**	61.6	-	-	53.6

Appendix Table 10. Ten-year survival by age and sex, for each cancer: adults diagnosed in England and Wales during 2010-11

Age group (years)	Bladder Men	Bladder Women	Brain Men	Brain Women	Breast Men	Breast Women	Cervix Men	Cervix Women	Colon Men	Colon Women
**15-44**	72.9	55.2	47.8	52.1	-	79.3	-	84.7	59.3	63.4
**45-54**	64.9	54.2	18.2	23.5	-	86.5	-	67.6	55.6	57.7
**55-64**	58.1	51.0	7.6	10.3	-	86.2	-	53.8	56.4	58.3
**65-74**	51.6	44.3	3.5	4.3	-	81.1	-	40.8	54.1	56.8
**75-99**	40.2	26.0	2.2	2.4	-	59.2	-	20.7	47.3	45.9

Age group (years)	HL Men	HL Women	Kidney Men	Kidney Women	Larynx Men	Larynx Women	Leukaemia Men	Leukaemia Women	Lung Men	Lung Women
**15-44**	92.6	93.7	70.2	75.9	65.5	-	66.7	66.7	16.4	22.3
**45-54**	81.7	85.0	60.8	68.8	57.2	-	63.2	62.5	10.5	16.8
**55-64**	65.9	71.1	53.4	60.5	52.6	-	55.5	56.6	7.6	12.7
**65-74**	44.6	53.0	47.1	49.6	50.7	-	41.3	44.5	5.9	9.1
**75-99**	22.8	29.1	31.8	29.8	46.3	-	20.8	23.7	2.9	4.3

Age group (years)	Melanoma Men	Melanoma Women	Myeloma Men	Myeloma Women	NHL Men	NHL Women	Oesophagus Men	Oesophagus Women	Other Men	Other Women
**15-44**	89.0	95.2	61.5	70.2	78.7	82.8	14.4	25.3	65.8	80.2
**45-54**	86.7	93.2	51.7	56.3	71.8	78.7	15.3	21.9	53.4	64.0
**55-64**	84.5	91.6	39.7	40.4	62.5	71.2	15.0	18.8	39.7	48.8
**65-74**	82.0	89.5	25.6	25.4	51.7	58.4	12.2	14.0	26.9	33.9
**75-99**	76.9	82.0	13.3	11.4	37.7	38.5	6.2	5.0	18.8	20.7

Age group (years)	Ovary Men	Ovary Women	Pancreas Men	Pancreas Women	Prostate Men	Prostate Women	Rectum Men	Rectum Women	Stomach Men	Stomach Women
**15-44**	-	78.6	13.0	20.5	92.0	-	57.3	64.5	22.4	27.6
**45-54**	-	55.5	7.1	9.9	91.9	-	58.4	65.2	20.3	25.4
**55-64**	-	38.9	4.0	5.0	90.6	-	57.9	64.2	17.7	22.4
**65-74**	-	27.4	2.4	2.7	86.9	-	53.9	58.7	14.5	18.0
**75-99**	-	16.0	1.4	1.3	69.9	-	42.6	41.2	9.4	10.0

Age group (years)	Testis Men	Testis Women	Uterus Men	Uterus Women
**15-44**	97.4	-	-	82.6
**45-54**	96.0	-	-	84.3
**55-64**	92.3	-	-	80.8
**65-74**	84.3	-	-	71.9
**75-99**	59.5	-	-	55.7

All values: Ten-year survival.

Appendix Table 10. Ten-year survival by age and sex, for each cancer: adults diagnosed in England and Wales during 2015-16

Age group	Ten-year survival									
	Bladder		Brain		Breast		Cervix		Colon	
(years)	Men	Women	Men	Women	Men	Women	Men	Women	Men	Women
15-44	68.8	50.6	51.2	58.4	-	77.7	-	86.2	61.1	65.3
45-54	62.1	50.8	21.0	27.8	-	86.8	-	68.7	57.7	59.8
55-64	56.0	48.3	9.3	12.9	-	86.7	-	54.3	58.4	60.6
65-74	49.5	42.0	4.2	5.2	-	80.9	-	40.4	55.8	58.9
75-99	38.2	23.6	2.5	2.7	-	55.4	-	20.5	48.1	47.0

Age group	Ten-year survival									
	HL		Kidney		Larynx		Leukaemia		Lung	
(years)	Men	Women	Men	Women	Men	Women	Men	Women	Men	Women
15-44	93.4	95.1	75.1	80.5	63.2	-	72.2	73.0	20.1	29.0
45-54	82.4	85.9	66.3	74.3	52.7	-	68.4	68.9	12.7	22.9
55-64	66.1	73.4	59.1	66.7	48.4	-	61.1	62.9	9.4	18.3
65-74	44.0	56.0	52.5	55.7	46.9	-	46.3	50.5	7.9	13.7
75-99	20.6	31.9	35.8	34.0	43.0	-	23.9	27.9	4.3	7.2

Age group	Ten-year survival									
	Melanoma		Myeloma		NHL		Oesophagus		Other	
(years)	Men	Women	Men	Women	Men	Women	Men	Women	Men	Women
15-44	92.4	96.8	69.0	78.4	82.4	86.4	14.7	29.5	71.4	84.1
45-54	90.6	95.3	60.8	66.5	75.7	82.7	16.4	26.7	59.1	69.0
55-64	88.9	94.3	49.8	52.4	67.3	76.3	16.3	23.2	45.6	54.6
65-74	86.8	92.7	33.5	35.0	56.5	63.8	13.1	17.6	31.1	38.8
75-99	82.4	86.9	17.7	16.7	42.0	44.0	6.6	6.7	20.9	23.6

Age group	Ten-year survival									
	Ovary		Pancreas		Prostate		Rectum		Stomach	
(years)	Men	Women	Men	Women	Men	Women	Men	Women	Men	Women
15-44	-	82.1	16.0	26.5	93.8	-	58.9	66.1	23.8	30.2
45-54	-	57.6	8.6	12.8	93.5	-	60.7	67.3	21.7	27.9
55-64	-	41.2	5.0	6.7	92.1	-	60.2	66.2	19.3	24.9
65-74	-	27.7	2.9	3.6	87.9	-	55.5	60.2	15.8	20.3
75-99	-	15.2	1.6	1.6	68.9	-	43.1	41.4	10.2	11.3

Age group	Ten-year survival			
	Testis		Uterus	
(years)	Men	Women	Men	Women
15-44	97.6	-	-	81.5
45-54	95.8	-	-	83.9
55-64	92.4	-	-	80.9
65-74	83.2	-	-	71.8
75-99	54.2	-	-	55.3

Appendix Table 10. Ten-year survival by age and sex, for each cancer: adults diagnosed in England and Wales during 2018

Age group (years)	Bladder		Brain		Breast		Cervix		Colon	
	Men	Women	Men	Women	Men	Women	Men	Women	Men	Women
**15-44**	67.1	48.3	53.4	60.7	-	76.2	-	86.4	60.5	64.3
**45-54**	60.5	49.1	22.1	29.6	-	86.7	-	68.7	58.5	60.8
**55-64**	54.8	46.9	10.2	14.3	-	86.7	-	55.1	59.2	61.5
**65-74**	48.1	40.2	4.4	5.9	-	79.8	-	39.4	56.3	59.7
**75-99**	37.1	23.1	2.6	2.9	-	53.5	-	18.7	48.5	48.0

Ten-year survival

Age group (years)	HL		Kidney		Larynx		Leukaemia		Lung	
	Men	Women	Men	Women	Men	Women	Men	Women	Men	Women
**15-44**	93.9	95.6	77.0	82.6	59.8	-	74.7	75.8	22.2	32.7
**45-54**	83.0	87.0	68.6	76.4	50.5	-	70.7	71.8	13.9	26.6
**55-64**	66.3	75.6	61.7	69.0	46.0	-	63.7	66.1	10.5	21.5
**65-74**	43.0	58.0	54.8	57.7	44.7	-	48.1	53.2	9.0	16.3
**75-99**	18.4	32.0	38.0	35.7	41.2	-	25.5	29.9	5.2	9.2

Ten-year survival

Age group (years)	Melanoma		Myeloma		NHL		Oesophagus		Other	
	Men	Women	Men	Women	Men	Women	Men	Women	Men	Women
**15-44**	93.8	97.5	73.8	82.5	83.9	87.9	14.3	33.0	73.8	85.3
**45-54**	92.2	96.2	65.2	71.0	77.3	84.4	16.7	29.4	61.7	71.4
**55-64**	90.8	95.3	54.3	57.1	69.5	78.3	16.7	25.6	48.5	57.5
**65-74**	88.8	93.9	36.9	39.3	58.3	65.7	13.2	19.3	32.9	40.8
**75-99**	84.7	89.2	20.2	20.6	44.1	46.4	6.6	7.8	22.0	25.1

Ten-year survival

Age group (years)	Ovary		Pancreas		Prostate		Rectum		Stomach	
	Men	Women	Men	Women	Men	Women	Men	Women	Men	Women
**15-44**	-	82.9	17.6	29.3	94.2	-	59.5	66.8	24.6	31.6
**45-54**	-	58.7	9.6	14.4	93.7	-	61.6	68.1	22.4	29.2
**55-64**	-	41.9	5.6	7.8	92.2	-	61.0	66.9	20.0	26.2
**65-74**	-	27.5	3.1	3.9	87.2	-	55.9	60.6	16.5	21.3
**75-99**	-	14.8	1.7	1.8	67.5	-	42.8	41.8	10.7	12.1

Ten-year survival

Age group (years)	Testis		Uterus	
	Men	Women	Men	Women
**15-44**	97.6	-	-	80.1
**45-54**	95.8	-	-	83.6
**55-64**	92.2	-	-	80.7
**65-74**	81.9	-	-	71.2
**75-99**	56.9	-	-	54.8

Appendix Figure 1. Trends in age-standardised one-year net survival (%) for all cancers combined (Cancer Survival Index), and for 22 cancers, for men (blue line), women (orange line) and both sexes combined (green line), in England and Wales, for selected periods during 1971-2018

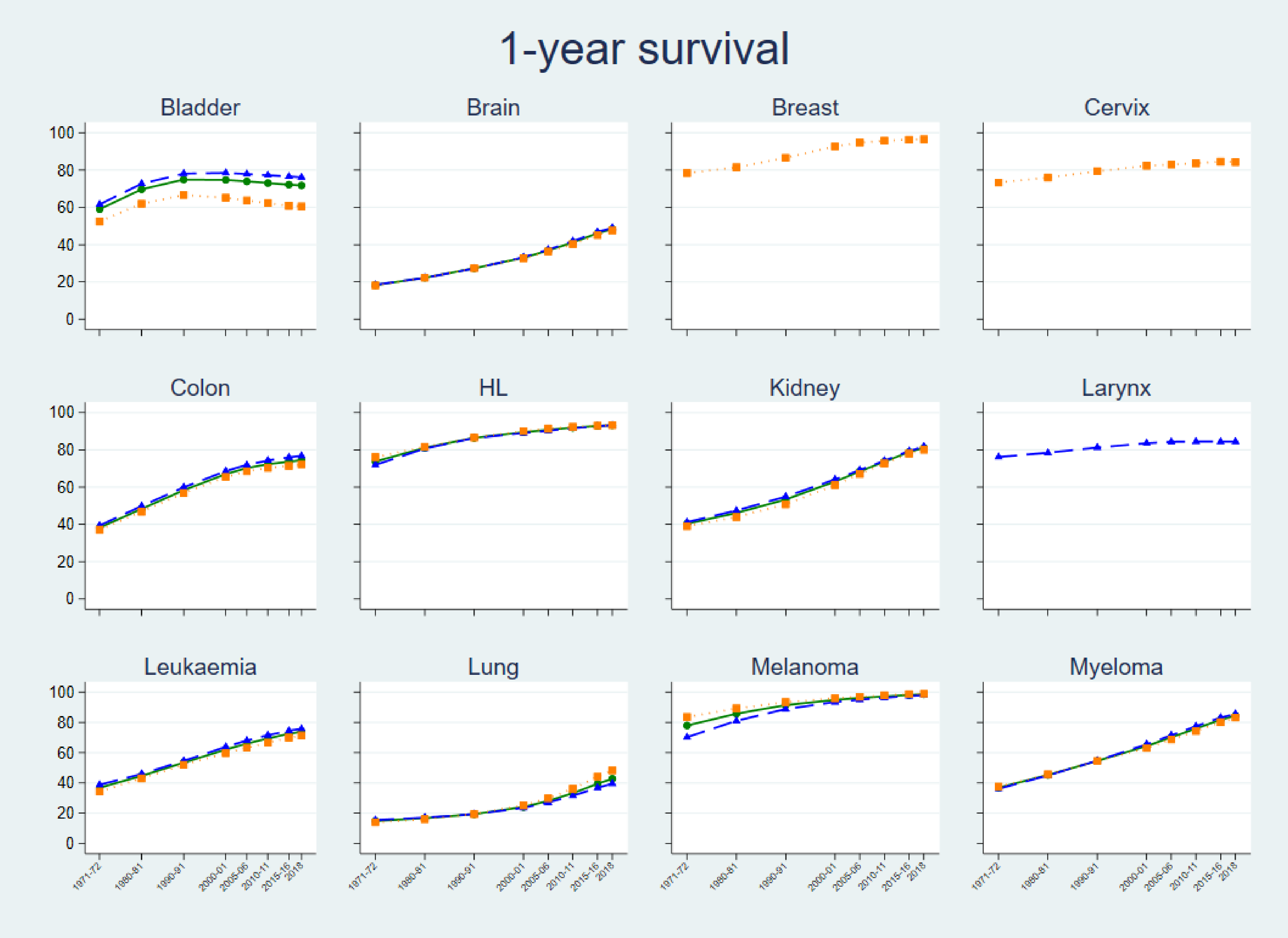

Appendix Figure 1. Trends in age-standardised one-year net survival (%) for all cancers combined (Cancer Survival Index), and for 22 cancers, for men (blue line), women (orange line) and both sexes combined (green line), in England and Wales, for selected periods during 1971-2018

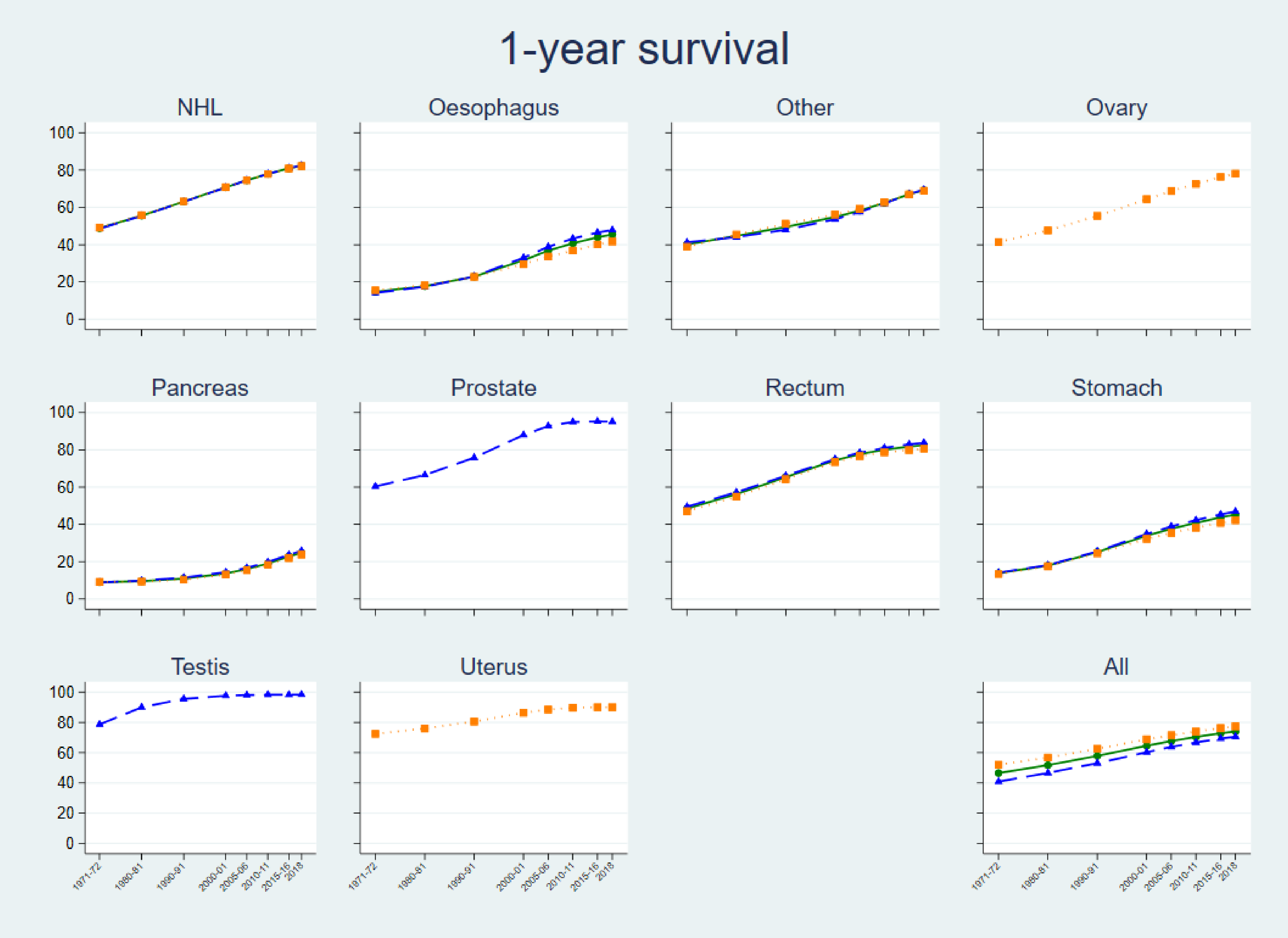

Appendix Figure 2. Trends in age-standardised five-year net survival (%) for all cancers combined (Cancer Survival Index), and for 22 cancers, for men (blue line), women (orange line) and both sexes combined (green line), in England and Wales, for selected periods during 1971-2018

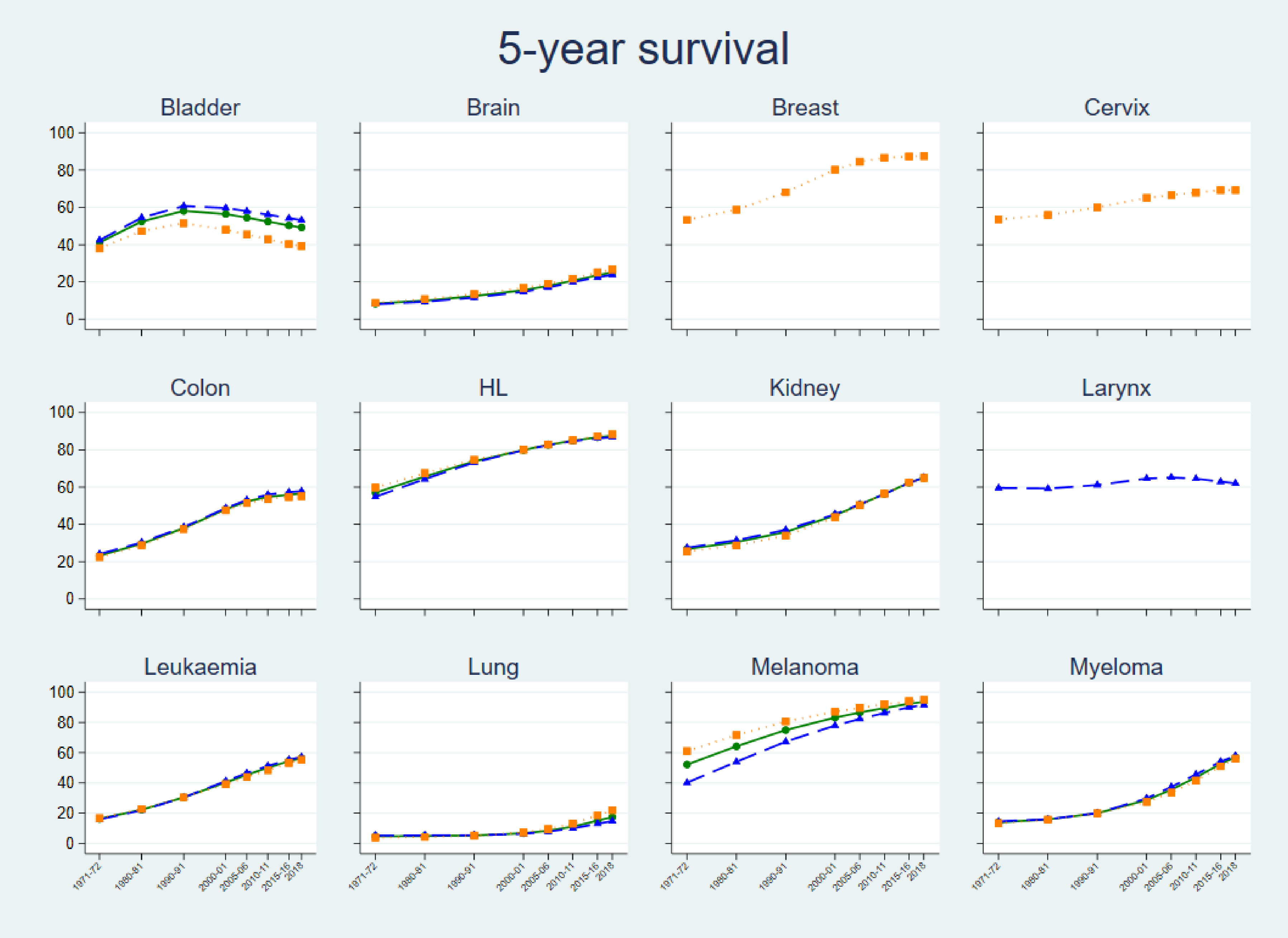

Appendix Figure 2. Trends in age-standardised five-year net survival (%) for all cancers combined (Cancer Survival Index), and for 22 cancers, for men (blue line), women (orange line) and both sexes combined (green line), in England and Wales, for selected periods during 1971-2018

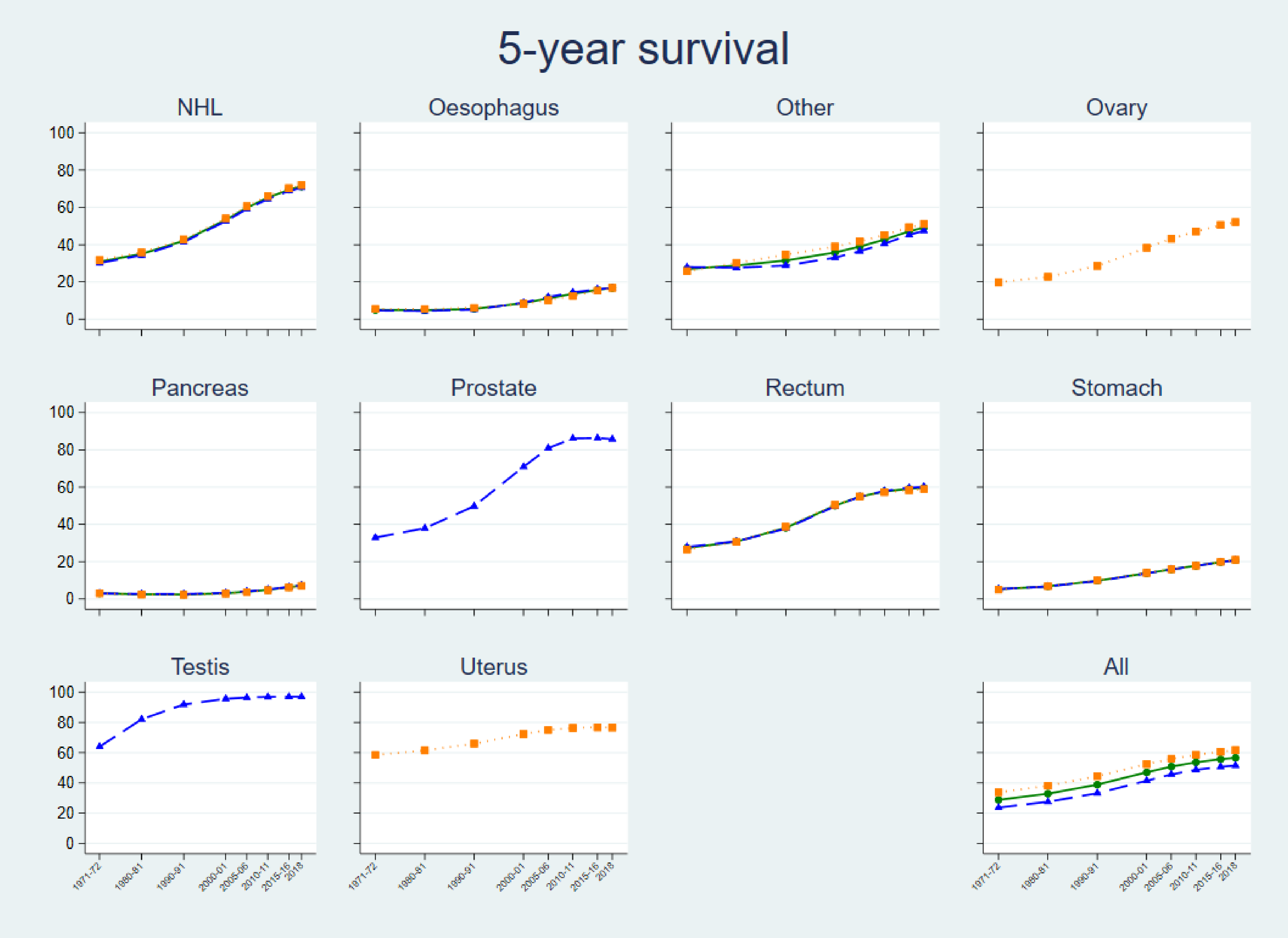

Appendix Figure 3. Trends in age-standardised seven-year net survival (%) for all cancers combined (Cancer Survival Index), and for 22 cancers, for men (blue line), women (orange line) and both sexes combined (green line), in England and Wales, for selected periods during 1971-2018

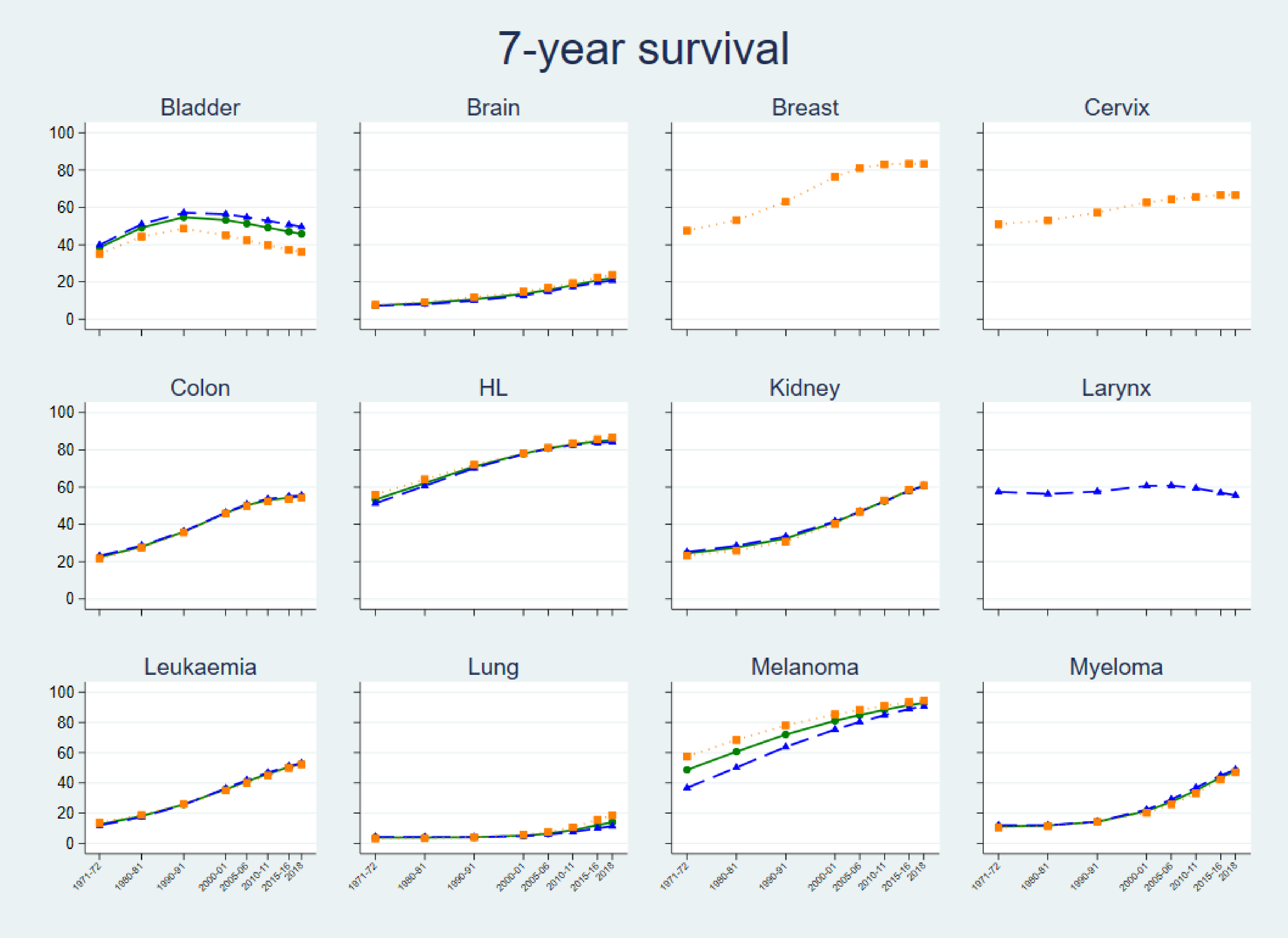

Appendix Figure 3. Trends in age-standardised seven-year net survival (%) for all cancers combined (Cancer Survival Index), and for 22 cancers, for men (blue line), women (orange line) and both sexes combined (green line), in England and Wales, for selected periods during 1971-2018

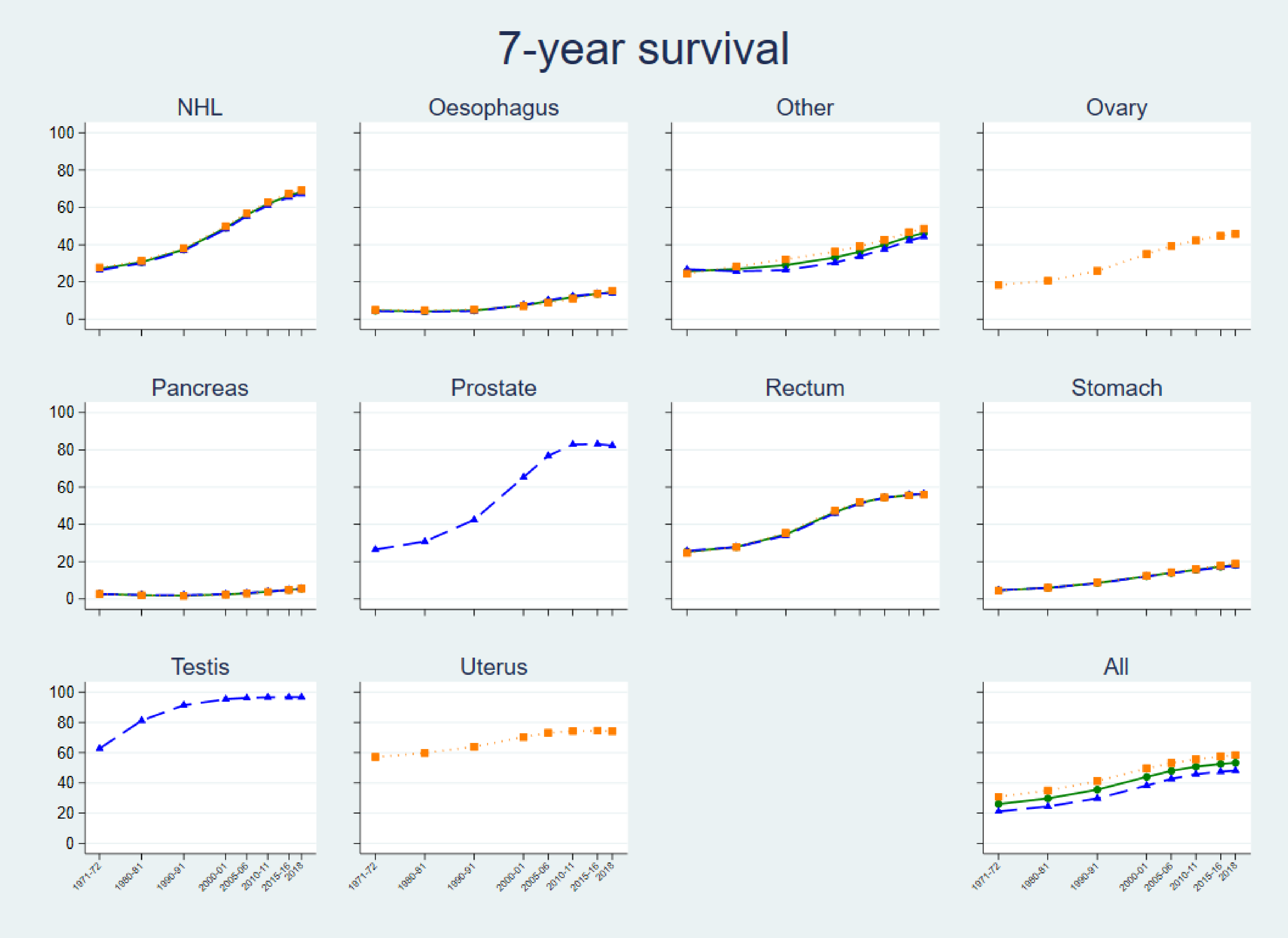

Supplement: Web-appendix [file mmc1.pdf]
